# Brazilian Guideline on Menopausal Cardiovascular Health – 2024

**DOI:** 10.61622/rbgo/2024rbgo100

**Published:** 2024-10-15

**Authors:** Gláucia Maria Moraes de Oliveira, Maria Cristina Costa de Almeida, Carolina María Artucio Arcelus, Larissa Espíndola, Maria Alayde Mendonça Rivera, Agnaldo Lopes da Silva-Filho, Celi Marques-Santos, César Eduardo Fernandes, Carlos Japhet da Matta Albuquerque, Claudia Maria Vilas Freire, Maria Cristina de Oliveira Izar, Maria Elizabeth Navegantes Caetano Costa, Marildes Luiza de Castro, Viviana de Mello Guzzo Lemke, Alexandre Jorge Gomes de Lucena, Andréa Araujo Brandão, Ariane Vieira Scarlatelli Macedo, Carisi Anne Polanczyk, Carla Janice Baister Lantieri, Eliana Petri Nahas, Elizabeth Regina Giunco Alexandre, Erika Maria Gonçalves Campana, Érika Olivier Vilela Bragança, Fernanda Marciano Consolim Colombo, Imara Correia de Queiroz Barbosa, Ivan Romero Rivera, Jaime Kulak, Lidia Ana Zytynski Moura, Luciano de Mello Pompei, Luiz Francisco Cintra Baccaro, Marcia Melo Barbosa, Marcio Alexandre Hipólito Rodrigues, Marco Aurelio Albernaz, Maria Sotera Paniagua de Decoud, Maria Sanali Moura de Oliveira Paiva, Martha Beatriz Sanchez-Zambrano, Milena dos Santos Barros Campos, Monica Acevedo, Monica Susana Ramirez, Olga Ferreira de Souza, Orlando Otávio de Medeiros, Regina Coeli Marques de Carvalho, Rogerio Bonassi Machado, Sheyla Cristina Tonheiro Ferro da Silva, Thais de Carvalho Vieira Rodrigues, Walkiria Samuel Avila, Lucia Helena Simões da Costa-Paiva, Maria Celeste Osorio Wender

**Affiliations:** 1 Universidade Federal do Rio de Janeiro Rio de Janeiro RJ Brazil Universidade Federal do Rio de Janeiro (UFRJ), Rio de Janeiro, RJ – Brazil; 2 Centro Universitário de Belo Horizonte Belo Horizonte MG Brazil Centro Universitário de Belo Horizonte, Belo Horizonte, MG – Brazil; 3 Centro Cardiovascular de Sanatorio Galicia Montevideo Uruguay Centro Cardiovascular de Sanatorio Galicia,Montevideo – Uruguay; 4 Hospital Santa Izabel Salvador BA Brazil Hospital Santa Izabel, Salvador, BA – Brazil; 5 Hospital Municipal de Salvador Salvador BA Brazil Hospital Municipal de Salvador, Salvador, BA – Brazil; 6 Universidade Federal de Alagoas Maceió AL Brazil Universidade Federal de Alagoas (UFAL), Maceió, AL – Brazil; 7 Universidade Federal de Minas Gerais Belo Horizonte MG Brazil Universidade Federal de Minas Gerais (UFMG), Belo Horizonte, MG – Brazil; 8 Universidade Tiradentes Aracaju SE Brazil Universidade Tiradentes (UNIT),Aracaju, SE – Brazil; 9 Hospital São Lucas Rede D’Or São Luis Aracaju SE Brazil Hospital São Lucas Rede D’Or São Luis, Aracaju, SE – Brazil; 10 Faculdade de Medicina do ABC Santo André SP Brazil Faculdade de Medicina do ABC, Santo André, SP – Brazil; 11 Hospital Santa Joana Recife Recife PE Brazil Hospital Santa Joana Recife, Recife PE – Brazil; 12 EMCOR – Diagnósticos do Coração LTDA Recife PE Brazil EMCOR – Diagnósticos do Coração LTDA, Recife PE – Brazil; 13 Hospital Barão de Lucena Recife PE Brazil Hospital Barão de Lucena,Recife PE – Brazil; 14 Universidade Federal de São Paulo São Paulo SP Brazil Universidade Federal de São Paulo (UNIFESP), São Paulo, SP – Brazil; 15 Centro Universitário do Estado Pará Belém PA Brazil Centro Universitário do Estado Pará (CESUPA), Belém PA – Brazil; 16 Faculdade IPEMED de Ciências Médicas Belo Horizonte MG Brazil Faculdade IPEMED de Ciências Médicas, Belo Horizonte MG – Brazil; 17 Cardiocare Clínica Cardiológica Curitiba PR Brazil Cardiocare Clínica Cardiológica, Curitiba PR – Brazil; 18 Hospital Agamenom Magalhães Recife PE Brazil Hospital Agamenom Magalhães, Recife PE – Brazil; 19 Universidade do Estado do Rio de Janeiro Rio de Janeiro RJ Brazil Universidade do Estado do Rio de Janeiro (UERJ), Rio de Janeiro RJ – Brazil; 20 Santa Casa de Misericórdia de São Paulo São Paulo SP Brazil Santa Casa de Misericórdia de São Paulo, São Paulo, SP – Brazil; 21 Hospital de Clínicas da Universidade Federal do Rio Grande do Sul Porto Alegre RS Brazil Hospital de Clínicas da Universidade Federal do Rio Grande do Sul (UFRS), Porto Alegre RS – Brazil; 22 Hospital do Coração São Paulo SP Brazil Hospital do Coração (HCor), São Paulo SP – Brazil; 23 RitmoCheck São José dos Campos SP Brazil RitmoCheck, São José dos Campos, SP – Brazil; 24 Instituto do Coração Hospital das Clínicas FMUSP São Paulo SP Brazil Instituto do Coração (Incor) do Hospital das Clínicas FMUSP, São Paulo SP – Brazil; 25 Universidade Federal de Campina Grande Campina Grande PB Brazil Universidade Federal de Campina Grande, Campina Grande, PB – Brazil; 26 Universidade Federal de Alagoas Maceió AL Brazil Universidade Federal de Alagoas (UFAL), Maceió AL – Brazil; 27 Universidade Federal do Paraná Curitiba PR Brazil Universidade Federal do Paraná (UFPR), Curitiba, PR – Brazil; 28 Pontifícia Universidade Católica do Paraná Curitiba PR Brazil Pontifícia Universidade Católica do Paraná (PUC-PR), Curitiba, PR – Brazil; 29 Universidade Estadual de Campinas Campinas SP Brazil Universidade Estadual de Campinas (UNICAMP), Campinas, SP – Brazil; 30 Hospital Socor Belo Horizonte MG Brazil Hospital Socor, Belo Horizonte, MG – Brazil; 31 Hospital Estadual da Mulher Goiânia GO Brazil Hospital Estadual da Mulher, Goiânia, GO – Brazil; 32 Sanatorio Italiano Assunção Paraguay Sanatorio Italiano, Assunção – Paraguay; 33 Universidade Federal do Rio Grande do Norte Natal RN Brazil Universidade Federal do Rio Grande do Norte, Natal, RN – Brazil; 34 Comité de Enfermedades Cardiovasculares de la Mujer Sociedad Venezolana de Cardiología Caracas Venezuela Comité de Enfermedades Cardiovasculares de la Mujer, Sociedad Venezolana de Cardiología, Caracas – Venezuela; 35 Hospital São Lucas Rede D’Or São Luiz Aracaju SE Brazil Hospital São Lucas, Rede D’Or São Luiz, Aracaju, SE – Brazil; 36 Pontificia Universidad Católica de Chile Santiago Chile Pontificia Universidad Católica de Chile, Santiago – Chile; 37 Hospital Privado Rosario Rosario Argentina Hospital Privado Rosario, Rosario – Argentina; 38 Instituto Universitario Rosario Santa Fe Argentina Instituto Universitario Rosario (IUNIR), Santa Fe – Argentina; 39 Rede D’Or Rio de Janeiro RJ Brazil Rede D’Or, Rio de Janeiro, RJ – Brazil; 40 Ministério da Saúde Brasília DF Brazil Ministério da Saúde, Brasília, DF – Brazil; 41 Hospital Geral de Fortaleza Fortaleza CE Brazil Hospital Geral de Fortaleza, Fortaleza CE – Brazil; 42 Secretaria de Saúde do Estado do Ceará Fortaleza CE Brazil Secretaria de Saúde do Estado do Ceará, Fortaleza CE – Brazil; 43 Faculdade de Medicina de Jundiaí Jundiaí SP Brazil Faculdade de Medicina de Jundiaí, Jundiaí, SP – Brazil; 44 CEMISE Oncoclínicas Aracaju SE Brazil CEMISE Oncoclínicas, Aracaju, SE – Brazil; 45 Universidade Federal de Sergipe Aracaju SE Brazil Universidade Federal de Sergipe (UFS), Aracaju, SE – Brazil; 46 Hospital de Clínicas de Porto Alegre Porto Alegre RS Brazil Hospital de Clínicas de Porto Alegre, Porto Alegre, RS – Brazil

## 1. Introduction

After the publication of the results from the *Women’s Health Initiative* (WHI) clinical trial in 2002 showing more risks than benefits to female health with estrogen (alone or combined with progestin) use to control menopausal signs and symptoms,^1^ there has been a progressive and sustained decline in the prescription of those drugs.^2-4^

In the United States, there was an increase in the prescription of menopausal hormone therapy (MHT) from 16 million in 1966 to 90 million in 1999,^5-7^ so that, by the end of the 1990s, 25% of 45–74-year-old women^8^ and more than 40% of those aged 50-69 years were on that therapy.^5-7^

There is evidence that, after the publication of the WHI study,^1^ the MHT prescription declined from 25% to 11.9% in 2003-2004, reaching 4.7% in 2012, in all demographic groups studied.^9^ It is worth noting that, even after new evidence that MHT could be used for younger women with no additional risk and who were within the first 10 years from menopause onset, there has been no increase in MHT prescription, which is currently found in 4-6% of the women in that phase.^10^

The world population is currently estimated at 8 019 876 189 people, 49.75% of whom are of the female sex and with a life expectancy at birth of 76 years (6 years more than men),^11^ higher access to education and the labor market (despite the indisputable and persistent gender inequality observed), and who tend to suffer with menopausal signs and symptoms for at least one third of their lives. As those women age, they are at increasing risk for cardiovascular (CV) morbidity and mortality,^12,13^ considering that one third of the current female mortality results from ischemic heart disease (IHD) and cerebrovascular disease.^14^

According to Faubion and Shufelt,^10^ the new generations of women will reach menopause with not only more freedom and safety to openly talk about the burden imposed by menopausal signs and symptoms, but more likely to search for solutions. This represents a potential product market valued at an estimated 600 billion dollars. Thus, this substantial number of women needs a health care system prepared for this scenario. In addition, to face the challenge, those authors^10^ state that the science of menopause needs to advance in regard to scientific investigation, education and updating of health professionals in female issues (internal medicine, endocrinology, cardiology, family medicine, and gynecology and obstetrics), creation of state public policies for education in women’s health and care, in addition to the education of employers and organizations’ leaders, who need to adapt the workplace to the women’s needs in that stage life.

In this context, the elaboration, organization, and presentation of this “Guideline on Cardiovascular Menopausal Health”, resulting from the joint work of national [Brazilian Federation of the Gynecology and Obstetrics Societies (in Portuguese, *Federação Brasileira das Associações de Ginecologia e Obstetrícia -* FEBRASGO), Brazilian Association of Menopause (in Portuguese, *Associação Brasileira de Climatério -* SOBRAC), and Brazilian Society of Cardiology (in Portuguese, *Sociedade Brasileira de Cardiologia -* SBC)] and international scientific societies [*Sociedad Interamericana de Cardiología* (SIAC)], as well as several specialties that deal with women’s health, meet all important requirements for educating and/or updating health professionals in the field, consisting of the disclosure of the best scientific evidence currently available on postmenopause and menopause.^15^

**Figure 1.1:**
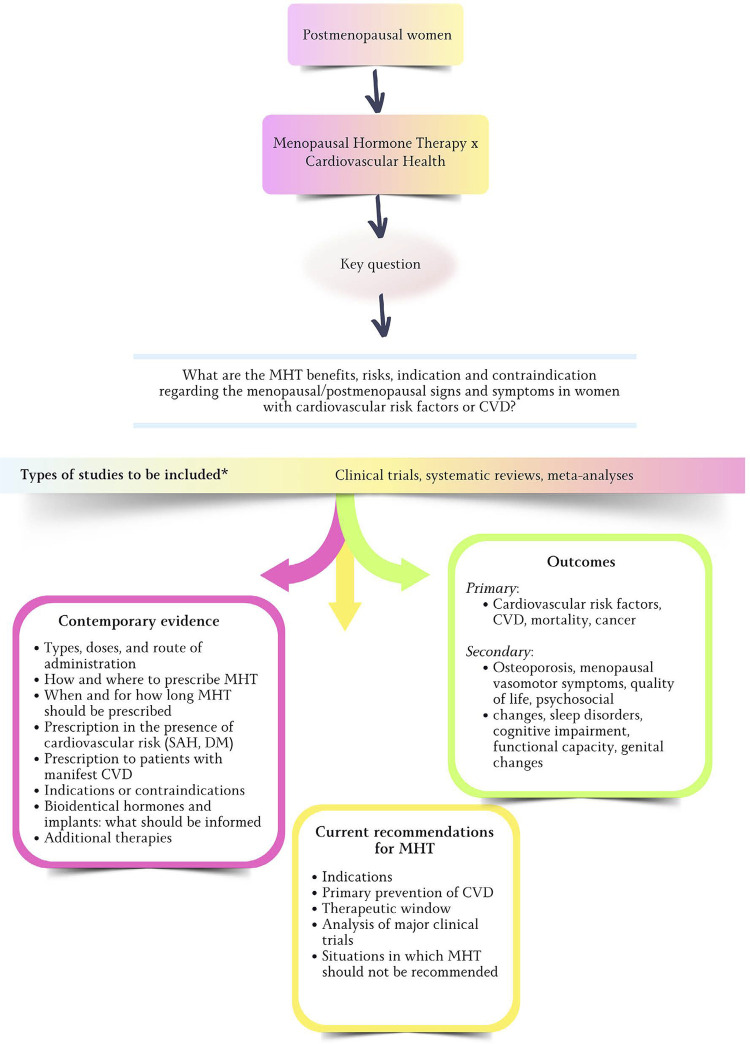
Structure of the systematic review that guided this guideline on menopause and postmenopause. CVD: cardiovascular disease; DM: diabetes mellitus; MHT: menopausal hormone therapy; SAH: systemic arterial hypertension.

The elaboration of this document involved a systematic review (Appendix 1), registered on PROSPERO 2024 CRD42024504299 Available from: https://www.crd.york.ac.uk/prospero/display_record.php?ID=CRD42024504299. The methods used are described in the appendix to this guideline (Figure 1.1).

The following section provides the highlights of each chapter.

### 1.1. Highlights

#### Sex Hormones (Estrogen, Progesterone, Testosterone) and their Functions Throughout Life

Ovarian steroidogenesis begins in puberty when hormones act on secondary sexual characters and regulation of pregnancy. Sex hormones (estrogens, androgens, and progesterone), through their receptors present in the entire female body, act and have specific functions.

Postmenopausal syndrome encompasses the set of symptoms and signs resulting from the interaction between sociocultural, psychological, and endocrine factors. Its diagnosis in women older than 45 years, in the presence of complaints suggestive of hypoestrogenism, requires no confirmation by additional exams.

Estradiol deficiency in menopause contributes to endothelial dysfunction due to loss of its vascular functions, such as NO synthesis increase, antioxidant action, and anti-inflammatory properties.

Changes in the lipid profile, such as increase in total cholesterol, LDL cholesterol, and triglycerides, begin in menopausal transition.

Hypoestrogenism leads to changes in female body fat storage and distribution, with increased central adiposity (android shape) and cardiovascular risk.

#### Relation between Postmenopause/Menopause and Traditional and/or Emerging Cardiovascular Risk Factors

The reduction in the protective function of HDL cholesterol and the increase in the Lp (a) concentration in perimenopause contribute to increase cardiovascular risk. Changes in the glucose metabolism associated with increased central adiposity predispose to the development of diabetes mellitus, which, in the presence of early menopause, leads to higher increase of the cardiovascular risk.

The risk for ischemic heart disease increases in menopause, and worsens the prognosis of women with previous disease, with higher revascularization rates and progression to heart failure.

Women with systemic arterial hypertension have higher incidence of left ventricular hypertrophy in postmenopause, with higher risk of diastolic dysfunction. Isolated systolic hypertension in this phase is related to greater aortic stiffness.

Sedentary lifestyle in postmenopause leads to worse physical fitness and poorer control of cardiovascular risk factors, in addition to higher incidence of fractures and mortality. Smoking increases the risk of early menopause and the likelihood of cardiovascular disease, stroke, osteoporosis, diabetes mellitus, and all-cause mortality.

In menopausal transition, the risk of depression and anxiety is higher. Emotional triggers associated with chronic stress lead to sustained activation of the hypothalamus-hypophysis-adrenal axis, deregulation of metabolic processes, and systemic inflammation, accelerating atherosclerosis and increasing the cardiovascular risk.

#### Relation between Postmenopause/Menopause and Cardiovascular Diseases

Cardiovascular risk stratification in postmenopause is an important tool to identify the major risk factors and risk markers and to implement measures to prevent and reduce women’s mortality. There is no specific risk score for perimenopausal and postmenopausal women, thus, the traditional scores are used and can be refined with the identification of risk-enhancing factors and subclinical atherosclerosis markers.

Women have a lower global burden of atherosclerosis and more coronary microvascular dysfunction. Early menopause is associated with increased mortality from ischemic heart disease.

Elderly women, of Black ethnicity, and lower socioeconomic level have higher incidence of stroke, and arterial hypertension is the major risk factor associated. Arterial hypertension, diabetes mellitus, and smoking have higher negative impact on women, who also have more negative outcomes and increased mortality after stroke.

In postmenopause, systolic and diastolic heart failure and left ventricular concentric remodeling are more frequent, and their incidence is increased in early menopause.

Multiple factors, such as systolic arterial hypertension, obesity, sedentary lifestyle, excessive alcohol intake, valvular heart disease, multiparity, and stroke, suggest a correlation between menopause and increased risk for atrial fibrillation. Early menopause, as well as stress, anxiety, insomnia, and depressive symptoms, is suggested to increase the risk of atrial fibrillation.

#### Menopause and Risk of Morbidity and Mortality from Other Diseases

There is increased cardiovascular risk in postmenopausal women treated for breast cancer, which is worsened by the inadequate control of risk factors and the cardiotoxicity of the treatment.

Women with cancer may have early menopause, depending on the baseline ovarian reserve, gonadotoxicity, and duration of exposure to oncogenic agents (oncological and/or endocrine therapy).

Aging, genetic profile, and systemic vascular disease are the major nonmodifiable risk factors for the development of dementia, whose prevalence is higher among women.

Thyroid dysfunctions are significantly more common in women, and their incidence increases with aging. Manifest and subclinical hyperthyroidism increase the risk of osteoporosis, especially in postmenopause.

Menopausal estrogen loss leads to negative bone remodeling and bone loss, increasing the risk of osteoporosis. Menopausal hormone therapy should be indicated for women with premature ovarian failure and, in natural menopause, it can be indicated to prevent osteoporosis, especially in the presence of vasomotor symptoms.

#### Cardiovascular Risk and Sex Hormones

Cardiovascular risk stratification needs to include the assessment of gynecological history and use of sex hormones throughout life.

Early menarche, polycystic ovary syndrome, and the use of hormonal contraceptives should be recognized as additional cardiovascular risk factors.

Combined oral hormonal contraception has a protective effect on the cardiovascular system. In anovulatory cycles due to hypoestrogenism and hypothalamic dysfunction, however, the risk of coronary atherosclerosis and cardiovascular events increases.

Supplementation with testosterone should not be indicated to women to improve cardiovascular risk.

Independently of the possible additional effects on cardiovascular risk from gender-affirming hormone therapy, prevention focus should be kept on the classic cardiovascular health pillars.

#### Current Recommendations for Menopausal Hormone Therapy

- The Brazilian Society of Cardiology (SBC), the Brazilian Federation of the Gynecology and Obstetrics Societies (FEBRASGO), and the Brazilian Association of Menopause (SOBRAC) recommend the use of menopausal hormone therapy for symptomatic menopausal women without contraindications. (**Strength of recommendation in FAVOR. STRONG recommendation. Level of certainty: HIGH**).
- This therapy consists in the administration of different sex hormones that should be individualized according to the risks and benefits of each woman. The many formulations, doses, and administration routes of hormonal therapy have high efficacy to relieve postmenopausal symptoms. (**Strength of recommendation in FAVOR. STRONG recommendation. Level of certainty: HIGH**).
- The menopausal hormone therapy should be initiated in the “window of opportunity”, that is, within 10 years of menopause onset and/or before the age of 60 years**. However, initiating the menopausal hormone therapy after the age of 60 years or more than 10 years after menopause onset can elevate the absolute risk of coronary artery disease, venous thromboembolism, and stroke. (**Strength of recommendation in FAVOR. STRONG recommendation. Level of certainty: HIGH**).
- There is no indication to start menopausal hormone therapy aiming at primary cardiovascular prevention in multiple scenarios. (**Strength of recommendation in FAVOR. STRONG recommendation. Level of certainty: HIGH**).

#### Contemporary Evidence of Hormonal Therapy in Women

Menopausal women with risk factors for cardiovascular disease need a thorough assessment before initiating menopausal hormone therapy.

Women with controlled systemic arterial hypertension and moderate to intense vasomotor symptoms can use menopausal hormone therapy through any route, but transdermal estrogen should be preferred in the presence of obesity, dyslipidemia, diabetes mellitus, and metabolic syndrome. Micronized progesterone (oral or vaginal route) is recommended for women without hysterectomy.

Systemic menopausal hormone therapy is not recommended for women with manifest cardiovascular disease, previous history of acute myocardial infarction or stroke. Transdermal menopausal hormone therapy is recommended for women with previous history of venous thromboembolism, depending on the factor causing the event.

For women with contraindication to or who do not want to undergo menopausal hormone therapy, nonhormonal therapies can help relieve vasomotor symptoms.

Compounded or bioidentical hormones or hormonal pellets are not recommended because of the lack of scientific evidence about their efficacy and safety.

#### Menopause and Woman in the Job Market – Difficulties and Opportunities for Improvement

Women are a large part of the global workforce, and almost half of them are in peri- or postmenopause.

Menopausal symptoms hinder their quality of life, as well as their work performance and attendance. Thus, employers need to be aware of the discomfort caused by those symptoms, providing a humanized and comfortable work environment.

Institutional policies should be created to support menopausal working women (education on the topic, medical appointments when required, adaptations of the work environment).

Promoting discussions on the theme with the leaderships is necessary, in the search for solutions for the problems presented.

Measures, such as flexible work schedules, more ventilated areas closer to toilets, and lighter and comfortable uniforms, are cost-effective. These measures should be prioritized in the employers’ policies for peri- or postmenopausal working women.

Menopause and Postmenopause in Latin America – Current Situation, Challenges, and Opportunities for Intervention

In low/middle-income countries, there is an increase in the prevalence of premature ovarian failure (before age 40 years) and early menopause (before age 45 years), which are considered risk factors for cardiovascular disease and mortality.

The mean age at menopause onset in Latin America is 47.24 years, with progressive elevation in the prevalence of premature ovarian failure and early menopause.

Vasomotor symptoms are one of the most prevalent symptoms (55%) in Latin American women during menopausal transition, being usually severe in a large part of that population.

In addition to vasomotor symptoms, sleep disorders, urogenital disorders, muscle/joint pains, and mood swings (depression, anxiety, irritability) are frequent and impair the quality of life of women transitioning through menopause and postmenopause.

In Latin America, menopausal hormone therapy is prescribed for 12.5% of menopausal women (oral, 43.7%; transdermal, 17.7%), while alternative therapies are used by 19.5%.

## 2. Sex Hormones (Estrogen, Progesterone, Testosterone) and their Functions Throughout Life

During the intrauterine life, between the sixth and eighth gestational weeks, in a female chromosomal sex embryo, 46XX, there is differentiation of the bipotential, embryonic gonads into ovaries. In the absence of the Y chromosome, the fetus develops ovaries and, in the absence of testosterone levels similar to male ones, the female phenotype appears.^16^ The ovaries begin steroidogenesis during puberty and their hormones, mainly estradiol and progesterone, are responsible for the development of secondary sexual characters and regulation of pregnancy.^17^

From the endocrinological viewpoint, the first signal of puberty is provided by the adrenal glands (adrenarche). With the maturation and growth of the adrenal reticular zone, there is an increase in adrenal androgens, dehydroepiandrosterone (DHEA) and dehydroepiandrosterone sulphate (DHEAS), which will result in testosterone increase. This increase accounts for the maturation of apocrine sweat glands, leading to adult body odor, and development of acne and pubic and axillary hairs. Thus, pubic hairs develop independently of the activation of the hypothalamus-hypophysis-gonadal axis.^18,19^

From puberty on, with the activation of the hypothalamus-hypophysis axis, the ovaries will secrete estrogens, especially estradiol, by the granulosa cells of the follicles, and that synthesis requires the production of androgens, especially testosterone, by the theca cells. In the first 1.5-2 years of ovarian activity, the cycles are anovulatory, thus, no progesterone is produced. Estradiol stimulates the development of the breasts (thelarche), skeletal growth, and development of the internal (uterus, uterine tubes, and upper segment of the vagina) and external (vulva and lower third of the vagina) genital organs, which culminates in the beginning of the menses (menarche). When the ovarian cycles become ovulatory, the corpus luteum resulting from ovulation begins to secrete progesterone along with estradiol. Progesterone is responsible for the changes, mainly endometrial ones, necessary for maintaining pregnancy.^18^

There are receptors for the sex hormones (estrogens, androgens, and progesterone) in almost all tissues and organs of the female body. Thus, those hormones act and have specific functions in the entire female body.

Estrogens are known to play a crucial role in the coordination of several neuroendocrine events that control sexual development, sexual behavior, and reproduction. Estradiol is fundamental to the sexual differentiation of the brain. It organizes neural circuits and regulates the apoptosis of neurons leading to long-term differences in the female brain. In addition, estradiol prevents the death of neuronal cells in a variety of models of brain injury, modulates learning and memory, promotes the formation of synapses, and influences the synthesis of neurotransmitters and cellular apoptosis. Testosterone, acting on the brain, seems to regulate reproduction, sexuality, and emotional behaviors in both sexes in a different context related to gender. Progesterone, by acting on the central nervous system, has a hypnotic/sedative, anxiolytic, and anesthetic/analgesic effect.^18,20,21^

Estradiol has a positive cardioprotective effect through its influence on endothelial, myocardial, vascular, and metabolic functions. Coronary and peripheral vessels have estrogen receptors that allow estradiol to play a role in vascular regulation. Estrogen stimulates the synthesis of nitric oxide (NO) through genomic and nongenomic effects, causing vasodilation. The sex hormones influence the mechanisms involved in body pressure (BP) regulation. Estrogens stimulate the synthesis of factors related to BP reduction. Estrogens, but not androgens, induce favorable effects on the kidneys, which influence BP levels in the long run.^22,23^

Estrogens influence the vascular effects of LDL cholesterol (LDL-c). Estradiol, which is a phenol with antioxidant properties, prevents the oxidation of LDL-c and VLDL cholesterol (VLDL-c), and protects the vasculature against the deleterious effects of the lipids. Estradiol attenuates the accumulation of minimally modified LDL-c and oxidized LDL-c on the arterial wall and prevents LDL-c oxidation and accumulation, mediated by the tumor necrosis factor α, on the arterial wall. In addition, estradiol increases the expression of the LDL-c receptor, increases the VLDL-c clearance, reduces the LDL-c production, decreases the size of LDL-c particles, and increases light and dense LDL-c clearance.^24^

The bone remodeling process, which maintains the skeleton healthy, can be considered a preventive maintenance program, continuously removing older bones and replacing them with new bone. Estrogens are essential to promote balance between bone remodeling, resorption, and formation events.^25^

Therefore, since puberty and during the entire women’s reproductive phase (menacme), the sex hormones have specific and fundamental effects on not only the reproductive system, but all organs and systems of the female body. Always consider estrogens, especially estradiol, to be the main actors, progesterone to be essential to maintain pregnancy, and testosterone to be a supporting actor in some specific functions.

### 2.1. Menopausal Hormone Changes

Women are born with their complete set of follicles, around 1-2 million. At the beginning of puberty, the mass of germ cells has already decreased to 300-500 thousand units. Over the following 35-40 years of the reproductive lifespan, 400-500 will be selected to ovulate, and the primary follicles will be depleted by menopause onset, when only a few hundred will remain.^16,17^

During the reproductive period, the pool of oocytes (follicles) is gradually depleted through ovulation and atresia (apoptosis – programmed cell death). The decline in the pool of oocytes results in the smaller secretion of inhibin B, reducing the negative ovarian feedback on the follicle-stimulating hormone (FSH). The resulting increase in FSH level leads to higher follicle recruitment and accelerated follicle loss, with preservation of estradiol levels at the beginning of menopausal transition (MT). When women are in their 40s, anovulation becomes more prevalent due to the reduced quality and capacity of the aged follicles, and, thus, there is no progesterone production. When all ovarian follicles are depleted, the ovary cannot respond even to elevated FSH levels and, thus, estrogen levels decline. The postmenopausal period is characterized by elevated FSH levels (> 30 mUI/mL) and low estradiol levels (< 30 pg/mL).^17^

Postmenopausal ovary secretes mainly androstenedione and testosterone. After menopause, the circulating level of androstenedione is around half of that observed before menopause. Most of that postmenopausal androstenedione derives from the adrenal gland, and only a small amount is secreted by the ovary, although androstenedione is the major steroid secreted by the postmenopausal ovary. Testosterone production decreases approximately 25% after menopause, but the postmenopausal ovary in most women, but not in all of them, secretes more testosterone than the premenopausal ovary.^17,26^

The circulating level of estradiol after menopause is approximately 10–20 pg/mL, most of which derives from the peripheral conversion of estrone, which derives mainly from the peripheral conversion of androstenedione. The circulating level of estrone in postmenopausal women is higher than that of estradiol, approximately 30–70 pg/mL. The mean postmenopausal production rate of estrogens is approximately 45 μg/24 hours, and almost all of them derive from the peripheral conversion of androstenedione. The androgen/estrogen proportion changes drastically after menopause because of the more marked decline of estrogen, when the appearance of mild hirsutism is common, reflecting that marked change in the proportion of sex hormones.^17,26^

### 2.2. Definition and Classification

Natural menopause is defined as the date of the last menstrual bleeding episode of a woman.^27^ On average, it occurs at the age of 51 years, and in 90% of the women, it occurs between the ages of 45 years and 55 years.^28^ Spontaneous menopause between the ages of 40 years and 45 years occurs in 5% of the women and is known as early menopause.^29^ Induced menopause is the interruption of menses that occurs after surgical bilateral oophorectomy or the iatrogenic loss of ovarian function due to chemotherapy (CTX) or radiotherapy.^29^ Premature ovarian failure (POF) is a syndrome that results from the loss of ovarian activity before the age of 40 years,^30^ which affects approximately 1% of the women.^31^ The term “premature menopause” can be used to refer to cases of definitive menopause before the age of 40 years, such as those resulting from bilateral oophorectomy.^29^ The term “menopausal transition” refers to the period of life when changes in the menstrual cycle occur due to ovarian function decline, starting with variation in the menstrual cycle duration and ending with the last menstrual bleeding episode.^29^

**Figure 1.2:**
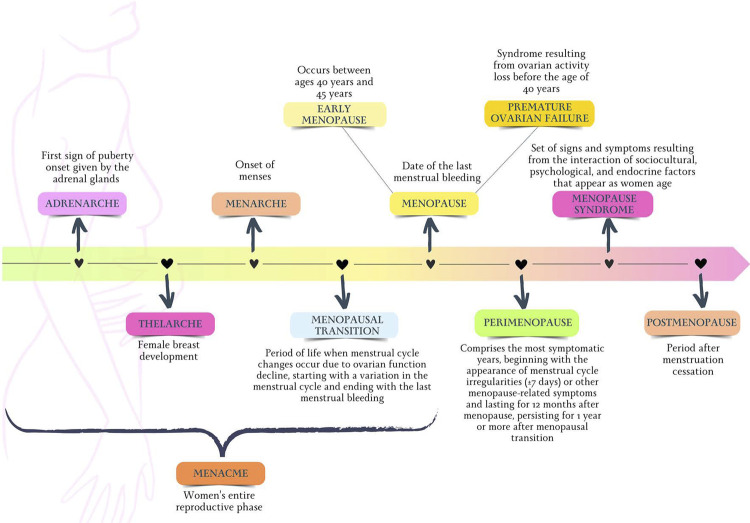
Nomenclature related to the women’s life cycle used in this guideline.

**Figure 2.1:**
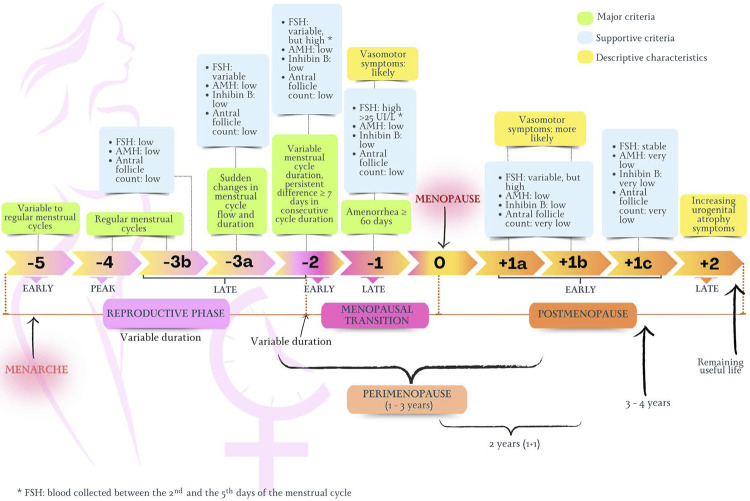
STRAW system for classification of the women’s reproductive stages. AMH: antimüllerian hormone; FSH: follicle-stimulating hormone.

The term “postmenopausal syndrome” encompasses the set of symptoms and signs resulting from the interaction between sociocultural, psychological and endocrine factors that appear as a woman ages.^27^Figure 1.2 illustrates the nomenclature related to women’s life cycles used in this Guideline, from puberty to the end of reproductive life. To standardize the definition of the several stages of reproductive aging, the STRAW (Stages of Reproductive Aging Workshop) system was created.^32^ Based on patterns of symptoms and laboratory findings, the STRAW system classifies reproductive aging in the following phases: reproductive, MT, and postmenopause. Figure 2.1 illustrates details of the STRAW system.^32^

### 2.3. Clinical and Laboratory Diagnosis

The aging process leads to progressive ovarian failure, resulting in the interruption of the ovulatory cycles and end of menstrual bleeding. Frequently, women seek care because of changes in the menstrual cycle during the MT. Because of the reduction in inhibin B production by the ovaries by the end of the fourth decade of life, an increase in the serum concentrations of FSH and estradiol can be observed at the beginning of the cycle, resulting in shortening of the follicular phase. In addition, the quality of the corpus luteum worsens, leading to a decline in progesterone levels in the secretory phase. Shortening of the interval between the menses is one of the first signs of ovarian function decline.^33^

Over the years, the process of follicular depletion persists, and anovulation becomes increasingly common. Because of the lack of progestational effect, the interval between the menses widens, reaching 40-50 days. This mean increase in the interval between the menses occurs around the age of 47 years.^33^ Amenorrhea episodes become longer, intercalated by menstrual bleeding episodes of variable volume. This pattern of menstrual bleeding can persist for a period of one to three years before menopause.^33^

Vasomotor symptoms (VMS), also known as hot flashes, are the most common symptoms related to MT. These symptoms involve sudden sensation of heat in the central body region, especially face, thorax, and neck, with a mean duration of 3-4 minutes.^34^ These episodes are often accompanied by an increase in heart rate, peripheral vasodilation, skin temperature elevation, and sweating. When occurring at dawn, they can be associated with sleep disorders, such as insomnia.^35^ Moderate/severe VMS occur in up to 80% of women.^36^ However, only 20-30% of them seek medical care.^33^ At the beginning of the ovarian function decline, the VMS can be mild, occurring at the lowest estradiol secretion, during the late luteal and initial follicular phases. The occurrence of VMS increases significantly during MT, reaching approximately 40% in early MT and increasing to 60-80% during late MT and the initial postmenopausal stages.^37^ In late postmenopause, the VMS tend to decrease; however, up to 30% of the women can experience moderate/severe VMS 10 years after menopause.^36^

Characterization of the menopause date is performed retrospectively after 12 months of amenorrhea in a woman at the expected age for MT.^23^ Diagnosis of postmenopausal syndrome is established by use of detailed anamnesis, complemented by a thorough physical exam.^38^ For women over the age of 45 years with complaints suggesting hypoestrogenism, such as VMS and typical changes of the menstrual pattern (less frequent uterine bleeding), the diagnosis of postmenopausal syndrome is clinical and requires no confirmation with other complementary tests.^38^ In cases with doubts regarding the symptomatology due to a drop in ovarian production of estradiol, measuring FSH levels in the initial follicular phase can be useful to confirm the diagnosis. Levels over 25 mUI/mL can indicate the onset of MT. However, it is worth noting that the daily concentrations can vary considerably in this phase. When necessary, two measurements should be taken at an interval of 4-6 weeks.^38^ In addition, most women on hormonal contraception based on progestogens alone will have changed bleeding patterns or amenorrhea, hindering the precise identification of the menopausal *status*. If necessary, women on hormonal contraception with progestogens alone can undergo serum measurements of FSH to assess their menopausal *status*.^39^ Levels > 25 mUI/mL are attributable to ovarian function decline. However, progestogens alone, such as depot medroxyprogesterone acetate and hormonal pellets, can suppress FSH, thus a woman on such medications can be in the perimenopause without showing increased FSH levels.^39^ The ideal moment to measure the FSH levels of a woman on depot medroxyprogesterone acetate is right before a new administration of the medication.^40^ Women on combined hormonal contraception have significantly suppressed FSH levels, even during the hormone-free phase, which make them inappropriate to provide information on the menopausal *status*. In addition, the VMS are less frequent due to the effects of the estrogen component of the contraceptive.^39^ For women on combined contraceptives who require FSH measurement, medication should be suspended 2-4 weeks before blood withdrawal.^33^

Bleeding patterns that do not meet those of ovarian function decline, such as very often bleeding, with increased volume and clots, require endometrial investigation with ultrasonography and/or endometrial biopsy.^38^ For women aged less than 45 years complaining of abnormal uterine bleeding of irregular pattern and less frequent menstrual cycles, even when the clinical findings suggest hypoestrogenism, additional investigation is recommended to assess the symptoms and exclude other causes of menstrual irregularity, such as pregnancy, thyroid disorders, and hyperprolactinemia.^35^

### 2.4. Relation to Cardiovascular Mortality

Coronary artery disease (CAD) is the most common cause of death in postmenopausal women, more frequent than breast cancer or any other gynecological cancer. The traditional risk factors (RFs) for CAD include age, smoking, sedentary lifestyle, unhealthy diet, elevated body mass index (BMI), systemic arterial hypertension (SAH), diabetes mellitus (DM), dyslipidemia (DLP), and family history of CAD. The prevalence of CAD among premenopausal women is low, probably because of the estrogens’ protective effects in women.^41^ There is a marked increase in the incidence of CAD in women after menopause, usually found 10 years after the last menstrual period.^42^

Menopause per se is unlikely to account for that change, and other RFs, such as DLP, insulin resistance, body fat redistribution, and SAH, can cause metabolic and vascular changes, contributing to increase the risk for CAD and cardiovascular disease (CVD). Those clinical situations can be related to peripheral adverse effects of the endothelial function.

Vascular aging is characterized by progressive arterial stiffening with decline in the vasodilation ability, which progresses differently in men and women. At the beginning of menopause, it occurs quickly, differently from the gradual loss of the vascular function observed as age advances. Endothelial dysfunction and vascular aging contribute to the development of SAH and atherosclerosis, favoring the increase of CVD in menopause.^43,44^

Estradiol is crucial to maintain normal endothelial function. Estradiol increases NO synthesis by the vascular endothelium, which thus spreads to the interior of smooth muscle cells, causing their relaxation. This is called endothelium-dependent vasodilation, whose loss is a characteristic of endothelial dysfunction. Estradiol preserves the endothelial function, and the decline of ovarian hormones with reproductive aging in menopause quickly affects endothelium-dependent vasodilation.^45^

Studies have shown that estradiol has antioxidant and anti-inflammatory properties. Estrogen deficiency regulates positively oxidative stress or systemic inflammation, leading to endothelial function decline.^46^ Thus, estrogen has multiple functions, such as antioxidant, increase in NO synthesis, and anti-inflammatory properties. Its deficiency in menopause contributes to endothelial dysfunction.^47^

Changes in the women’s lipid profile begin in the MT period, with increases in total cholesterol (TC), LDL-c, triglycerides (TG). The *Women’s Health Across the Nation* (SWAN) was a prospective study of MT in Caucasian women and representatives of minorities (Afro-American, Hispanic, Japanese, Chinese women) who were not on hormone therapy. That study provided evidence that MT is related to adverse lipid profiles. It showed that TG, LDL-c, and apolipoprotein-B increase already within 1 year from the last menstrual period, independently of the age at which that occurs. All these factors are directly related to endothelial dysfunction and lead to atherosclerosis. An increase in LDL-c during MT is related to the appearance of carotid plaques in postmenopause.^48-50^ Such changes differ from the linear changes related to chronological aging.

Metabolic syndrome (MS) is defined as the coexistence of several metabolic RFs, such as SAH, DLP, glucose intolerance, and central adiposity. Estradiol plays an important role in fat storage and distribution. Before menopause, fat is mainly deposited on the thighs and hip. Women tend to gain weight (total body fat) during midlife because of the aging chronology. However, during MT, women undergo a change in body composition and fat distribution, with central adiposity increase.^51^ The MT can, thus, contribute to increase abdominal fat, insulin resistance, DM, and inflammatory diseases, leading to the development or worsening of MS in women.^51-53^

The progression of the atherosclerotic process seems to be the final result of a complex interaction between CVD, RFs, and their aggravation during the perimenopausal period. The cardiovascular risk (CVR) increase in menopause results from the important changes in the CV system physiology that affect the peripheral and cardiac vasculatures, as well as the cerebrovascular system. Changes in the lipid profile, vascular stiffness, metabolic parameters, and oxidative stress contribute to worsen the CVR of women during the MT.

**Figure 2.2:**
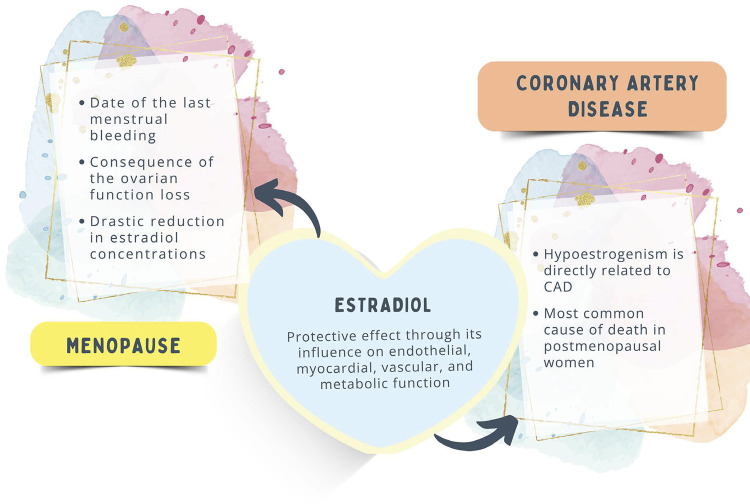
Hypoestrogenism and coronary artery disease (CAD).

The treatment strategies should include strict control of cardiovascular risk factors (CVRFs) to prevent the progression of atherosclerotic disease in menopausal women. Figure 2.2 shows the interactions between hypoestrogenism and CAD.

## 3. Relation Between Postmenopause/Menopause and Traditional and/or Emerging Cardiovascular Risk Factors

### 3.1. Introduction

By 2025, there will be more than 1.1 billion postmenopausal women in the world, representing 12% of the entire world population. With population aging, women are likely to spend half of their lives in that phase. Menopausal transition is a landmark in a woman’s life, associated with bothersome symptoms, such as hot flashes, night sweats, sleep and mood disorders, which jeopardize quality of life. In addition, menopause is associated with chronic diseases, such as CVD, DM, neoplasms, and osteoporosis.^54^

Would the increased frequency of CVD in perimenopause be due to chronological or ovarian aging? Recent data from longitudinal studies have shown that menopause-related factors, such as earlier age of menopause onset and surgical menopause, are related to more CV outcomes. In addition, perimenopause is associated with cardiometabolic RFs, such as body composition, visceral fat accumulation, SAH, DLP, MS, chronic stress, sedentary lifestyle, smoking, and social determinants of health.^54,55^

Menopause transition is a time of acceleration of the CVD risk, thus, monitoring the health of women during midlife is important, because that is a critical window for the implementation of early intervention strategies to reduce the CVD risk. Thus, discussing the CVRFs associated with MT, perimenopause, and postmenopause is extremely important.^55^

### 3.2. Hypertension

Systemic arterial hypertension is the most prevalent and modifiable CVRF and the one associated with the highest rates of death and DALYs (Disability-Adjusted Life Years) in Brazil and worldwide for both sexes. The SAH prevalence increases with age in both sexes, but that increment is accentuated in women after menopause and older than 65 years, exceeding that of men in the same age range.^56^ The SAH that occurs in that phase of women’s lives seems to be more sensitive to salt overload, being more often associated with MS and the appearance of adverse effects from medications as compared to SAH in men at the same age.^57^

According to data from Vigitel 2021, regarding self-reported SAH, the highest prevalence of SAH in Brazil, 61% (confidence interval - 95% CI, 59.0-63.0), was observed in individuals aged 65 years and older, and, in that age group, women had higher prevalence than men, 63.7% (95% CI, 61.6-65.8) and 57.1% (95% CI, 53.4-60.7), respectively.^58^In that age range, less than half of the postmenopausal women had controlled SAH.^58^

Postmenopausal women with SAH have a higher incidence of left ventricular (LV) hypertrophy and greater risk of developing diastolic dysfunction as compared to younger adult women. Isolated systolic SAH in postmenopausal women is related to greater aortic stiffness probably caused by proliferation of smooth muscle cells, collagen accumulation, and increased levels of vasoconstricting molecules in the blood vessel walls due to lack of the estrogen’s protective effect.^59,60^

The lack of estradiol can negatively interfere with vasodilation due to effects on the renin-angiotensin-aldosterone system (RAAS), NO system, endothelin, and immune system. In addition, the lack of estradiol can affect NO bioavailability, due to the reduced superoxide dismutase activity, and the humoral and cellular immune responses.^59,60^ However, the decline in progesterone levels can be at least partially associated with the occurrence of SAH in postmenopausal women, given that progesterone acts as a vasoactive hormone, preventing the noradrenaline-induced vasoconstriction, acting directly on the vascular smooth muscle cells. In addition, in postmenopausal women, low levels of DHEAS, androgen, and the precursor of steroid hormones were associated with higher CV and all-cause mortality.^61^ Two crucial changes in autonomic regulation during menopause that can propitiate the development of SAH are worth noting: increase of the central sympathetic flow and increase of the adrenergic sensitivity in peripheral blood vessels.^55^

Although estrogen plays a protective role in premenopausal women, the administration of exogenous estrogens to menopausal women affects neither BP nor the risk for CV outcomes. After the MHT onset, BP should be monitored and, if no proper BP control is achieved, MHT should be suspended.^59^

The absorption, distribution, metabolism, and excretion of anti-hypertensive drugs differ between women and men probably due to the influence of the sex hormones on absorption (P-glycoprotein), distribution volume, cytochrome P450 (CYPs) activity, and renal clearance.^62^ The adverse effects of anti-hypertensive drugs are more often reported in women, especially during menopause, such as cough induced by angiotensin-converting-enzyme inhibitors, ankle edema with calcium blockers, and hypokalemia and hyponatremia with diuretics. These adverse effects can explain the smaller adhesion of menopausal women to the SAH treatment.^57-60,62^

### 3.3. Overweight/Obesity

The physiological and metabolic changes associated with menopause are a direct effect of estrogen deficiency, which affect the lipid metabolism, energy consumption, insulin resistance, and body fat composition, with transition from a gynecoid body shape to an android one, with increased accumulation of abdominal and visceral fat, diagnosed by the waist circumference measure and waist-to-hip ratio. These changes were associated with increased metabolic and CV risks, as well as the risks related to diabetes type 2 (DM2), LDL-c, and endometrial and breast cancers.^63^

Results from long-term cohort studies with a large number of women, such as SWAN and WHI, have suggested that the increase of obesity in postmenopause, measured by use of BMI, is consequent to age and occurs in both previously obese and non-obese women after menopause. No or mild association between obesity and late menopause onset has been observed.^64,65^Postmenopausal women with obesity have a four-fold increased risk of CV mortality.^64,65^The longitudinal studies SWAN and WHI have shown ethnic differences in the physical and metabolic changes that occur during postmenopause.^64,65^

Studies have shown that perimenopausal women with obesity have less intense VMS than normal-weight women, possibly due to lower levels of estradiol and FSH, aromatization of androgens to estrogens in the adipose tissue, which down-regulate the hypothalamus and hypophysis, decreasing FSH and the ovarian secretion of estrogen. Other symptoms of postmenopausal obese women, particularly those associated with increased abdominal circumference, are apnea and other sleep disorders and genitourinary symptoms.^66,67^

Women with obesity are more likely to have symptoms during perimenopause and require MHT. However, the use of MHT is associated with a higher risk of venous thromboembolism (VTE), CV complications, and breast and endometrial cancers, especially in obese women. Thus, strict risk-benefit assessment of MHT is required, even when indicated. In this case, the use of patches with micronized progesterone and low-dose estrogen is suggested for a short period.^63,68^ Studies have shown that changes in lifestyle prevent perimenopause-associated visceral adiposity and improve the symptoms and cardiometabolic risks.^63,67^

### 3.4. Metabolic Syndrome

Cross-sectional studies have shown that, as compared to premenopausal women, postmenopausal women have significantly more visceral obesity and MS. Meta-analysis performed with articles published between 2004 and 2017 (119 studies, n = 95 115) has shown postmenopausal MS prevalence of 37.17% (95% CI, 35.00%-39.31%). The pooled odds ratio (OR) for MS in postmenopausal women, compared to premenopausal women (23 studies, n = 66 801), was 3.54 (95% CI, 2.92-4.30). The chances of high fasting glycemia (OR 3.51; 95% CI 2.11-5.83), low HDL cholesterol (HDL-c) (OR 1.45; 95% CI, 1.03-2.03), high BP (OR 3.95; 95% CI, 2.01-7.78), high TG (OR 3.2; 95% CI, 2.37-4.31), and increased waist circumference (OR 2.75; 95% CI, 1.80-4.21) were all higher in postmenopausal women than in premenopausal women.^69^

Women in MT tend to have higher peripheral fat deposits accumulating in the gluteofemoral region (“pear shaped”). However, during the menopausal period, fat tends to accumulate centrally, and, in addition to the decline in the estrogen’s protective effect, it contributes to endothelial dysfunction, inflammatory *status* and arterial stiffness, resulting in increased risk for CVD. Moreover, postmenopausal women tend to reach higher levels of TC, LDL-c, TG, and lipoprotein (a) [Lp(a)], but lower levels of HDL-c, as compared to perimenopausal women, which represents a change to a pro-atherogenic and procoagulant lipid profile, strongly related to the increase in visceral fat and other traditional RFs for CVD.^70^

A study using data of 1470 women from the *Atherosclerosis Risk in Communities cohort* (ARIC), with a 10-year follow-up and four visits, has reported gradual increases in the MS severity over time. Black women exhibited more rapid progression in MS severity during the MT and perimenopausal periods than during the postmenopausal period, in which favorable changes were observed in the rate of variation of waist circumference, TG, HDL-c, and glucose. These data suggest that the higher MS prevalence in postmenopausal women can be caused by changes during MT than during postmenopause, suggesting higher CVR from MS in the perimenopausal period.^71^

The presence and severity of MS are associated with an increased risk of DM2 in the perimenopausal period. However, surgical menopause is strongly associated with higher MS incidence. It is worth noting that women with polycystic ovary syndrome (PCOS) have an increased risk of MS during the reproductive years; however, during MT, the risk for MS is similar to that of women without PCOS.^72^

### 3.5. Sedentary Lifestyle

Sedentary lifestyle is one of the CVRFs and independent prognostic marker of mortality.^73,74^Sedentary women have been shown to have worse physical fitness in postmenopause as well as poorer control of other CVRFs as compared to women practicing physical exercises.^75^In the last American Heart Association (AHA) position statement on the construct of CV health, the eighth element has been introduced: sleep health.^76^Studies have shown the association of higher amounts of sedentary time with short sleep duration and poor sleep quality in postmenopausal women.^77^

The results of the WHI study have evidenced a significantly 24% higher risk for incident heart failure (HF) hospitalization in menopausal women with sedentary time longer than 9.5 hours/day.^78^ One reason for that would be the increased activity of the sympathetic nervous system and RAAS.^79^

Cessation of the ovarian function after menopause causes a significant estrogen decline, accelerating bone loss and osteoporosis in 20-30% of women, increasing the likelihood of fractures and mortality. Physical exercises improve muscle strength and balance to prevent falls, restoring self-confidence and coordination, in addition to maintaining bone mass, stimulating bone formation, and reducing bone resorption.^80^ Moreover, physical exercises are recommended to prevent breast cancer.^81^

In all life periods, women should avoid the sedentary behavior to improve their quality of life and reduce the complications from the sedentary lifestyle to health.^82^

According to the World Health Organization (WHO) guidelines, active midlife adults should undertake at least 150 minutes of moderate-intensity, or 75 minutes of vigorous-intensity, aerobic physical activity per week, and associate resistance physical exercises at least twice a week, involving the higher muscle groups.^83^

### 3.6. Smoking

Smoking is considered an important RF for CVD. Studies have shown its association with early age of menopause onset. Female smokers had a two-fold higher risk of developing early menopause, and female ex-smokers had a 15% higher risk of POF and early menopause. A positive relation was observed with intensity, duration, cumulative dose, and early onset of smoking.^84^

Early menopausal age is associated with the increased likelihood of CVD, stroke, osteoporosis, DM, and all-cause mortality. Female smokers die 11 years before those who never smoked and have higher prevalence of CVD and CV and all-cause mortality, reinforcing the need to quit smoking.^64^

### 3.7. Chronic Stress

Chronic stress compromises CV health. Women seem to respond more intensely to the adversities related to their social roles, such as spouse, parent, employee, and caregiver.^85^Some mechanisms are involved in the pathophysiology of CVD, such as sustained activation of the hypothalamus-hypophysis-adrenal axis, deregulation of metabolic processes, and systemic inflammation, contributing to increase BP and in the atherosclerotic process.

Women reporting chronic stress during midlife had significantly higher carotid intima-media thickness in later life than those never reporting a stressful role.^85^Depression was associated with higher coronary artery calcium score (CAC) in postmenopausal women,^86^ being considered an independent RF for CV and all-cause death.^87^Women are at higher risk for depression and anxiety during MT.^88^

The factor “chronic mental stress” has been significantly associated with the increased number of CD63+ platelets and pro-inflammatory platelet bioactivity, being a possible explanation to the relation between mental and somatic disorders in menopause.^89^ The decline in estrogen levels in postmenopausal women increases their susceptibility to Takotsubo cardiomyopathy.^90^

Interventions in lifestyle, such as healthy diet, physical activity, proper sleep duration and quality, as well as practicing meditation and yoga, to reduce chronic psychological stress in menopause emphasize the relationship between mental and CV health.^64^

### 3.8. Dyslipidemia

Menopause results in several lipid disorders due to hormonal changes, such as decline in estrogen levels and increase in circulating androgen levels. The changes in the lipid metabolism and excessive adipose tissue play a fundamental role in the synthesis of excessive fatty acids, adipocytokines, pro-inflammatory cytokines, and oxygen reactive species that cause lipid peroxidation and result in insulin resistance, abdominal adiposity, and DLP.^91^ The population risk attributable to DLP is higher in women as compared to all other CVRFs. However, the benefits of reducing the LDL-c levels to atherosclerosis regression have the same magnitude in women and men.^92^

In addition, there is a bidirectional relation of CVRFs and CV events with the early onset of menopause. Data from the *Framingham Heart Study* have shown that the increase in TC and BP, as well as other CVRFs, before menopause was associated with early menopause, independently of smoking.^93^ Moreover, in a pooled analysis of 177 131 women from 9 studies, a first CVD event before the age of 35 years was associated with doubling the risk of early menopause.^94^

The relation between HDL-c and menopause is particularly relevant. The SWAN study^95^ has suggested that the antiatherogenic function of HDL-c, which is its ability to promote the reverse transportation of cholesterol, can decrease during menopause in association with an apparent inversion of the direction of the association between HDL-c and CVR, with higher levels of HDL-c being associated with less carotid atherosclerosis before menopause, but with higher carotid atherosclerosis after menopause.

Still regarding lipid disorders, the Lp(a) concentration increases during pregnancy and since menopausal onset (around 50 years). In addition, high Lp(a) levels are more common in women than in men after the age of 50 years, which might affect the risk for CVD. All these particularities of lipid disorders in menopause suggest that the current recommendations of the guidelines on DLP might be inadequate for women.^96^

### 3.9. Diabetes Mellitus

In addition to the lipid changes, other metabolic and clinical factors secondary to menopause, such as insulin resistance, fat redistribution, dysglycemia, and DM, contribute to the accelerated risk of aging and CVD. During MT, there are several phenotypical and metabolic changes, which affect body weight, adipose tissue distribution, and energy expenditure, such as insulin secretion and sensitivity. These factors can predispose women to develop DM.^97^

Women with DM are at a 45%-higher risk of developing IHD. In addition, the risk of fatal CAD in women with DM2 is three times higher than that of women without DM2, especially in menopause. The presence of DM also results in a decrease in the revascularization rate and, thus, higher HF occurrence among women as compared to men, which increases in menopause.^92^

Diabetes mellitus coupled with early menopause can result in an even higher CVD risk in women. The risk associated with early menopause (< 45 years) as compared to normal-age menopause has been estimated by Yoshida *et al*. during a 15-year follow-up.^98^ Adjusted hazard ratios (HR) for CV event in early menopause were greater in women with DM *versus* those without DM (CAD: 1.15 *versus* 1.09; stroke: 1.21 *versus* 1.10; CV atherosclerotic disease: 1.29 *versus* 1.10; HF: 1.18 *versus* 1.09).

Another relevant aspect is that, although more prevalent among men, DM2 determines a higher relative increase, although not necessarily absolute, of CVR in women than in men, at all ages. This can be partially related to higher adiposity, considering that women are typically less physically active and have higher BMI than men, in addition to sex-specific RFs for DM, such as PCOS and gestational diabetes.^96^

**Figure 3.1:**
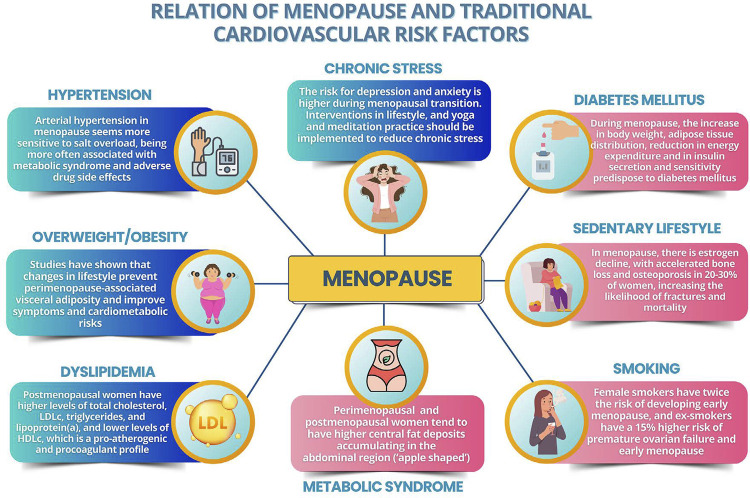
Relation of menopause and traditional cardiovascular risk factors.

Figure 3.1 illustrates the relation of menopause and traditional CVRFs.

### 3.10. Economic Situation and Job

**Figure 3.2:**
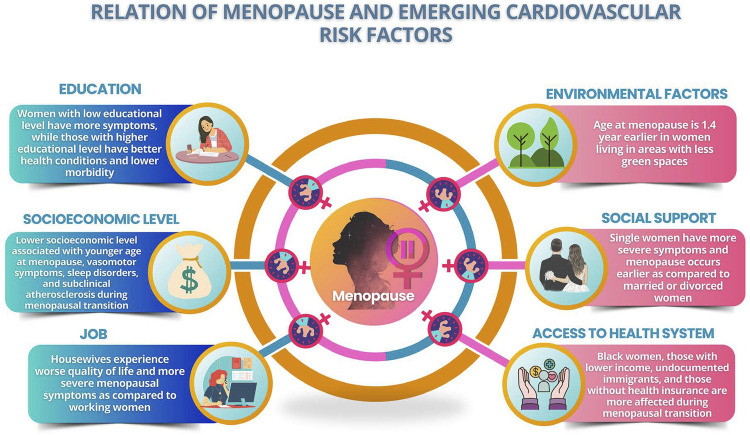
Relation of menopause and emerging cardiovascular risk factors.

Emerging RFs represent a challenge in CVD, and the recognition and quantification of their association with CV outcomes are difficult to currently assess. Their modification involves not only individual, but collective and governmental actions as well. The social risks associated with aging reflect the worsening of social and health care, which are even worse in the elderly when alone. The social determinants of health encompass the social conditions in which people are born, live, and work, and are critical to CV morbidity and mortality (Figure 3.2).^98^

Housewives experience poorer quality of life and more menopausal symptoms as compared to working women. Better financial condition improves the quality of life in menopause, due to the higher access to health services and guidance on the control of menopausal symptoms. Unfavorable socioeconomic conditions are believed to lead to early menopause, and, when occurring during childhood, they can be associated with smoking increase and early menopause.^99,100^

In 2020, almost 20% of middle-aged women in the United States lived in poverty. The worst socioeconomic *status* was related to younger age of menopause onset, more frequent VMS, sleep disorders, and subclinical atherosclerosis during MT.^101^

### 3.11. Low Health Literacy

According to some studies, there is a negative relation between the level of knowledge and the severity of menopausal symptoms. Women with lower schooling tend to be more symptomatic, while those with higher schooling have better health conditions and lower morbidity. Thus, women with higher educational level are more aware of the menopausal symptoms and the strategies to cope with them, adopting a healthier lifestyle, with less sexual dysfunction during menopause, which might directly influence sexual satisfaction.^100,101^

In addition, the mean age of menopause in higher-educated women is greater than that of uneducated women. Moreover, the husband’s educational level also affects the quality of life of postmenopausal women, especially regarding the psychosocial dimension, which might be attributed to better understanding and supporting their spouses.^99,101^

### 3.12. Racial Discrimination

Black women tend to enter menopause at earlier ages than White, non-Latin women, and can have a longer MT. Regarding symptoms, Black, Latin, and non-Latin women more often have VMS, sleep disorders, and depression, while Asian and non-Latin ones are more likely to report a decline in libido.^100,101^

### 3.13. Access to Health Systems

In a sample of predominantly Latin women who are homeless and/or have no health insurance, the researchers have found that the homeless ones reported more menopausal symptoms as compared to those with housing. Black women, those with lower income, and undocumented immigrants are more affected during MT. Women with no health insurance have been shown to report more bothersome menopausal symptoms than women with health insurance.^99,101^

### 3.14. Environmental Factors

Data from the European Community Respiratory Health Survey, an international population-base cohort, have shown that the age at menopausal onset is 1.4 year earlier for women living in areas with fewer green spaces as compared to women living in greener neighbourhoods.^102^

### 3.15. Social Support

Single women have more severe menopausal symptoms and earlier age at menopause onset as compared to married and divorced women, with higher risk for osteoporosis and CVD, probably because of the social relationships and family support. In addition, married women have better quality of life in menopause than single women and widows. Older age at the last pregnancy and higher number of pregnancies and deliveries delay menopausal onset, possibly because of the increased secretion of estrogen and progesterone due to uterine and ovarian activity and breastfeeding.^99,101^

### 3.16. Conclusion

The MT comprises different experiences for women, influenced by personal beliefs, cultural norms, behaviors, social environment, and traditional CVRFs. These varied factors coexist at several levels (individual, interpersonal, communitarian, and collective), resulting in unequal access to health systems. Many of such factors have not been contemplated in clinical trials, which need to include a higher number of menopausal women so that the diagnostic and therapeutic strategies can be transposed to that phase of women’s life.

## 4. Relation between Postmenopause/Menopause and Cardiovascular Diseases

### 4.1. Calculation of Cardiovascular Risk in Menopause – Peculiarities of Risk Stratifiers and Imaging Tests

One in every three women dies due to CVD worldwide,^103^ a risk that increases substantially after menopause.^104^

Women develop IHD several years after men, with a remarkable increase during MT.^105^ However, CVR stratification in postmenopausal women is an important tool to identify the major RFs and risk markers, aiming mainly at implementing therapeutic strategies and measures to prevent and reduce mortality. There is no specific risk stratification score for perimenopausal and postmenopausal women, thus, the traditional scores are used.

The major factors that influence female CVRs are race/ethnicity, reproductive history, such as former gestational diabetes and preeclampsia, CV health in premenopause, physical activity, diet, alcohol intake, smoking, and genetics, in addition to age at natural menopause, type and stages of menopause, endogenous estrogens, VMS, depression, and sleep disorders.^55^

Data from the *West Pomeranian Voivodeships*, using the scores ASCVD, SCORE2 and POL-SCORE for women at different menopausal stages,^106^ have shown that most participants were at low CVR. Age at menopause, time since menopause, and presence of MS associated with higher CVR (OR = 1.186, 1.267, and 13.812, respectively). Women who enter menopause before the age of 45 years have higher CVD and all-cause mortality, but further studies are necessary to define whether the negative CV outcomes and mortality relate to the time since menopause or to mechanisms leading to early menopause, such as genetic, reproductive (parity and menarche age), and lifestyle-related (smoking, alcoholism, and BMI) factors.

Women have risk-enhancing factors (REFs),^107^ such as autoimmune diseases (systemic lupus erythematosus and rheumatoid arthritis), which increase CVR by 2-3 times, in addition to other less common, such as systemic sclerosis, Sjögren syndrome, rheumatic polymyalgia, antiphospholipid syndrome, and giant cell arteritis. It is worth noting that breast cancer treatment with radiotherapy and CTX with anthracyclines and trastuzumabe is associated with a higher CVD risk, even years after the end of treatment.

Risk stratification can be refined with markers of subclinical atherosclerosis, such as CAC, ankle-brachial index (ABI), medio-intimal thickening (MIT), or carotid plaque on coronary computed tomography angiography (CCTA) with plaque occlusion < 50%, in the presence of doubt regarding the clinical management with lipid lowering drugs for primary prevention after the inclusion of REFs.^107^

The MESA study^108^ has shown that absence of coronary calcification (CAC = 0 in >50% of the women) associated with low/intermediate risk of atherosclerotic CVD in 10 years, being higher in early menopause; CAC = 1-99 or > 100 UA associated with higher incidence of atherosclerotic CVD, which, however, is similar in women with or without early menopause.

**Figure 4.1:**
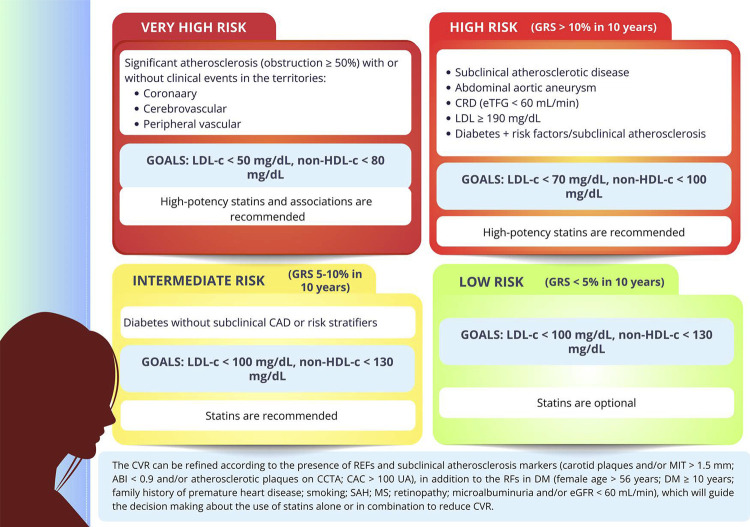
Cardiovascular risk stratification and therapeutic goals for postmenopausal and menopausal women. Adapted from Oliveira et al.13 ABI: ankle-brachial index; CAC: coronary artery calcium score; CAD: coronary artery disease; CCTA: coronary computed tomography angiography; CRD: chronic renal disease; CVR: cardiovascular risk; DM: diabetes mellitus; eGFR: estimated glomerular filtration rate; GRS: global risk score; MIT: medio-intimal thickening; MS: metabolic syndrome; RF: risk factor; REF: risk-enhancing factors; SAH: systemic arterial hypertension.

Thus, for the CVR stratification aimed at therapeutic definition for postmenopausal/menopausal women (Figure 4.1), the very-high-risk situations, such as manifest atherosclerotic CVD, and the high-risk situations (subclinical atherosclerosis, abdominal aortic aneurysm, chronic renal disease, diabetes with risk stratification, and severe hypercholesterolemia) should be initially considered. In such situations, high-potency statins, alone or in combination, are strongly recommended.

The intermediate risk and low risk stratifications are based on the global risk score (GRS). In such situations, the CVR can be refined according to the presence of REFs and subclinical atherosclerosis markers (carotid plaques and/or MIT >1.5 mm; ABI < 0.9 and/or atherosclerotic plaques on CCTA; CAC >100 UA), in addition to the RFs in DM (age > 56 years in women; DM ≥ 10 years; family history of premature heart disease; smoking; SAH; MS; retinopathy; microalbuminuria and/or estimated glomerular filtration rate (eGFR) < 60 mL/min). These markers will guide the decision making about the use of statins alone or in combination to reduce the CVR.^13^

However, studies of outcomes in primary and secondary prevention of atherosclerotic CVD remain elusive for women. Thus, recommendations specifically targeted at postmenopausal and menopausal women need to be elaborated.^105^

### 4.2. Acute and Chronic Ischemic Heart Disease

**Figure 4.2:**
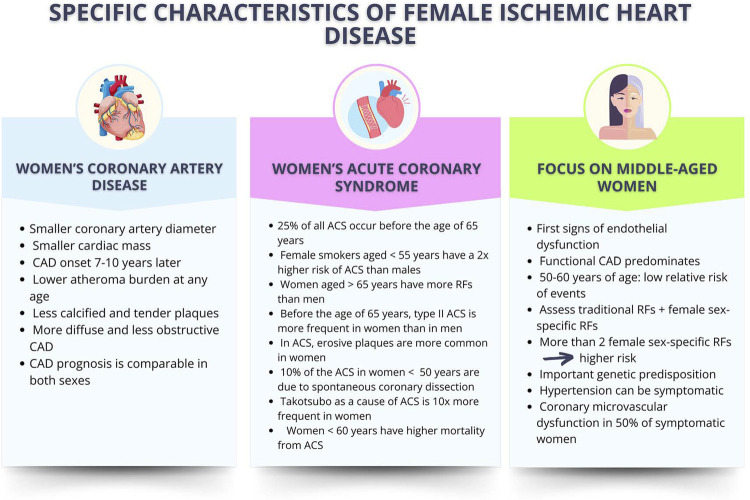
Specific characteristics of female ischemic heart disease as compared to the male one. Adapted from Elias-Smale et al.121 ACS: acute coronary syndrome; CAD: coronary artery disease; RF: risk factor.

Recent studies have shown great advances on the knowledge of women’s IHD, which have specific characteristics regarding symptoms and pathophysiology, with positive impact on mortality rates (Figure 4.2). However, deeper investigation of that pathology in postmenopause and menopause is required.

The risk of a CV event in younger women in premenopause is lower; however, this tendency reverses with aging. Premenopausal women have a relatively lower risk of IHD as compared to men of the same age group, but this gender difference decreases after menopause.^109^

#### 4.2.1. Coronary Anatomo-functional Changes

The total atherosclerosis burden is lower in women, with a more diffuse and less obstructive pattern of CAD. However, more than 50% of symptomatic middle-aged women have coronary microvascular dysfunction.^109^

In postmenopause, the plaques characteristically show less calcification, which is associated with estrogen’s protective properties against arterial aging. However, there is a gradual increase in more vulnerable and calcified plaques after the age of 60 years.^22^

In the WISE study, an analysis of postmenopausal women with suspected IHD has revealed higher prevalence of angiographic IHD and worse CV event survival of those with history of menstrual irregularity and biochemical evidence of hyperandrogenemia. Similarly, the *Rancho Bernardo Study* has concluded that IHD was associated with PCOS (history of menstrual irregularity, hyperandrogenism, infertility, central obesity, and insulin resistance) in a large cohort of postmenopausal Caucasian women.^110^

#### 4.2.2. Timing of Menopause and Development of Chronic Ischemic Heart Disease

Studies have shown that natural early menopause is associated with CVD, possibly due to the estrogen’s vasoprotective effects in premenopausal women. Kalarantidou *et al*. have shown that women with POF have abnormal endothelial function, assessed via brachial artery flow-mediated dilation, and that condition can be changed with cyclic hormone therapy of estrogen/progestogen.^111^

In recent years, several studies have investigated the relation between age at natural menopause and the risk of IHD and reported an increase in mortality from IHD in women with early menopause, despite speculations regarding the increased risk in women with very late menopause.^112^

A study involving 302 632 Chinese women has revealed that age at menopause and the total of reproductive life years were inversely associated with fatal and nonfatal CVD, especially CAD, with increasing risk over time since menopause.^113^

#### 4.2.3. Acute Ischemic Heart Disease

In acute coronary syndrome (ACS), younger women have a two-time lower likelihood of having significant coronary lesions as compared to men.^114^However, in men and women after MT, the classic pattern of plaque rupture followed by thrombus formation is commonly observed. In addition, in younger women, ACS often manifests with plaque erosion and type II ACS (functional coronary disease).^115^

Spontaneous coronary dissection is more prevalent in young women, representing 10% of all ACS under the age of 50 years. It can occur in women without apparent RFs, being associated with a combination of tissue disease or fibromuscular dysplasia. In addition, it is related to pre-SAH during pregnancy or after delivery, and often triggered by stressful situations.^116^

Regarding the clinical presentation of ACS, nonspecific symptoms of chest pain and dyspnea are common in middle-aged women. Vascular endothelial dysfunction emerges as the first manifestation of arterial aging, characterized by imbalance between vasodilation and vasoconstriction, which can result in the first manifestations of chest pain and dyspnea.^117^ The WISE study has shown that, in more than 50% of middle-aged women, the symptoms of chest pain were related to vascular dysfunctions in epicardial coronary arteries and microcirculation rather than to obstructive CAD.^118-120^

## 4.3. Cerebrovascular Disease

Of the etiologies of cerebrovascular disease, stroke is the most prevalent, affecting 94 in every 100 000 individuals per year worldwide.^122,123^ In addition, after a transient ischemic attack or minor ischemic stroke, 6.2% of the patients are affected by a new stroke within one year, and the risk of recurrence increases to an estimated cumulative rate of 12.9% over 5 years.^124^

Cerebrovascular disease is an important cause of morbidity and mortality worldwide and has particularities in women,^122^ representing the second cause of death and third cause of disability. Women have higher incidence of stroke than men at more advanced ages, which can be partially explained by their longer lives.^122^ Significant disparities per race/ethnicity in that age range have been reported, and Black and Hispanic women aged ≥70 years have a 76-77% higher risk of stroke as compared to White women after adjusting for age.^122^ In Brazil, cerebrovascular diseases are also one of the major causes of death and disability. Stroke is more prevalent in low socioeconomic individuals and can be partially explained by insufficient access to health services and poorer control of RFs, such as SAH, DM2, and smoking. Although mortality from stroke in Brazil tends to decline, which can be attributed to improvement in prevention, diagnosis, and treatment, there are still significant regional inequalities, with higher rates in the Northern and Northeastern regions of Brazil.

Stroke outcomes tend to be significantly more severe in women, with higher mortality rates and worse functional recovery.^125^

### 4.3.1. Common Risk Factors for Stroke

**Figure 4.3:**
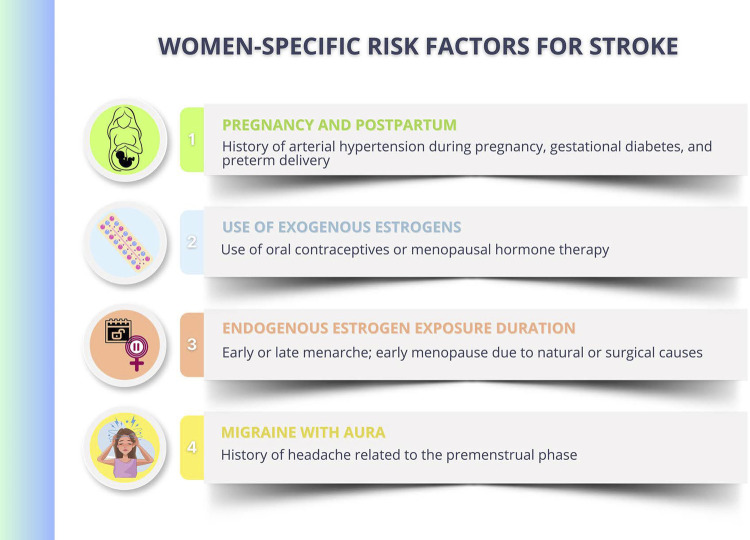
Women-specific risk factors for stroke.

The INTERSTROKE study has suggested that ten common RFs accounted for approximately 90% of the population attributable risk for stroke worldwide.^126^ The most prevalent RF is SAH, and recent studies have shown a higher negative impact on women. Diabetes mellitus type 2 is an important RF for ischemic and hemorrhagic stroke, with higher risk in women.^122^ Recent data have found no difference between men and women regarding the impact of DLP on the stroke prevalence, and data on the increment of ischemic and hemorrhagic stroke in the female population in the presence of obesity are controversial. Smoking has a direct relation with stroke prevalence, and impact is higher on women.^127^ Some RFs are exclusive to women (Figure 4.3).

## 4.4. Heart Failure

Menopausal physiological changes influence several organs and systems, and the CV system is one of the most affected.^55^ As compared to premenopausal women, postmenopausal women more frequently have: LV systolic and diastolic dysfunction, higher LV relative wall thickness and LV concentric remodeling, and ventricular relaxation changes.^128^ A cohort study with more than 1.4 million postmenopausal women has shown a 33%-higher risk of HF in menopause, after adjusting for CVRFs, and that earlier age at menopause gradually increased the HF incidence.^129^

**Figure 4.4:**
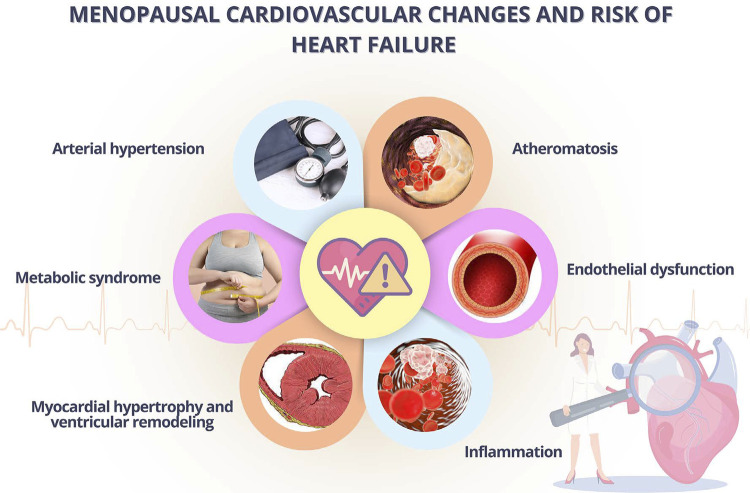
Menopausal cardiovascular changes and risk of heart failure. Adapted from Muka et al.132

Postmenopausal CV changes contribute with multiple factors to the risk of developing HF.^130^ Estrogen deficiency predisposes to higher risk because of its direct or indirect effect on diastolic dysfunction, this being one of the major causes of HF in women. As the estrogen levels decline, menopausal women are more likely to have cardiometabolic RFs.^131^ Estrogen loss in postmenopause can activate the RAAS, which activates intracellular signaling pathways, resulting in endothelial dysfunction, inflammation, vascular injury, LV remodeling, and eventual diastolic dysfunction, leading to HF.^132^(Figure 4.4)

The longer the period of estrogen deprivation in early menopause, the higher the cumulative risk of HF, mainly in the presence of previous CVRFs, as shown in some studies.^133,134^

Because of the HF burden in women and its increased prevalence, further research is required to establish the causality and to understand the underlying mechanisms of the early onset of menopause and how it can contribute to HF. This is relevant information to implement interventions aimed at improving CV health of postmenopausal women.

## 4.5. Venous Thromboembolism

Venous thromboembolism, including deep venous thrombosis (DVT) and pulmonary embolism (PE), has an incidence of 1 per 1000 women-year in postmenopause. Approximately 10% of the cases can be fatal, and PE is the major cause of death.^135^

Menopause leads to changes in the CV system that can contribute to increase CVR, but there is no direct association of menopause with higher risk for VTE. However, the risk for VTE increases exponentially with age and can be associated with the higher prevalence of RFs for VTE, such as obesity, cancer, hospitalization, or other comorbidities of elderly women.^136^

It is worth noting that women at increased risk for VTE, those older than 60 years and/or with more than 10 years from menopause onset should avoid MHT because of the enhanced risk for thromboembolic events.^136-138^

## 4.6. Arrhythmias

Based on recent observational data, reproductive factors (menarche, POF and early menopause, recurrent gestational losses, time and number of gestations) associate with the risk of CVD in women, and menopause is the strongest marker of CVR. When premature, that is, before the age of 40 years, menopause increases the risk of myocardial infarction (MI), stroke, HF, and CV mortality. Regarding arrhythmias, however, there is little data in the literature correlating arrhythmia and menopause. Atrial fibrillation (AF) is one of the most common diseases of aging and is associated with multiple factors, such as CV events, inflammation, higher frequency of thrombosis, hormonal dysregulation, suggesting a correlation between menopause and increased risk for AF.^139^

### 4.6.1. Atrial Fibrillation and Menopause

**Figure 4.5:**
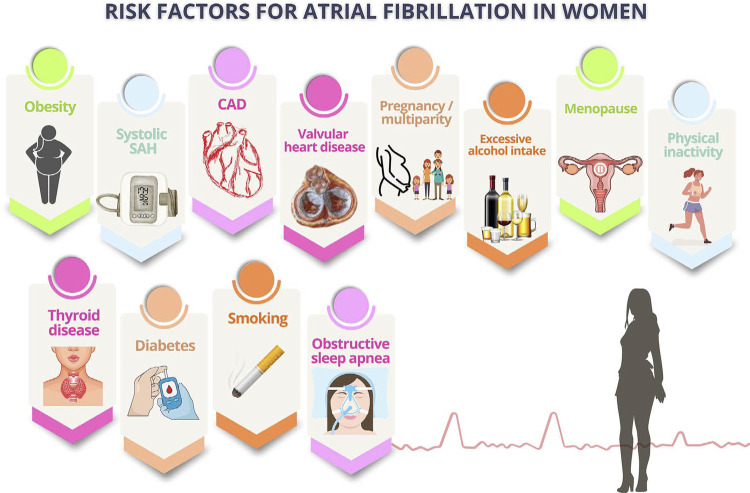
Risk factors for atrial fibrillation in women. MHT: menopausal hormone therapy; SAH: systemic arterial hypertension.

It is estimated that 29.4 million women have AF worldwide. Although the incidence is higher among men, elderly women have more AF because they have a longer life expectancy.^140^Women have specific RFs for AF, such as systolic SAH, obesity, sedentary lifestyle, excessive alcohol intake, valvular heart disease, multiparity, and CAD (Figure 4.5).^13^

Currently, the increase in the risk for AF in women in menopause or on MHT has been questioned.^141^A study with 30 034 healthy women with no hysterectomy and/or oophorectomy prior to menopause, which had occurred close to the age of 50 years, in a 20.5-year follow-up, has prospectively assessed the relation between menopausal age, MHT, and AF incidence. The authors reported 1350 AF events, but the menopausal age did not add risk to that inherent to age itself.^142^

However, a study performed in 1 401 175 postmenopausal women has assessed the association between early menopause, menopausal age, and risk of AF. At a 9.1-year follow-up, there were 44 834 (3.2%) new cases of AF, and history of early menopause associated with an increased risk of AF. There was an increased incidence of AF when age at menopause was under 50 years as compared to over 50 years, mainly when age at menopause was under 40 years (POF). The results show that, the sooner menopause occurs, the higher the risk for AF, indicating the need for prevention and care in that specific group of women.^129^Recent meta-analysis with 9 studies, including 6 255 783 postmenopausal women, has evidenced that those with early menopause (before the age of 45 years) or premature menopause (before the age of 40 years) had increased risk of AF as compared to those with menopause at usual age. However, the exact mechanism has not been elucidated, requiring future prospective studies.^143^ A study with 16 729 women followed up for 8.5 years has shown that 3943 developed AF, which was associated with C-reactive protein and interleukin (IL) levels, but not with IL-1β on multivariate analysis.^144^

In menopause, important behavioral changes occur, in which stress, anxiety, insomnia, and depressive symptoms can activate inflammatory and neuro-hormonal factors that potentialize the development of AF. Inflammation plays an important role in that arrhythmia. A recent study on the correlation of cytokines and AF incidence in postmenopause has assessed 83 736 women (mean age of 63.9±7.0 years, 10.5±6.2-year follow-up) and 23 954 cases of AF were observed. In postmenopausal women, insomnia and stressful life events were the major psychosocial factors associated with arrhythmia.^145^

Thus, menopause-related factors, such as onset time of menopause, presence of associated RFs, and behavioral factors, such as stress, anxiety, quality of sleep, and depression, should be considered in the implementation of measures to reduce CVR, mainly HF and AF.^143^

## 5. Menopause and Risk of Morbidity and Mortality from Other Diseases

### 5.1. Cancer

The intersection of CVD, cancer, and menopause represents an area of increasing interest in medicine. Cancer and CVD are the major causes of death worldwide, and they share some RFs, such as age, obesity, smoking, family history, and diet.^13^

For both women and men, CVD is the major cause of mortality, but there is a remarkable increase after menopause.^55^

Both obesity and MS are associated with an increased incidence of DM2, CVD, breast cancer (postmenopausal), and other cancers.^146^

The CVR in postmenopausal women treated for breast cancer is higher than that in women without breast cancer. Postmenopausal breast cancer survivors have shown a strong association with MS, DM, atherosclerotic disease, hypertriglyceridemia, SAH, and abdominal obesity, which are major CVRFs, as compared to postmenopausal women without breast cancer. In postmenopausal women with breast cancer at the initial stage, the risk increases sharply so that the mortality rates from CVD in 10 years are similar to the mortality rates from cancer itself.^147^

The increase in CVR in menopausal women with cancer is due not only to the inadequate control of CVRFs, but also to the cancer treatment because of its secondary cardiotoxic effects, such as ventricular dysfunction, SAH, arrhythmias, myocardial ischemia, valvular disorders, thromboembolic disease, pulmonary hypertension, and pericarditis, in addition to atheromatosis.

Chemotherapy with anthracyclines and trastuzumabe can cause cardiac dysfunction in the short, medium, and long run. Radiotherapy to the left hemithorax can lead to secondary CV effects, such as coronary atherosclerosis, that can emerge more than 5 years after exposure, and the risk persists for up to 30 years. Hormone therapy with aromatase inhibitors increases the risk for atherosclerotic disease.^148^

Late CV effects of cancer develop over several decades, which for many women may overlap with reproductive and lifecycle events. Thus, women need longitudinal cardio-oncologic care that anticipates CVRs and responds to their evolution.^149^

Women with cancer may have early, gradual, or rapid menopause, depending on the baseline ovarian reserve, gonadotoxicity, and duration of exposure to oncogenic agents (oncological and/or endocrine therapy).^150^

Female childhood cancer survivors are at risk of developing early menopause due to POF after oncological treatment.^151^

The CVR is higher in early menopause because of prolonged endogenous estrogen deprivation, leading to a variety of metabolic and vascular effects, including glucose intolerance, DLP, SAH, and endothelial dysfunction.^152^

The POF not only confers risk of IHD after adjusting for conventional RFs, but also predicts worse ischemic outcomes and higher mortality.^153^

Menopause induced by oncological treatment can be caused by surgical bilateral oophorectomy, CTX, and radiotherapy to the pelvis and/or hormone suppression therapy. Bilateral oophorectomy causes acute and permanent menopause, and, when before the age of 50 years, it increases the risk of global CVD (relative risk [RR]: 4.55; 95% CI, 2.56-8.01), HF, and stroke.^154^

Chemotherapy and radiotherapy to the ovaries can lead to ovarian dysfunction and consequent secondary menopause, which can be temporary or permanent, depending on patient’s age, type and dose of the drug, treatment duration, and, in case of radiotherapy, its site and dose used.^155^

Some hormone suppression therapies or endocrine therapies, with aromatase inhibitors or selective estrogen receptor modulators, can temporarily prevent ovulation and cause temporary menopause. Treatment with tamoxifen and aromatase inhibitors for 5 years increase the 20-year survival rate up to 85%, with 22% risk of recurrence.^156^

Endocrine therapy is a common treatment, because 65–70% of all patients with early and metastatic breast cancer develop hormone receptor-positive disease. Endocrine therapy involves the reduction of levels or inhibition of biological activity, stopping/delaying or preventing cancer growth. Selective estrogen receptor modulators (tamoxifen, toremifene) or aromatase inhibitors (letrozole, anastrozole, or exemestane) are recommended for early breast cancer according to the menopausal *status*, comorbidities, and risk of disease relapse.^155^

Tamoxifen is the endocrine therapy of choice for premenopausal women, while the strategies for postmenopausal women can include tamoxifen, aromatase inhibitors, or a sequential combination, with careful assessment of the benefits and management of the toxicity risks.^148^

The use of aromatase inhibitors increases the risk of DLP, MS, SAH, HF, and MI.^156^ In the ATAC study (anastrozole and tamoxifen alone or in combination), patients with preexisting IHD treated with anastrozole had more CV events (17% vs. 10%) and elevation of cholesterol levels (9% vs. 5%) than those treated with tamoxifen.^157^ The significant increase in the risk of thromboembolic disease was consistently shown with tamoxifen, which, thus, is not recommended for patients at increased risk for thrombosis. The risks for thromboembolic disease, hypercholesterolemia, and CVD should be discussed with the patients, although it is recognized that the absolute benefits of preventing breast cancer recurrence usually outweigh the CVRs.^158^

In conclusion, there is association between cancer and increase in CVRs in postmenopausal women, as well as different psychosocial and physical barriers to access CV care.

Thus, menopausal women with a history of cancer should be monitored by specialized professionals to have their CVR assessed, undergo complementary tests, and receive preventive measures, and, if necessary, medications to reduce their CV morbidity and mortality.

### 5.2. Dementia

The consequence of world population aging is the increase in the prevalence and incidence of chronic and neurodegenerative diseases.

Currently, 50 million people worldwide are estimated to have some form of dementia, and 10 million new cases are diagnosed per year worldwide. In Brazil, around 1.7 million elderly are estimated to have dementia, with a prevalence of approximately 1036/100 000 inhabitants.^159^ The global estimates of dementia prevalence are of up to 7% of individuals over the age of 65 years, with future estimates indicating higher prevalence in low- and middle-income countries.^160,161^

**Figure 5.1:**
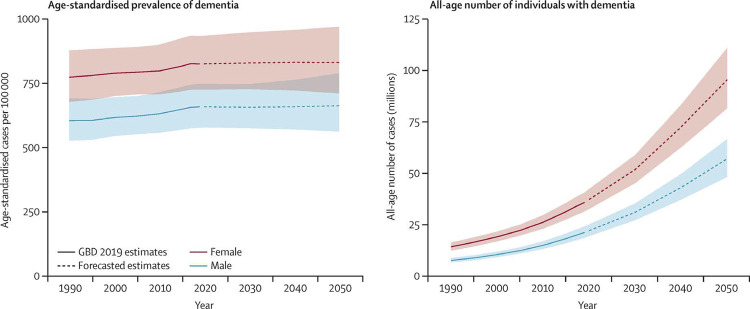
Estimated trends in the global age-standardized prevalence of dementia (A) and the all-age number of cases of dementia (B). Source: GBD 2019.162

Data from the *Global Burden of Disease* (GBD) study have shown that, in 2019, there were more women with dementia than men (1.69 woman:1 man ratio), with a forecasted prevalence of 1.67 woman: 1 man ratio in 2050^162^ (Figure 5.1). However, there is higher prevalence of vascular risks in men as compared to women, suggesting potentially strong mechanisms of neutralization that boosts those inequalities. Although the difference between sexes could be partially explained by the higher life expectancy of women, there is former evidence of potential differences between the sexes also in the underlying biological mechanisms.

**Figure 5.2:**
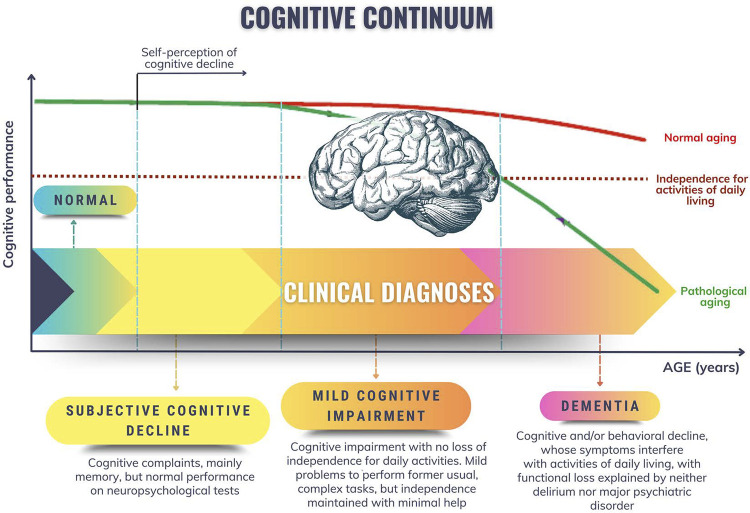
Decline of cognitive continuum in normal and pathological aging.159

Cognitive decline occurs in all individuals as age advances and can vary from mild impairment, without loss of autonomy or subjective decline, in which neuropsychological tests are normal, to dementia. This process occurs continuously with normal aging and in pathological situations, in which there is inability to perform daily tasks (Figure 5.2).

Of the degenerative dementias commonly found in the elderly,^160^ Alzheimer’s disease is the most prevalent form, corresponding to 70-80% of the cases. Vascular dementia corresponds to 15%, being associated with CVRFs. Other causes are Lewy body dementia, frontotemporal lobe degeneration, and Parkinson’s disease, the last one corresponding to 10% of the cases. Vitamin deficiencies (B12 and thiamine), hypothyroidism, normal pressure hydrocephalus, chronic alcohol abuse, CTX-related cognitive dysfunction, intracranial masses (subdural hematomas, brain tumors), traumatic brain injury, and psychiatric disease (deep depression/anxiety)^160^ are common causes of non-neurodegenerative mild cognitive impairment and dementia that can occur throughout life. Of the mixed-cause dementias, Alzheimer’s disease associated with vascular dementia is the most common.^160^

Aging, genetic profile (APOE ε4 allele), and systemic vascular disease are the major nonmodifiable RFs for the development of dementia, and ethnicity and gender are also worth noting.^162^ Although dementia is not part of normal aging, age is its major known RF, and its incidence increases proportionally to population aging. Nevertheless, in some countries, the incidence of dementia according to age has declined probably due to improvements in education, nutrition, health care, and lifestyle changes. Regarding gender, the prevalence of dementia is higher among women, not only because most elderly are women, but also because women are more impacted by the modifiable RFs that affect cognitive reserve. Regarding ethnicity, several RFs are grouped around inequalities, which occur mainly in Blacks, Asians, and minorities, as well as in vulnerable populations. The early onset of Alzheimer’s disease associates with genetic factors, but the mostly known gene to be associated with the later appearance of Alzheimer’s disease is the APOE ε4 allele.^162^

Of the modifiable RFs, the following stand out: less education, SAH, hearing impairment, smoking, obesity, depression, sedentary lifestyle, DM, social isolation, alcoholism, traumatic brain injury, and air pollution. Around 40% of dementias worldwide can be prevented or delayed with intervention on those RFs. The presence of early-life RFs, those appearing before the age of 45 years, such as less education, affects cognitive reserve. The midlife (45-65 years) and later-life (older than 65 years) RFs influence that reserve and triggering of neuropathological developments.^162^

#### 5.2.1. Female Reproductive Aging and Cognitive Decline

Menopausal transition is a process of midlife neuroendocrine aging that culminates in reproductive senescence and occurs in stages characterized by unique endocrine properties that impact the aging trajectories of multiple organic systems, the brain included. Thus, MT is considered a state of reproductive and neurologic transition, such as evidenced by the fact that many menopausal symptoms are neurological, such as VMS, sleep disorders, mood swings, and forgetfulness.^163^

Gonadal steroid hormones, especially 17β-estradiol, are well-known regulators of reproductive and neural function, and, during MT, their levels decrease substantially in the body and brain.

Menopausal transition has marked effects on brain structure, connectivity, energy metabolism, and amyloid β protein (Aβ) deposition. A multimodality neuroimaging study has been conducted in women across different MT stages (pre-, peri-, and postmenopause) to investigate its effects on the structures of the brain gray and white matters.^163^ The results indicate that MT significantly impacts brain biomarkers in regions involved in higher-order cognitive functions. The effects, independently of age and use of hormonal therapy, were specific of menopausal endocrine aging and not chronological aging, as determined by the comparison with age-matched men. It is worth noting that cognition was preserved in postmenopause, which correlated with the recovery of the gray matter volume and the cerebral adenosine triphosphate production, suggesting potential compensatory mechanisms. Finally, Aβ deposition was higher among peri- and postmenopausal women carrying the APOE-4 genotype, indicating specific effects of that gene on the risk of Alzheimer’s disease with perimenopausal onset.^163^

#### 5.2.2. Conclusion

Because of the absence of recent and effective treatments to change the progression of dementia, immediate efforts to reduce its prevalence in the future should be directed to prevention, through interventions on modifiable RFs. Interventions that change the prevalence of such RFs can reduce in up to 40% the expected prevalence of dementia in coming years, according to the results of the Lancet Commission 2020 update on dementia prevention, intervention, and care.

In conclusion, significant changes in exposure to RFs have been suggested to considerably change forecasted estimates and reduce the future burden of dementia worldwide.^162^

## 5.3. Thyroid Dysfunction

Thyroid disorders are significantly more common in women, and their incidence increases with aging, given that the physiological production of thyroid hormones decreases as age increases.^164^

One in every eight women is likely to have some type of thyroid dysfunction throughout life,^165^ mainly during the peri- and postmenopause.^166^

Few studies on the relation between menopause and thyroid function have been conducted; thus, if menopause influences thyroid independently of aging could not be clarified.^166^

However, thyroid activity is age-dependent,^167^ because, with aging, there is a reduction in iodide uptake by the gland, in free T4, free T3 synthesis, and free T4 catabolism. Although reverse T3 increases, the TSH level remains normal, sometimes tending to higher limits.^168^

Considering the well-known evidence about the effect of the thyroid *status* on cognitive function, CVRs, bone remodeling, and longevity, it is not difficult to understand the risk for thyroid dysfunctions in menopausal women.^165,166^

**Figure 5.3:**
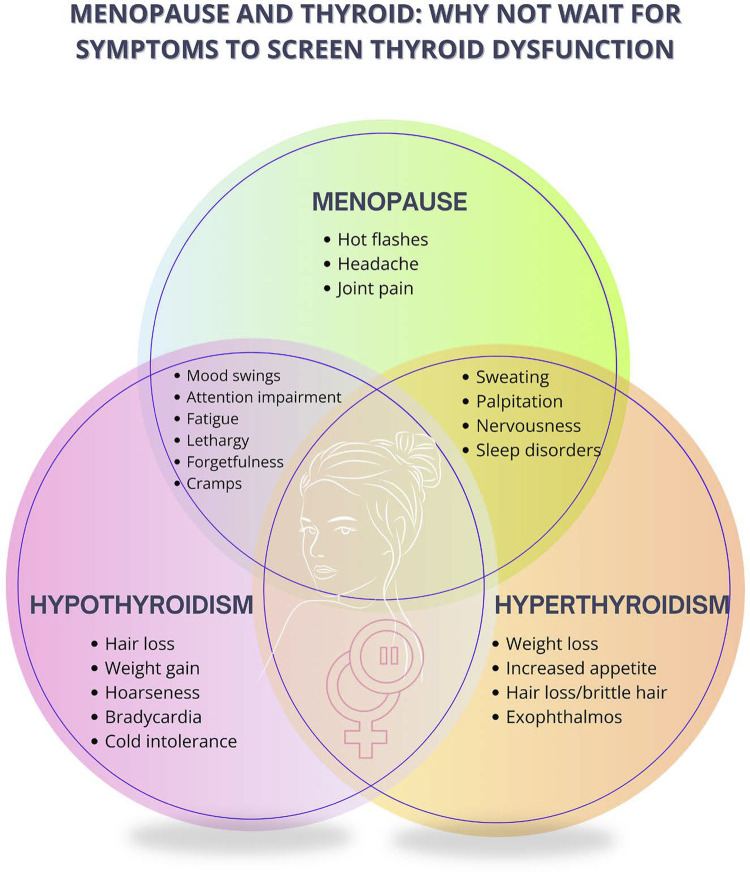
Overlapping symptoms of thyroid dysfunctions and menopause.

Thus, menopausal women should undergo routine screening for thyroid disorders, especially because symptoms of thyroid disease and postmenopause often overlap, which can delay the diagnosis of thyroid dysfunction (Figure 5.3).^167^

### 5.3.1. Hypothyroidism

The occurrence of hypothyroidism, goiter, and thyroid nodules increases with age, and hypothyroidism is more prevalent in the elderly, 2-20% of whom have some type of hypothyroidism.^165^

The prevalence of subclinical hypothyroidism (HypoSC) in the population is approximately 4-10%, higher among women and the elderly, and inversely proportional to the iodine content in the diet.^169^ The potential risks of HypoSC in the elderly include progression to manifest hypothyroidism, CV effects, DLP, and neurological and neuropsychiatric effects.

Fatigue, lethargy, forgetfulness, attention or concentration impairment, and mood disorders are characteristics common to both hypothyroidism and menopause, which makes hypothyroidism pass unnoticed sometimes, with symptoms being exclusively attributed to menopause, side effects of drugs, or aging itself.^166^

Of the causes of hypothyroidism, iodine deficiency and autoimmune disease stand out. However, iodine therapy, radiotherapy for head and neck malignancy, and central hypothyroidism due to hypophysis or hypothalamus tumors should be mentioned as a cause in women, mainly after menopause.^165,170^

Usually, HypoSC is asymptomatic in that population and not associated with cognitive function effects, depression, or anxiety. There is no consistent evidence about the consequences in cardiac structure and systolic and diastolic functions in population studies.^169^ There is consistent evidence of the association of HypoSC with the risk of IHD, especially for TSH levels ≥ 10 mU/L, although these data have not been observed in patients older than 65 years.^169^It is uncertain if middle-aged women with HypoSC should be treated.^13^The Latin-American Society Guidelines on Thyroid recommend no routine treatment for the elderly with HypoSC if TSH levels < 10 mU/L. In addition, they do not recommend the treatment of HypoSC to improve the cognitive function in the elderly, but the treatment can be considered on an individual basis.^170^

Because of lack of data from robust studies showing benefits regarding CVR and mortality risk, the treatment of HypoSC remains controversial. It can be considered for persistent HypoSC after the confirmation of serum levels of TSH ≥ 10 mU/L after 3 to 6 months, because of the higher risk of progression to manifest hypothyroidism, HF, CAD, and mortality.^169^Cohort studies have shown indirect evidence of benefits from the treatment of HypoSC regarding CVR and mortality, in addition to a favorable effect on TC in patients with HypoSC and TSH > 10 mU/L.^169,171,172^

Use of medications and liver and kidney diseases may affect the metabolism of thyroid hormones or change the proteins binding to these hormones. Thus, serum TSH should be monitored with the use or suspension of oral estrogens and androgens, because these medications can change the need for levothyroxine, increasing thyroid-binding globulin, which reduces free T4.^164,166^

Therapy with levothyroxine can induce a relevant improvement in some CV parameters, such as increased cardiac output, decrease in systemic vascular resistance and in end-diastolic volume, effects more evident in clinical than in subclinical disease. However, it can increase oxygen consumption, thus inducing myocardial ischemia in patients with underlying CAD.^170,172^ Recent evidence has suggested an increase in the risk of fracture in patients > 70 years of age at usual doses. Thus, for elderly patients and those with IHD and HF, the guideline recommends beginning levothyroxine at the dose of 12.5-25 µg/day, especially for patients with HypoSC.^170^

### 5.3.2. Hyperthyroidism

Hyperthyroidism occurs at the proportion of 5 women:1 man. Its prevalence (approximately 1.3%) increases to 4–5% in elderly women, among whom toxic nodular goiter is the most common cause. In addition, hyperthyroidism induced by drugs, such as contrast medium and amiodarone, should be mentioned as a cause.^166^

Hyperthyroidism in elderly patients can be lethargic rather than have the classic presentation of sympathetic system hyperactivity, such as tremors and palpitations.^166^ In cross-sectional studies, elderly patients had a reduced risk for classic symptoms (heat intolerance, tremor, nervousness) and higher prevalence of weight loss and shortness of breath as compared to younger patients, in addition to a higher rate of moderate to severe ophthalmopathy and AF. Tachycardia, however, can be absent because of concomitant conduction system disease.^166^

The typical symptoms of hyperthyroidism mimic the menopause-related symptoms, but, as age advances, the clinical symptoms decrease.

Slopien *et al*.^167^have reported that the serum concentration of TSH has a negative correlation with symptoms, such as sweating, palpitations, and weakness, while that of free T4 has positive correlation with palpitations, paresthesia, and nervousness.

**Figure 5.4:**
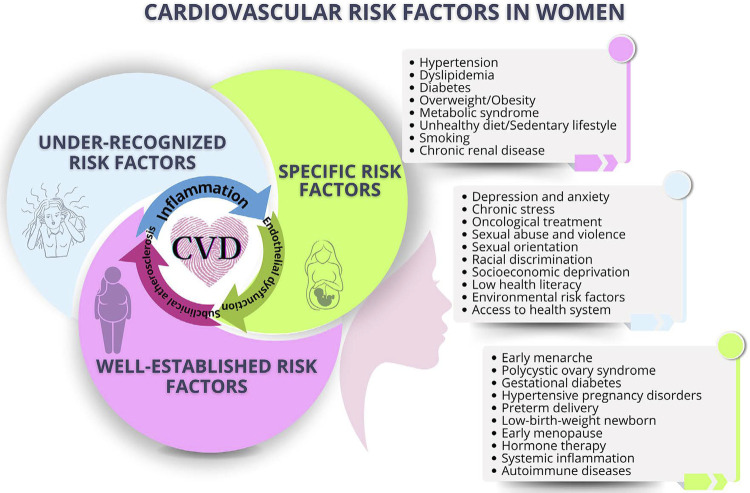
Menopause and hyperthyroidism: accumulation of risks. AF: atrial fibrillation; CAD: coronary artery disease.

In hyperthyroidism, the cycles of bone remodeling are reduced, resulting in a high bone turnover rate. With bone resorption exceeding mineralization, there is bone mass loss of approximately 10% per cycle. In addition, there is a reduction in calcium absorption in the intestine and an increase in calcium renal excretion, resulting in a negative balance of that electrolyte. There is evidence that both manifest hyperthyroidism and subclinical hyperthyroidism (HyperSC) increase the risk of osteoporosis, especially in postmenopause (Figure 5.4).^164^

Although the treatment of manifest hyperthyroidism is always recommended, the management of HyperSC is less clear. Several studies have shown similar deleterious effects in both HyperSC and manifest hyperthyroidism. A recent meta-analysis has found increased risk of CAD in HyperSC (HR= 1.44 [1.06; 1.94]).^164^ The effects of long-term TSH suppression include increased heart rate at rest, frequent arrhythmias (especially AF), and reduced cardiac function. This higher CVR also extends to the TSH suppression in patients on treatment with levothyroxine.^164^

Studies have shown an increase in CV mortality in HyperSC.^166^ Recent data indicate that patients with HyperSC are at risk for developing HF, especially the elderly and those with lower levels of TSH^166^(Figure 5.4).

A population study has shown that HyperSC increased the risk of stroke in individuals older than 50 years (HR = 3.39), although a meta-analysis has been inconclusive.^166^

### 5.3.3. Thyroid Autoimmunity and Premature Ovarian Failure

Autoimmune etiology accounts for approximately 5% of the cases of POF, and autoimmune thyroid disease is present in 14–27% of women at the first diagnosis. Thus, measuring TSH levels and the presence of anti-thyroid peroxidase antibodies (anti-TPO) in those patients is recommended.^166^

## 5.4. Depression and Anxiety

**Figure 5.5:**
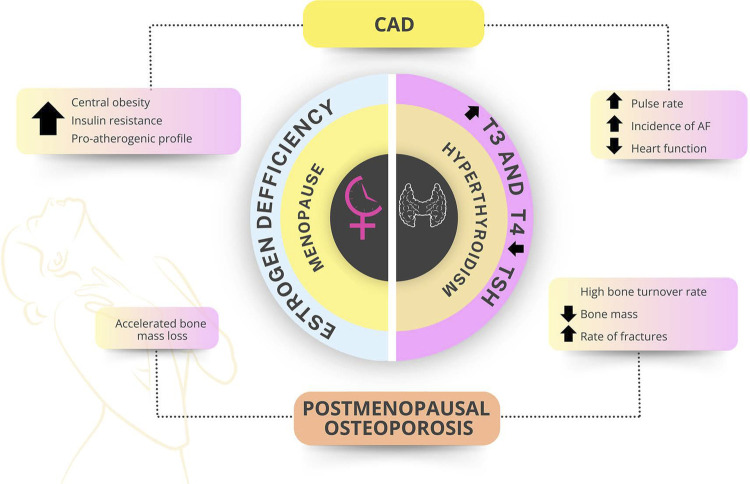
Correlation between specific, under-recognized, and well-established risk factors of cardiovascular disease (CVD) in women.173

Studies have shown the importance of classic RFs in CVD. In women, however, the gender-specific RFs, as well as the under-recognized RFs, such as depression and anxiety, also have a significant impact on CVR^13^ (Figure 5.5).

One out of five adults has some type of psychiatric disorder throughout life. In women, however, gender intrinsic and extrinsic factors can compound the occurrence of those disorders, which are directly related to the increase of CVR in that population.^173,174^

Recent studies have correlated mental diseases, such as generalized anxiety disorder and depression, with the increase in CVR, because of the metabolic changes triggered by the daily stress and injuries from those comorbidities.

Different patterns of changes in sex hormones and body fat distribution of lipids and lipoproteins across menopause relate to the potential to accelerate the CVD development in that phase of a woman’s life.^55^

Women with generalized anxiety disorder or depression can develop some degree of social impairment because of the illness mechanism. This impairment can be naturally intensified in certain phases of life, such as menopause, and this correlation has been studied more and more frequently. It is important to note that postmenopausal symptoms, such as VMS, night sweating, sleep and libido disorders, and cognitive changes, can intensify mental comorbidity findings and mask their diagnosis, delaying treatment.^173^

Regarding perimenopause, triggers, such as changes in career and relationships, awareness about aging issues, changes in physical appearance, personal and family disease, and the empty nest syndrome, can compound some feelings such as sadness, fear, frustration, and feelings of not being good enough or of inadequacy, surfaced by the depressive/anxiety syndromes. The pathophysiological mechanism involves the autonomic nervous system and the hypothalamus-hypophysis-adrenal axis, leading to an increase in cortisol and catecholamines and homeostasis changes.^173,174^In addition, the presence of psychiatric disorders associates with unhealthy behaviors that contribute to increase the CVR, such as binge eating, smoking, alcoholism, sedentary lifestyle, and lower adhesion to the treatment of comorbidities.^175^

The harmful role of the generalized anxiety disorder and depression in CV health has been shown in recent studies, evidencing that anxious people are at a twice higher risk of cardiac death as compared to the general population.^176^In addition, a potential cardioprotective effect has been attributed to emotional support.^175^

Thus, the longitudinal follow-up of a woman’s health by a multiprofessional team is of fundamental importance to differentiate a healthy phase of menopause from that associated with depression or anxiety. Early intervention with strategies of proper prevention, diagnosis, and treatment are aimed at reducing the CVR of menopausal women.^174,176^

## 5.5. Osteoporosis and Menopause

Impairment of CV and bone health is directly related to the age at menopausal onset. There is a strong correlation of POF and early menopause with increased CVR, osteoporosis, fractures, and mortality in women.^177^ The pathophysiological mechanism is related to the direct effect of estrogen on endothelial function and on bone metabolism, because the impact of traditional RFs, such as SAH, DLP, obesity, and hyperglycemia, which are frequent in women who enter menopause at the usual age, is smaller in those conditions.^177^

The systemic effects of estrogen deprivation increase the risk of depression, dementia, and osteoporosis, and, thus, of all-cause mortality. The POF and early menopause can be related to genetic or environmental factors, and, because of the increased risk of adverse outcomes to women’s health, should be considered a marker of risk.^132^

Estrogen plays an important role in bone homeostasis, through its receptors located in the precursors of osteoblasts and osteoclasts, thus maintaining the balance between bone resorption and formation. Estrogen reduces resorption and the bone remodeling velocity through the reduction in the number of osteoclasts and their useful life, via apoptosis of osteoclasts, thus maintaining bone mass. In addition, it seems to attenuate the apoptosis of osteoblasts and osteocytes.^178^

Menopausal estrogen loss leads to negative bone remodeling and consequent bone loss, as evidenced in studies showing increased formation of osteoclasts and bone resorption, partially as a result from increased apoptosis of the osteocytes.^178^ Thus, the earlier the menopause onset, the higher the risk of osteoporosis, fractures, and increased all-cause mortality.^177^

In menopausal women, several interventions have been proposed to optimize women’s bone and CV health. Changes in lifestyle, such as regular physical activity, balanced diet, diet supplementation, and weight control are fundamental. The MHT should be indicated for women with POF, but there is little evidence regarding its use in women with early menopause. For those entering menopause at the usual age, MHT can be indicated to prevent osteoporosis, especially in the presence of VMS.^179^

**Figure 5.6:**
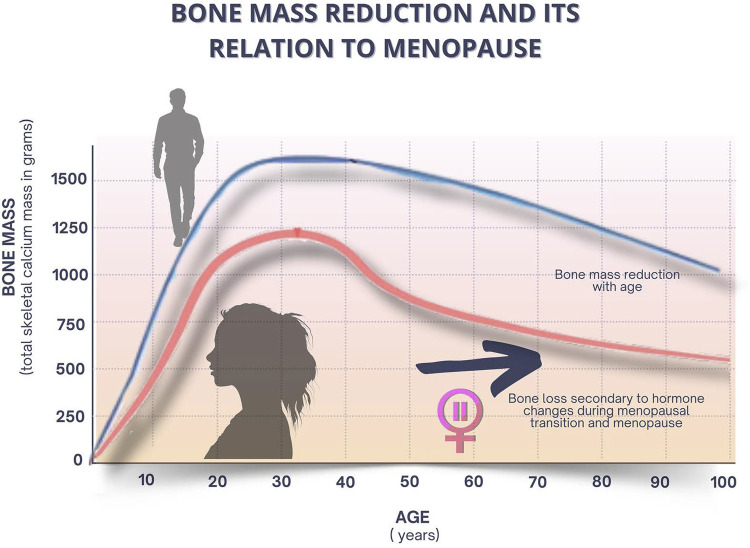
Bone mass in men versus women. Men have a reduction in bone mass as age advances, while women have a more marked decline related to menopausal transition/menopause.

Figure 5.6 illustrates bone mass in men and women, showing more marked bone loss in women after menopause.

## 6. Cardiovascular Risk and Sex Hormones

### 6.1. Introduction

Cardiovascular diseases are the major cause of women’s mortality, and there has been a constant multidisciplinary effort to change this scenario. Traditional RFs for CVD, such as SAH, DM, obesity, DLP, and smoking, are well-known in both sexes, but it is crucial to highlight specific RFs that still do not receive due attention in medical anamnesis.^180,181^ This chapter approaches the topics that should be included in the algorithms for CVR stratification of women, both cis- or not, such as: early menarche, hormonal contraception, PCOS, and use of testosterone and hormone therapy in transsexuality.

### 6.2. Early Menarche

In Brazil, the mean age of menarche is 12.4 years, and it can be influenced by race, socioeconomic level, and behavioral, climatic, and genetic factors, in addition to overweight/obesity.^182^

Menarche before the age of 10 years is considered early, but, when happening before the age of 12 years, it is already related to higher CVR, higher frequency of RFs, more CV events, mainly IHD and stroke, in addition to higher general mortality in adulthood.^183-185^

Factors related to fetal nutrition, overweight, and higher BMI in childhood have been associated with early menarche and MS during adulthood.^183,184,186^ However, after adjusting for BMI, early menarche is associated with unfavorable levels of insulin, glycemia, glycated hemoglobin, BP, and worse lipid profile in adulthood. In addition, higher risk of SAH, DM, and non-alcoholic fatty liver disease has been reported.^183,184,187^

Each year of menarche delay has been reported to cause a 2-4% reduction in all-cause mortality.^184^ However, a study with 34 000 women has shown that smoking eliminated that protection, and non-smokers had a reduction in the incidence of SAH, DM, obesity, and HF.^188,189^

The WISE study has assessed 648 North-American women, considering their duration of exposure to endogenous or exogenous estrogen (pregnancy and oral contraception, respectively). Although the authors have reported an increase in CVR in patients with early menarche, mainly before the age of 10 years, they have shown no difference in the CVD incidence according to estrogen exposure throughout life.^190^

Paradoxically, late menarche, after the age of 17 years, also seems to influence the increase in CVR.^190,191^ The hypotheses suggest that a shorter exposure to estrogen throughout life and PCOS can partially explain the increase in CVR.^180,184^ However, the subject is still controversial and requires further investigation.^188,190^

Including the age of menarche in the CVD investigation is a window of opportunity for risk stratification, enabling the implementation of more effective preventive measures, such as lifestyle change, and, when necessary, pharmacological therapy to reduce CV events.

### 6.3. Hormonal Contraceptives

The development of hormonal contraception has been acclaimed as one of the most important achievements in public health of the twentieth century. Since 1960, the “pill” has been used by millions of women to prevent unintended pregnancy and its consequences, such as unsafe abortion and all-cause maternal death.

Hormonal contraception is available in different routes of administration and the contraceptives contain either a combination of estrogen (usually ethinyl estradiol – EE) with progestin (derived from progesterone or testosterone) or progestin alone. It is worth emphasizing that the combined oral contraceptive (COC) is the most used form of reversible hormonal contraception.

#### 6.3.1. Innovation of Hormonal Contraception and Cardiovascular Risk

In the 1960s, there was scientific evidence of a “robust” association between high doses of EE contained in COCs and the occurrence of thromboembolism, MI, and stroke.^192^

The major adverse CV effects associated with COCs are assumed to derive from estrogen action on lipid metabolism and hemostasis, both mediated by the liver receptors of estrogen.^193,194^ This encouraged the production of COCs containing lower doses of EE, aimed at reducing the CVR.^195^

The progestins derived from testosterone (norethindrone-1^st^ generation, levonorgestrel-2^nd^ generation) contained in COCs are associated with androgenic side effects with negative impact on the metabolism of carbohydrates and lipids, while the progestins derived from progesterone and spironolactone (chlormadinone, drospirenone-4^th^ generation) more specifically bind to progesterone receptors, thus reducing the androgenic, estrogenic, and glucocorticoid receptor interactions.^196^

Recently, novel formulations of COCs, estradiol valerate with dienogest-4^th^ generation and estradiol with nomegestrol acetate-4^th^ generation, have replaced the synthetic hormones with steroidal components analogue to endogenous molecules. This innovation assumes that those compounds more similar to natural hormones should have a more favorable CV safety profile.^197^

#### 6.3.2. Hormonal Contraceptives and Cardiovascular Disease

The association of the use of hormonal contraceptives and CVD is a complex subject and reason for debate and investigations. The lack of high-quality evidence and studies have resulted in inconsistent and conflicting conclusions.^198^ Therefore, some studies that deserve to be highlighted, despite their limitations, were selected for this document.

The risk of MI in COC users has been investigated in a meta-analysis of 24 studies, which showed RR of 1.6 (95% CI, 1.3-1.9) proportional to the increase in the EE dose (> 50 mcg) but not modified by the type of progestin (drospirenone *versus* levonorgestrel).^199^ A second meta-analysis of 11 studies performed after the 1990s has reported a 1.7-time higher aggregate risk of MI among COC users.^200^

Specific study on the risk of thromboembolism, stroke, and MI among users of COCs with EE < 30ug has found that, for the same dose of EE, the risk of VTE was higher with desogestrel-3^rd^ generation and gestodene-3^rd^generation, as compared to that with levonorgestrel. This result was not observed for arterial thromboembolism. In addition, for the same type of progestogen, the EE dose of 20 µg *versus* EE of 30-40 µg was associated with lower risk of events.^201^

The association between SAH and COC use has been studied in a meta-analysis of 24 studies with 270 284 participants. It showed RR of 1.47 (95% CI, 1.25-1.73) for COC users and a linear dose-response relation of 13% (RR 1.13; 95% CI, 1.03-1.25) increase of ‘new’ cases of SAH for each 5 years of COC use.^202,203^

A meta-analysis on the relation between stroke and COC use has shown risks according to the following variables: 1) EE dose: for each 10 μg increase, there was an increase in OR of 1.19 (95% CI, 1.16-1.23) for stroke; 2) duration of COC use: for each 5 years of COC use, there was an increase in OR of 1.20 (95% CI, 1.05-1.37); and 3) time after COC suspension: for each 5 years of COC use interruption, OR was 0.82 (95% CI, 0.68-0.98), reinforcing the already known correlation between estrogen and CVR.^204^

Regarding the progestin-only contraceptives, a systematic review and meta-analysis has shown that the aggregated RR for MI and stroke were 0.98 (95% CI, 0.66-1.47) and 1.02 (95% CI, 0.72-1.44), respectively.^205^

Evidence has shown a higher risk of VTE in users of COCs with high doses of EE (>50 μg) as compared to the ones with low-dose formulations (<35 μg). There is reasonable evidence that users of 3^rd^-generation COCs have a slightly higher risk of VTE than users of 2^nd^-generation COCs. The administration of the hormonal combination, both patch and vaginal ring, had no different results for the risk of VTE as compared to the oral one.^206-208^

Regarding progestogen-only, the stratified analysis according to the route of administration showed that the injectable posed a higher risk of VTE (RR 2.62; 95% CI, 1.74-3.94), which was not observed for the oral route (RR 1.06; 95% CI, 0.7-1.62), and a decreased risk with the hormonal intrauterine device (HIUD) with levonorgestrel (RR 0.53; 95% CI, 0.32-0.89). No effect of the use of progestogen-alone on BP has been found, but there was a trend of increased risk for DM for the injectable route.^209^

#### 6.3.3. Underlying Situations of Cardiovascular Risk and Use of Contraceptives

There is evidence that smoking, age over 35 years, obesity, presence of inherited thrombophilias, such as mutations of factor V Leiden and prothrombin G20210A, and deficiencies of proteins C, S or antithrombin already pose intrinsic risks for thrombotic events, contraindicating COCs.^206,210^

Publications on the use of hormonal contraceptives in women with CVDs are limited. A prospective study over 39 months with women with structural cardiac lesions (rheumatic and congenital) has reported CV events (SAH and transient ischemia) in 11.5% of users of COCs with EE 30 μg + desogestrel and in 7.4% of users of injectable depo-medroxyprogesterone quarterly.^211^

In conclusion, the prescription of hormonal contraception in situations posing risks for CVD should meet the WHO eligibility criteria^212^ (Table 6.1).

**Table 6.1:** WHO eligibility criteria for the use of hormonal contraceptives in women with cardiovascular diseases (CVDs)

Condition	COC Patch Ring	Combined injectable	POP		Injectable DMPG	Transdermal		HIUD	
Smoking									
<15 cigarettes/day	3	2	1		1	1		1	
≥15 cigarettes/day	4	3	1		1	1			
Multiple risk factors for CVD									
e.g. advanced age, smoking, diabetes, SAH, dyslipidemia	3/4	3/4	2		3	2		2	
Hypertension									
History of SAH/BP cannot be assessed (includes gestational diabetes)	3	3	2		2	2		2	
Controlled SAH, when BP can be measured	3	3	1		2	1		1	
High SAH									
- SBP=140-159 or DBP=90-99 mm Hg	3	3	1		2	1		1	
- SBP>160 or DBP>100 mm Hg	4	4	2		3	2		2	
Vascular disease	4	4	2		3	2		2	
DVT/PE									
History of DVT/PE	4	4	2		2	2		2	
Acute VTE/PE	4	4	3		3	3		3	
VTE/PE on anticoagulant therapy	4	4	2		2	2		2	
Major surgery with long immobilization	4	4	2		2	2		2	
Thrombogenic mutations									
Factor V Leiden, prothrombin, proteins C, S, or antithrombin	4	4	2		2	2		2	
Ischemic heart disease	4	4	I	C	3	I	C	I	C
Current and history			2	3		2	3	2	3
Stroke	4	4	I	C	3	I	C	2	
Current and history			2	3		2	3		
Valvular heart disease									
Factors aggravating valvular diseases (pulmonary hypertension, risk of atrial fibrillation, history of infective endocarditis)	4	4	1		1	1		2	

BP: blood pressure; COC: combined oral contraceptive; CVD: cardiovascular disease; patch, transdermal patch; ring, vaginal ring; DBP: diastolic blood pressure; DPMG: depo-medroxyprogesterone; DVT: deep venous thrombosis; HIUD: hormonal intrauterine device with levonorgestrel; PE: pulmonary embolism; POP: progesterone oral pills; SAH: systemic arterial hypertension; SBP: systolic blood pressure; VTE: venous thromboembolism; C: continuous use; Category 1: Condition with no restriction on the use of the method; Category 2: Condition in which advantages exceed disadvantages in the use of the method; Category 3: Condition in which the risks exceed advantages in the use of the method; Category 4: Condition with unacceptable risk to health due to the use of the method.

#### 6.3.4. Late Cardiovascular Disease and Use of Hormonal Contraceptives

A prospective study of 11.8 years, including 161 017 women, has shown that the history of COC use, independently of the traditional RFs, was associated with a lower risk for all-cause death and incidence of late CV events, such as CAD, MI, HF, and AF, after adjusting for age (all with statistical significance of p<0.05).^212-214^

The treatment with COCs of reproductive disorders during the fertile years is assumed to have a protective effect on the CV system. This is in accordance with evidence showing that anovulatory cycles caused by hypoestrogenism and hypothalamus dysfunction increases the risk of coronary atherosclerosis and CV events.^215^

## 6.4. Polycystic Ovary Syndrome

Infertility is a condition that affects 9-18% of the population at reproductive age, being a well-known CVRF for postmenopausal women. One of its most common causes is PCOS that can cause an elevation in androgenic hormones and MS.^216^ PCOS affects 6-10% of women at reproductive age worldwide, being defined as the presence of two of the following criteria: excess of androgens, presence of ovarian cysts, and oligo-anovulation.^217^

Women with PCOS are at increased risk for metabolic disorders known to be related to atherosclerosis and CVD, such as obesity, SAH, glucose intolerance, DLP, and sleep obstructive apnea. Dyslipidemia is the most frequent metabolic abnormality in PCOS, usually showing low HDL-c levels and high TG levels, but also increased LDL-c levels.^218^

The biological mechanisms linking PCOS to increased CVR are multifactorial. Insulin resistance, highly prevalent among women with PCOS, both thin and obese, increases the adipose tissue lipolysis, leading to DLP and vasoconstriction, which is mediated by the reduction in NO synthesis in the vascular endothelium. Hyperinsulinemia can also elevate sympathetic activity with consequent increase in the renal water retention and in BP, while the defects in the insulin secretion and peripheral action contribute to an increased risk for DM.^219^

The relation of PCOS to atherosclerotic disease was demonstrated in 2014 in the CARDIA (*Coronary Artery Risk Development in Young Adults*) study,^220^ which assessed the presence of hyperandrogenism and anovulation and correlated them with the development of coronary artery calcifications and increased carotid intima-media thickness, classifying those women as having subclinical atherosclerotic disease (OR 2.70; 95% CI, 1.31-5.60).

A more recent meta-analysis with ten studies and 166 682 women included has shown an increased risk for MI, IHD, and stroke (OR 1.66; 95% CI, 1.32-2.08), but for neither CV nor all-cause mortality in patients with PCOS.^221^

A meta-analysis with 32 studies has shown that women with PCOS had a 1.3-time higher risk of developing a combination of CVDs, and the increased risk persisted when IHD and stroke were assessed separately, but no increase in CV mortality was observed.^222^

Physicians of postmenopausal women need to know their patients’ fertility history, because the presence of PCOS can change their CVRs. In addition to that, their CVRF assessment should include the patients’ obstetrical history, presence of early menopause, depression, autoimmune diseases, and other emerging RFs for an accurate and individualized risk determination.

## 6.5. Use of Testosterone for Women

Testosterone is an important sex steroid that acts directly as an androgen or indirectly as a precursor of estrogen in women.^223^ The CVDs are the major cause of death worldwide, and, although women are less affected than men during their reproductive phase, the CVR increases in the MT because of the loss of the ovarian estrogen protection and circulation of androgens.^224^

It is still inconclusive whether the use of exogenous testosterone poses risk or danger of CVD to women, because no randomized clinical trial has been conducted to assess those effects. Observational data regarding endogenous testosterone and CVD morbidity and mortality in women are inconsistent, some being positive, some negative, and others showing no association, maybe because of differences in the studies’ designs.^225^ Until recently, the use of androgens in women has been considered skeptically. Hyperandrogenism is typical of women with hirsutism and PCOS, being associated with increased CVR and metabolic disorders.

The *Women’s Health Study* has reported a relation between high levels of free androgens and increased IHD risk in postmenopausal women, although the association was not independent of other CVRFs. In the *Cardiovascular Health Study*, an association was found between high levels of testosterone and risk for CAD. However, a hypoandrogenic state is also harmful to CV health. Low levels of androgens are associated with atherosclerosis, CAD, and damage to the arterial wall of elderly of both sexes.^226^It has been proposed that low levels of serum testosterone in women is harmful to CV health, given that testosterone in physiological concentrations has favorable effects on the vasomotor tonus, endothelial function, and peripheral vascular resistance.^227^

A substudy of the ASPREE trial, the SHOW study, performed in women from Australia, aged at least 70 years, who were not receiving hormonal or steroid therapy, has compared lower *versus* higher concentrations of sex hormones, assessing major adverse CV events and all-cause mortality as primary endpoints. The serum levels of testosterone and DHEA above the lowest quartile in older women were associated with a reduced risk of a first major adverse CV event.^228^ Lopez *et al.*,^229^ in an observational study, have assessed the association of testosterone replacement therapy and CV outcomes in cisgender women and transgender people, in addition to determining if that association varied with the menopausal *status*. In that study, the use of testosterone replacement therapy increased the risk of CVD, CAD, and stroke in cisgender women, but not in transgender people.^229^

Currently, only one indication for testosterone supplementation is based on evidence: treatment of the hypoactive sexual desire disorder in postmenopausal women. In that scenario, the oral route should not be used because it is associated with adverse effects of the lipid metabolism, and the dose prescribed should result in physiological levels of serum testosterone close to premenopausal ones.^227^

Testosterone replacement therapy is not indicated and should not be used to improve cardiometabolic or musculoskeletal health, VMS, or mood swings, because there is no evidence to support its use in premenopausal women. In postmenopausal women, testosterone replacement within physiological levels can improve general well-being, but data are still inconclusive and there are sufficient studies on neither the impact of androgens on CV health in postmenopausal women nor if they can be used for CV treatment. There is still a large gap in scientific knowledge regarding testosterone replacement therapy, thus, robust randomized studies with specific assessment of the population groups in different scenarios are required.

## 6.6. Hormonal Therapy and Transsexuality

Transgender or gender incongruence describes the situation in which the individual gender differs from the assigned sex at birth. Around 0.6-1.1% of the world population and 2.7% of adolescents are transgender. Gender dysphoria is the discomfort between gender identity and the assigned sex at birth. Gender identity is affirmative care and can include gender-affirming hormone therapy (GAHT) and surgeries, as well as other procedures.^230^

The WHO reclassifies gender incongruence from the ‘mental health chapter’ to the new and established ‘sexual health chapter’, reflecting the current understanding of gender identity. This reclassification aims at reducing stigma and facilitating gender-affirming healthcare.^231^

The GAHT helps improve gender dysphoria and promote well-being. It is difficult to assess the CV effects of such treatments because of the variety of hormone regimens, paucity of longitudinal studies focusing on CV outcomes, and bias by confounding factors known to increase CVR in that population, such as psychological stress, smoking, alcohol abuse, HIV infection, discrimination, lower socioeconomic status, some of which offer opportunity for intervention. There is no consensus on the comparability of CVRs between transgender and cisgender individuals. In addition, medical problems prior to GAHT should be sought.^232^

Gender-affirming interventions include puberty suppression, hormone therapy, and gender-affirming surgery. This chapter focus on GAHT and its effects on CVRFs.^233^

### 6.6.1. Gender-affirming Hormone Therapy

Hormone therapy for a transgender individual is aimed at decreasing the levels of endogenous hormones and maintaining hormonal levels compatible with those of the opposite gender, mainly to obtain the secondary sexual characters of the desired gender, reducing those of the biological sex. These changes are aimed at providing physical, mental, and emotional well-being.^234^

Table 6.2 shows the major hormones involved in GAHT and their desired and adverse effects.

**Table 6.2:** Major hormones involved in gender-affirming hormone therapy and their desired and adverse effects

HORMONE	ROUTE OF ADMNISTRATION	DOSE	DESIRED EFFECTS	GENERAL ADVERSE EFFECTS
Transgender women				
Estradiol valerate	Oral	2-6 mg/day	Hormone for the changes: suppresses gonadotrophins and, thus, androgen production. These changes can be definitive and should be clarified regarding fertility	Therapy with estrogens in general: • Changes in liver metabolism • Changes in cholesterol are conflicting • Increase in triglycerides • Reduction in serum homocysteine • Prothrombotic state depending on dose and administration route • Higher incidence of MI vs. cis-women • Higher incidence of stroke vs. cis-men • Higher incidence of VTE vs. cis-women and cis-men
Estradiol	Transdermal	0.025-0.2 mg/day		
Estradiol (valerate or cypionate)	Parenteral	2-10 mg intramuscular/week		
Anti-androgens				
Spironolactone	Oral	100-300 mg/day		Anti-mineralocorticoid effect, can change blood pressure levels At high doses: potential increase in meningioma; depression and hyperprolactenemia
Cyproterone acetate	Oral	25 mg/day		
Triptorelin	Subcutaneous	3.75 mg/month		
Finasteride	Oral			
Transgender men				
Testosterone cypionate or enanthate	Parental	100-200 mg/every 4 weeks		Therapy with testosterone in general: • Adverse changes in cholesterol metabolism: increase in LDL, reduction in HDL, mild increase in triglycerides • Increased insulin resistance Increase in serum homocysteine • Increase in medio-intimal thickness with continuing use • Despite the reported adverse effects, it does not seem to increase CV outcomes in tras-men in most series. However, some studies have shown an increase in MI in trans-men vs. cis-gender in general
Testosterone decanoate	Parental	1000 mg/every 12 weeks		
Testosterone gel 1.6%	Transdermal	50-100 mg/day		
Testosterone patch	Transdermal	2.5-7.5 mg/day		

CV: cardiovascular; MI: myocardial infarction; VTE: venous thromboembolism.

A – Female transsexual therapy with estrogens

Estradiol is the hormone for the changes, suppressing gonadotrophins, and, thus, the production of androgens. These changes can be definitive and should be clarified regarding fertility.

Ethinyl estradiol is a synthetic estrogen that has been widely used. However, because of its prothrombotic potential and possible role in CVDs, most hormone regimens currently use oral, cutaneous, or intramuscular estradiol valerate. Studies comparing the long-term safety and efficacy between the different estradiol formulations lack.^230,235^

B – Androgen Deprivation Therapy

Spironolactone reduces the synthesis of testosterone and its action on the androgen receptor. Gonadotropin-releasing hormone agonists, such as triptorelin and cyproterone acetate, block the androgen receptors. Finasteride improves androgenetic alopecia in transgender women.^236^

C – Male Transsexual Therapy with Testosterones

The major hormone treatment is testosterone, aimed at suspending menstruation and inducing virilization, deep voice, and acquisition of an android body type. These changes can be definitive and should be clarified regarding fertility. Weight, BP levels, and hematocrit should be monitored, because erythrocytosis can occur with the use of some types of testosterones.^231,237^

### 6.6.2. Transsexuality and Cardiovascular Health

In all literature referring to transgender people and CVR, there is concern about the confounding RFs present in almost 50% of that population: smoking, stress, sleep disorders, and especially psychosocial determinants. It is worth noting that GAHT often begins in adolescence, enlarging the exposure time to hormones and psychosocial RFs in that population. Thus, not only the cardiologist, but all the multidisciplinary team involved in this scenario should approach and intervene positively on the CV health milestones formed by qualitative variables of difficult analysis and impact assessment, but highly important. There are several knowledge gaps in this subject, thus further and well-conducted research is required.^181,231^

## 7. Current Recommendations for Menopausal Hormone Therapy

In the past 20 years, longitudinal studies with women transitioning through menopause have emphasized the increase in the risk of CVD in that period. This increased risk results from endogenous changes of sex hormones and unfavorable changes in body fat distribution, lipids, and lipoproteins, as well as in the functional measures of vascular health in MT, described in the previous chapters. These data emphasize the importance of monitoring to enable CV health interventions for women in MT.^238^

Since the end of the 1980s, there has been a change in the short-term estrogen therapy prescription for menopausal symptoms, favoring the long-term prescription (longer than 5 years) to prevent CVD, especially CAD. This prevention strategy has been based on more than 30 observational studies, most of which showing a protective effect of estrogen regarding CVDs.^238^ In addition to observational data, angiographic and postmortem studies have suggested that estrogen has an antiatherogenic effect.^239,240^

Although the initial observational studies had suggested benefits from MHT for primary and secondary prevention of CVD, this has not been confirmed in the subsequent large clinical trials. The WHI study was a set of clinical trials, including two trials of estrogen therapy, in healthy postmenopausal women aged 50-79 years. One of the trials testing the continuous combined regimen of estrogen-progestogen [conjugated estrogen, 0.625 mg, and medroxyprogesterone acetate, 2.5 mg/day] *versus* placebo in more than 16 000 women was discontinued because of the increased risk of breast cancer, stroke, CAD, and VTE during the mean 5.2-year follow-up.^1^ Another study with conjugated estrogen, 0.625 mg, *versus* placebo in approximately 11 000 women with hysterectomy has also been discontinued because of the increased risk of stroke and lack of benefit for the CV health of menopausal women.^241^

Factors including the advanced age of the WHI population, as well as the type, administration route, and dose of estrogen, have been considered to explain the discordant results.^242^Other factors proposed to explain the adverse results observed were high BMI, associated comorbidities, such as diabetes, family history, smoking, and the time from MT to the beginning of MHT. Subsequent studies have shown that the absolute risk of an adverse event (breast cancer, CAD, stroke, or VTE) was low, 19 additional events per year per 10 000 women on MHT *versus* placebo.^243^

In addition, estrogen is the most effective treatment available to relieve menopausal symptoms, mainly the VMS. The MHT, estrogen alone or combined with progesterone, is currently indicated to treat menopausal symptoms. The long-term use to prevent diseases is no longer recommended.^244^

Data from a primate model, observational studies in postmenopausal women, meta-analysis of clinical trials, coronary angiographic study, and secondary analyses of the WHI have suggested that the time when the MHT starts is an important factor to influence the subsequent CV risk. The use of MHT in the first years of menopause does not seem to be associated with an increased risk of CVD as compared to that of older women in postmenopause.^238^The population of the WHI study was older (mean age, 63 years) than that of most observational studies. More advanced age at the MHT beginning could be associated with more subclinical atherosclerosis at the beginning of the study, with advanced or complex atherosclerotic lesions that can be more susceptible to the prothrombotic and pro-inflammatory effects of estrogen, especially when used orally. In contrast, starting the MHT right after menopause might not cause damage (or might be beneficial).

A meta-analysis of 19 trials on oral MHT (including the WHI) with over 40 000 postmenopausal women, with analyses of subgroups in women beginning MHT within 10 years of menopause onset, has reported lower risk of CAD and CVD mortality.^245^ A meta-analysis from 2017 with similar trials has concluded that the risks of the prolonged use of MHT to prevent chronic diseases outweighed any benefits.^246^

Because of the increase in women’s life expectancy, it is estimated that 40% of their lifespan will be spent in postmenopause. Considering that:

- the VMS are associated with worse levels of RFs for CVD and subclinical atherosclerosis measurements,
- sleep disorders are a common complaint during MT, being associated with higher risk of subclinical CVD and worse indices of CV health,
- depression more often occurs during the perimenopausal and postmenopausal years, being related to VMS and the occurrence of CVD,
- the increase in central adiposity during MT is associated with increased risk of mortality, even among those with normal BMI,
- the increase in lipids (LDL-c and apolipoprotein B), the risk of MS, and vascular remodeling are more affected by MT than by aging,
- there is a higher risk of fractures due to bone mass loss associated with MT,

the SBC, FEBRASGO, and SOBRAC recommend in **FAVOR** of adopting MHT for symptomatic menopausal women without contraindications. (**Strength of recommendation in FAVOR. STRONG recommendation. Level of certainty: HIGH**).^64,94,132,238,242,247-254^

The MHT consists in the administration of different sex hormones that should be individualized according to the risks and benefits for each woman. The many formulations, doses, and administration routes of MHT have high efficacy to relieve postmenopausal symptoms and were discussed in the preceding chapters. (**Strength of recommendation in FAVOR. STRONG recommendation. Level of certainty: HIGH**). There is neither pre-established maximal age nor duration for the use of MHT, and the decision to continue or interrupt MHT should be based on the persistence of indications and no change in the risks. (**Strength of recommendation in FAVOR. STRONG recommendation. Level of certainty: MODERATE**).^64,179,244,247,255-259^

The primary indication for systemic MHT is the treatment of VMS, and this is the most effective therapy, considered gold standard for the relieve of those symptoms. Vaginal estrogen-only therapy is effective for the treatment of the genitourinary syndrome symptoms only. In addition, MHT is indicated to prevent bone mass loss and to reduce the risk of fractures. For women with POF, MHT should be used at least close to the age of 50 years, which is the mean age of menopause occurrence. (**Strength of recommendation in FAVOR. STRONG recommendation. Level of certainty: HIGH**).^34,143,260-273^

The literature supporting the critical role of the time of MHT beginning, before the age of 60 years or within 10 years of menopause onset, seems to be associated with the reduction of CVD risk. Evidence suggests that the effects of MHT on the progression of atherosclerotic events and CVD vary according to the age MHT is initiated or the time from menopause to MHT beginning. The beneficial effects on the CVD outcomes and all-cause mortality can occur when MHT is initiated in women <60 years of age or within 10 years of menopause onset, but the effects can be null or harmful when MHT is initiated at more advanced ages or after a longer time from menopause.

Numerous observational studies before 1991, that recommended the use of MHT close to the beginning of menopause, have reported a reduction in the rates of CAD in MHT users.^273^ Analysis of prospective observational studies has reported a RR of CAD events of 0.50 (95% CI, 0.43–0.56).^42^ Analyses of the *Nurses’ Health Study,* with women aged 30-55 years at the study baseline, have shown lower risk of mortality among current users as compared to those who never used MHT (RR, 0.63; 95% CI, 0.56-0.70), and a higher reduction in mortality in those at higher risk.^274^ Case-control and cross-sectional studies have also found a reduction in morbidity from CAD with MHT in women with CAD defined on coronary angiography.^273,275-277^

The MHT should be initiated in the “window of opportunity”, that is, within 10 years of menopause onset and/or before the age of 60 years**. The decision to initiate MHT, its dose, regimen, and duration should be individually made after discussing the benefits and risks with each patient and after providing written information on the product selected. This should be considered in the context of the global benefits obtained with the use of MHT, including control of symptoms and better quality of life, and weighing the potential CV and bone benefits associated with the use of MHT. (**Strength of recommendation in FAVOR. STRONG recommendation. Level of certainty: HIGH**). The combined use of estrogen and progestogen is not recommended for the primary prevention of chronic conditions in postmenopausal asymptomatic women. In addition, the use of estrogen alone is not recommended for the primary prevention of chronic conditions in postmenopausal women with hysterectomy. The effects of MHT on the risk for CVD vary depending on the time it is initiated. In healthy women, when initiated in the window of opportunity, MHT can have favorable effects on the risk of CVD. However, there is no indication to start MHT aiming at primary CV prevention in multiple scenarios. However, beginning MHT after the age of 60 years or more than 10 years after menopause onset can increase the absolute risk for CAD, VTE, and stroke. (**Strength of recommendation in FAVOR. STRONG recommendation. Level of certainty: HIGH**).^29,42,255,283-290^

Cessation of ovarian function has great impact on the cardiometabolic RFs, such as increases in BP, cholesterol, body mass, and blood glucose. Obesity is strongly related to BP elevation in women.^291,292^ In menopause, in response to BP elevation, women have more intense microcirculation abnormalities, higher occurrence of chronic kidney disease, changes in coronary microcirculation, and LV concentric hypertrophy.^291,292^ In women, SAH is a strong RF for acute myocardial infarction (AMI), HF with preserved (more common in women) and reduced ejection fraction, peripheral arterial disease, stroke, and cognitive decline. In later menopause, significant arterial stiffness acceleration has been observed, which can contribute to an even higher CV risk.^293^

Estrogen-replacement doses have little effect on BP. The WHI trial with combined estrogen-progestin has reported only a slight increase (1.5 mm Hg) in systolic BP as compared to placebo.^1^ In addition, the WHI trial with estrogen only has reported a similar difference of 1.1 mm Hg between the groups on hormone or placebo.^241^ Likewise, the *Postmenopausal Estrogen/Progestin Interventions* (PEPI) study has reported that estrogen, combined or not with progestogens, had no effect on BP. These findings contrast with the frequent BP elevation observed when higher doses of synthetic estrogen (EE) are administered for oral contraception.^291^Women with controlled SAH and moderate to intense VMS can use MHT through any route, but preferably transdermal estrogen therapy (by use of gel or patches) in the presence of obesity, DLP, DM, and MS. (**Strength of recommendation in FAVOR. STRONG recommendation. Level of certainty: MODERATE).**

All peri- and postmenopausal women, including those on MHT should undergo periodical screening mammography according to the current screening guidelines. FEBRASGO suggests mammography be performed before prescribing MHT. In addition, quantifying TC and its subfractions, TG, and fasting glycemia helps choose the best MHT administration route. Other complementary tests might be necessary depending on the findings of clinical history, physical exam, and RFs. In addition, postmenopausal women with intermediate risk stratification for CVD might need complementary assessment to better individualize MHT.^179,294^

Observational evidence suggests a protective effect of MHT against CVD. However, the WHI and other studies on MHT do not support such findings. The analysis of 3 trials (n = 18 085) has shown no significant difference in the risk of CAD events in individuals treated with estrogen plus progestin as compared to placebo (2.8% *versus* 2.6%; RR, 1.12; 95% CI, 0.94-1.33) during a mean 4-year follow-up. Likewise, a grouped analysis of 3 trials (n = 11 310) has found no significant difference in coronary events between the use of estrogen alone and placebo (RR, 0.95; 95% CI, 0.79-1.14) during a mean 4.1-year follow-up. The WHI has reported an increased risk for stroke with the use of estrogen, both alone and in combined therapy with progestogen. The risk for stroke was significantly higher in individuals randomized to receive estrogen plus progesterone as compared to those randomized to placebo (1.9% *versus* 1.3%; HR, 1.37; 95% CI, 1.07-1.76). Likewise, women who received only estrogen had a statistically higher risk of stroke as compared to those who received placebo (3.2% *versus* 2.4%; HR, 1.35; 95% CI, 1.07-1.70). Systemic MHT is not recommended for women with manifest CVD, history of AMI or stroke.^279^ (**Strength of recommendation in FAVOR. STRONG recommendation. Level of certainty: HIGH**).^283,295^ Vaginal estrogen therapy for the treatment of menopausal genitourinary syndrome can be used in women with known CVRF or established CVD and requires no addition of progestogen in those with hysterectomy.^29,42,179,255,284-290,294^

For women with contraindication or who do not want to undergo MHT, nonhormonal therapies with proven effectiveness*** can improve VMS. (**Strength of recommendation in FAVOR. STRONG recommendation. Level of certainty: HIGH**).

Five trials have reported risk of thromboembolism with the use of oral MHT. In the WHI (n = 16 608), individuals randomized to receive conjugated estrogen plus medroxyprogesterone had an increased risk of venous thrombosis (1.96% *versus* 0.94%; HR, 2.06; 95% CI, 1.57-2.70), DVT (1.4% *versus* 0.8%; HR, 1.87; 95% CI, 1.37-2.54) and PE (1.0% *versus* 0.5%; HR, 1.98; 95% CI, 1.36-2.87) as compared to those receiving placebo.^132,298^ Other studies have reported few thromboembolic events or had results consistent with those of the WHI.^283,295^ In the WHI (n = 10 739), individuals randomized to receive estrogen alone had an increased risk of DVT (1.6% *versus* 1.0%; HR, 1.48; 95% CI, 1.06-2.07). The risk of PE was higher in the estrogen group than in the placebo group, but the results were not statistically significant, although the confidence interval was (0.98% *versus* 0.72%; HR, 1.35; 95% CI, 0.89-2.05).^132^For women with history of VTE, MHT is not usually recommended, but, depending on the cause of the event, should MHT be indicated, the transdermal route would pose the lowest risk. (**Strength of recommendation in FAVOR. STRONG recommendation. Level of certainty: MODERATE**). Decompensated liver disease, bleeding of unknown cause, and systemic lupus erythematosus with high thrombotic risk are also contraindications with weak level of evidence. **(Strength of recommendation in FAVOR. WEAK recommendation. Level of certainty: LOW**).

The trials on MHT have reported the risk of breast cancer as one of the major adverse results of the treatment. In the WHI, 16 608 women were randomized to receive estrogen plus progestin or placebo. There was significantly increased risk of breast cancer in the group receiving MHT as compared to that receiving placebo (2.4% *versus* 1.9%; HR, 1.24; 95% CI, 1.01-1.53),^132^ and the risk persisted during follow-up.^303-305^In addition, in the WHI, in a 20.3-year follow-up, the point estimate of the risk of mortality from breast cancer was higher in the estrogen plus medroxyprogesterone group than in the placebo group, although the difference was not statistically significant (HR, 1.35; 95% CI, 0.94-1.95).^304^ Four studies have reported the effects of estrogen alone on breast cancer; however, only the WHI has followed participants up for more than 3 years. With a 20.7-year follow-up, the WHI has reported a lower risk of invasive breast cancer in the group receiving estrogen alone as compared to that receiving placebo (HR, 0.78; 95% CI, 0.65-0.93).^1^ Moreover, in the WHI, in the 20.7-year follow-up, the group on estrogen had a lower risk of mortality from breast cancer than that on placebo (HR, 0.60; 95% IC, 0.37-0.97).^304^The risk of breast cancer associated with MHT is low, with less than one additional case per 1000 women per year of use. (**Strength of recommendation in FAVOR. STRONG recommendation. Level of certainty: HIGH**).

In 16 608 women, MHT was associated with a slight increase in the risk of breast cancer with the use of 0.625 mg/d of conjugated equine estrogen plus 2.5 mg/d of medroxyprogesterone acetate as compared to placebo (0.38%/year for conjugated equine estrogen plus medroxyprogesterone acetate *versus* 0.30%/year for placebo).^1^ In 10 738 women, treatment with 0.625 mg/d of conjugated equine estrogen alone did not show that outcome (0.26%/year for conjugated equine estrogen alone *versus* 0.33%/year for placebo).^306^ Contraindications to MHT are as follows: hormone-dependent neoplasms, such as breast cancer, breast cancer precursor lesions, endometrial cancer, history of coronary and cerebrovascular diseases. (**Strength of recommendation in FAVOR. STRONG recommendation. Level of certainty: HIGH**).

Natural hormones constituted by preparations of estradiol and micronized progesterone are approved by the Food and Drug Administration (FDA) and are available through medical prescription. In contrast, compounded bioidentical hormone preparations are not FDA-approved and should be avoided because neither their safety nor efficacy have been tested and they are not monitored for quality.^307^ In 2020, a report from the National Academies of Sciences, Engineering, and Medicine concluded that compounded bioidentical hormone preparations were not properly labeled with usage instructions, contraindications, and potential adverse effects, lacking reliable information on safety, efficacy, and product-to-product variability.^306,307^ Most marketing claims on safety and efficacy of compounded bioidentical hormone preparations are not supported by properly controlled studies. The use of compounded bioidentical hormone preparations (for example, dehydroepiandrosterone, estradiol, estradiol cypionate, estriol, estrone, pregnenolone, progesterone, testosterone, testosterone cypionate, and testosterone propionate) should be restricted to individuals with documented allergy to active pharmaceutical ingredients or excipients of medications approved by the regulatory agencies. Patients should be informed about the risks inherent in the lack of regulation of the compounded bioidentical hormone preparations by regulatory agencies. The compounded bioidentical hormones in patches or other forms and the so-called “hormone modulation” are not recommended because they lack scientific evidence of efficacy and safety.^306,307^ The SBC, FEBRASGO, and SOBRAC **are AGAINST the adoption of those therapies. STRONG recommendation. Level of certainty: VERY LOW**.

Patients should be informed that acupuncture, relaxation therapy, phytoestrogens, exercises and *black cohosh* are not significantly better than placebo to relieve VMS.^308-311^In clinical trials on therapies for VMS, up to 50% of the participants responded to placebo.^312^Thus, control with a placebo group is necessary to assess the efficacy of potential treatments for VMS.^306^

Absolute increases in the risk associated with hormone therapy are smaller for women starting the treatment close to menopause onset. Higher safety (regarding the risk of stroke or CAD) of transdermal preparations of estradiol as compared to conjugated equine estrogen plus medroxyprogesterone acetate by oral route has been suggested in large observational studies, but there is no randomized, controlled, multicenter clinical trial on that.^306^ It is worth noting that new clinical trials approaching unanswered questions, such as ‘does age or timing of initiation of MHT affect differently the health outcomes?’; ‘can the benefits and harms of MHT vary in population, racial and ethnic groups?’; ‘do socioeconomic barriers that imply higher risk for certain chronic conditions (DM, stroke, chronic CAD) alter the results of MHT?’; ‘how do the benefits and harms of the different MHT formulations and durations compare?’.^255^

**Figure 7.1:**
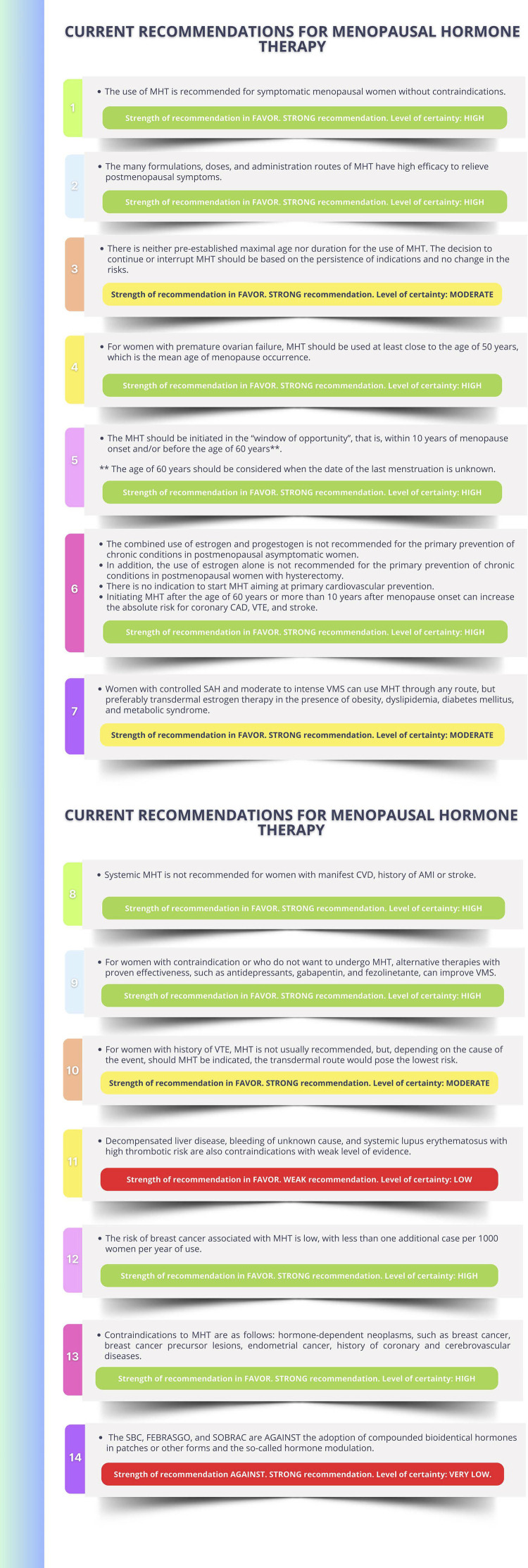
Current recommendations for menopausal hormone therapy.

The current recommendations for MHT are summarized in Figure 7.1.

## 8. Contemporary Evidence of Hormonal Therapy in Women

### 8.1. What Should be Used: Types, Doses, and Routes of Administration

The MHT consists in the pharmacological administration of sexual steroids, especially estrogens and progestogens, to women in MT or postmenopause, aimed at relieving symptoms or preventing health problems.^313^

The International Menopause Society (IMS) recommends that the use of the term ‘class effect’ be avoided, because of the large number of possibilities due to differences in hormones, routes of administration, doses, and regimens.^313^

The most important steroid in MHT is estrogen because it relieves menopausal symptoms and protects against osteoporosis. The role of progestogen is to prevent the endometrial proliferation caused by estrogen, thus preventing the increase in the endometrial cancer risk.^179^

The most frequently used estrogens in MHT are estradiol, identical to the endogenous one from the molecular viewpoint, and conjugated estrogens. Estradiol is available in the form of 17-beta-estradiol or estradiol valerate.^179,314^ Another drug used for MHT is tibolone, which is a synthetic progestogen, but its metabolites have estrogenic, androgenic, and progestational effects.^315^

Regarding progestogens, the most frequently used are the synthetic ones, such as norethisterone, dydrogesterone, drospirenone, nomegestrol acetate, and medroxyprogesterone acetate. In addition, there is micronized progesterone, identical to natural progesterone.^179^

Regarding MHT administration route, the key question refers to estrogen, whose routes can be oral, transdermal, or vaginal.^179^ The vaginal route is meant for local effects in the genital system, with no significant systemic effects,^316^ while the oral and transdermal routes have systemic effects.^179^

After absorption in the digestive tube, estrogen goes to the liver through the porta system, affecting the hepatic protein synthesis (for example, of the coagulation factors). After this hepatic pass, estrogen finally reaches the systemic circulation. When delivered through the transdermal route, estrogen is distributed to the systemic circulation right after absorption, reaching the liver only later. Thus, the estrogen’s oral administration has higher liver impact than the transdermal route, and this phenomenon has been called the ‘hepatic first-pass effect’.^317^

This phenomenon explains the fact that oral estrogen favors some increase in the serum levels of TG, but also causes a higher increase in HDL-c associated with a higher reduction in LDL-c than that observed with the transdermal route.^318^

Case-control studies have shown an increase in the thrombosis and VTE risks associated with oral estrogen, but no increase with the transdermal route.^319-321^ The last of those studies has reported RR of 1.40 (CI, 1.32-1.48) associated with oral estrogen, while, with transdermal route, RR was 0.96 (IC, 0.88-1.04).^321^

Regarding the hormone dose used, the lowest effective doses are currently preferred, the highest doses being reserved for patients who do not respond sufficiently.^179,313^ One of the studies assessing different doses of oral estradiol has reported that doses of 2 mg, 1 mg, and 0.5 mg were better than placebo to relieve VMS, but 0.25 mg was not; thus, the lowest effective dose of oral estradiol is 0.5 mg.^322^ Transdermal estradiol in Brazil is available as patches, at the doses of 25 mcg and 50 mcg, and as gel, at the doses of 0.5 mg/day to 3.0 mg/day.^314^

### 8.2. How to Prescribe MHT

The IMS, the North American Menopause Society (NAMS), and the SOBRAC agree that the treatment of menopausal symptoms, as well as the prevention of osteoporosis, is the prime indication for MHT. For the menopausal genitourinary syndrome, vaginal estrogen is preferred.^179,313,323^

**Figure 8.1:**
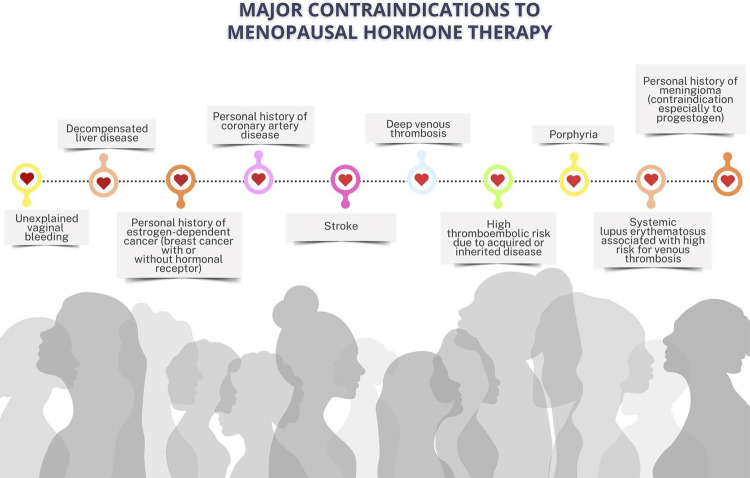
Major contraindications to menopausal hormone therapy.179,323

In addition, patients with POF have indication for MHT, in the absence of contraindications.^179^Figure 8.1 shows the major contraindications to MHT.^179,323^

According to the SOBRAC, the prescription of MHT should be preceded by the assessment of a mammography performed within 12 months from the prescription, as well as the quantification of TC and its subfractions, TGs, and fasting glycemia. Other complementary tests might be necessary depending on the clinical history and physical exam findings. Other screening gynecological tests should be requested according to specific guidelines and not because of the likelihood of beginning MHT.^38,323^

### 8.3. When and for How Long MHT Should Be Prescribed

The WHI was a large study that randomized postmenopausal women to receive MHT by use of conjugated estrogens with or without medroxyprogesterone acetate or placebo. That study has revealed that MHT associated with a higher risk of CV events,^1^ differently from that reported in the *Nurses’ Health Study*, an observational study.^324^

It is worth to carefully assess the WHI population: over two-thirds of the women were 60 years or older at the time of randomization and a significant part was 70 years or older. Only a few women were within 10 years of menopause onset at the beginning of MHT in that study. The large majority had neither moderate nor intense VMS.^325^

It has been reported that MHT initiated in the first years from menopause onset could decrease the progression of atherosclerosis^281^ or even reduce CV outcomes.^325^

It is worth noting that, in the *Nurses’ Health Study*, the women on MHT, on average, had shorter time since menopause onset and were more symptomatic than those in the WHI, similarly to the real daily practice of clinicians.^326-328^

In the WHI, when analyzing the subgroups according to the years since menopause at MHT initiation (less than 10 years, between 10 years and 20 years, and more than 20 years), the authors have reported an increase in CVR with statistical significance only in those with more than 20 years.^329^

Thus, the concept of window of opportunity for beginning MHT was created, corresponding exactly to the first years of postmenopause. The NAMS recommends beginning MHT for women in the first 10 years of postmenopause or before the age of 60 years, and states that for women with indication and no contraindication and respected the window of opportunity, the benefits of MHT outweigh eventual risks.^179^ The IMS and the SOBRAC share the same opinion.^313,323^

A joint assessment of the postmenopausal time and CVR has been proposed by Kaunitz & Manson and the NAMS. If the time since menopause is shorter than 10 years and the CVR according to risk calculators classify the MHT candidate as at low risk, MHT is indicated. If the CVR is intermediate, MHT can be prescribed, but the transdermal route for estrogen should be preferred. If the time since menopause exceeds 10 years or the calculated CVR is high, MHT is not recommended. All this should only be considered in the absence of contraindications and if agreed with the patient.^35^

Regarding MHT duration, none of the most important guidelines on MHT establishes a mandatory maximal duration when MHT should be interrupted. This decision should be made at each follow-up visit based on the assessment of individualized risk-benefit ratio.^179,313,323^

### 8.4. Prescription in Different Scenarios of Cardiovascular Risk (SAH, DM, DLP)

In Brazil and worldwide, CVDs diseases are the major cause of death, accounting for one third of all-cause deaths and affecting men and women of all age groups. In women, the traditional RFs for CVD include: DM, SAH, DLP, smoking, obesity, and sedentary lifestyle.^13^ The indications for MHT approved by the menopause societies worldwide, including the Brazilian one (SOBRAC), comprise the treatment of moderate to severe VMS, menopausal genitourinary syndrome symptoms and signs, prevention of bone loss and osteoporotic fractures, in addition to hypoestrogenism due to hypogonadism, bilateral oophorectomy, and POF.^179,313,330,331^

The prescription of MHT can be challenging in patients with one or more morbidities or one of the known CVRFs that will be approached in the following paragraphs.

#### 8.4.1. Obesity

Obesity is considered a RF for VTE, and the oral use of estrogen for overweight patients has an additive effect on the increased risk for VTE, as shown in some observational studies and systematic reviews.^319,332,333^ In the WHI study, overweight and obese women randomized to use systemic MHT had a three-fold (HR, 3.80; 95% CI, 2.08–6.94) and a six-fold (HR, 5,61; 95% CI, 3,12–10,11) higher risk for VTE, respectively, as compared to those using placebo.^298^In the absence of randomized clinical trials and based on consistent data from observational studies on the low risk for VTE with transdermal MHT, this route of administration should be preferred for patients with overweight or obesity, an indication endorsed by the leading menopause societies.^334,335^

#### 8.4.2. Dyslipidemia

Menopausal transition is associated with an increased risk for CVD, attributed mainly to atherogenic DLP, central obesity, and insulin resistance, in addition to an increased risk for SAH.^13^ The AHA states that adults aged 40-75 years should have their risk for atherosclerotic CVD estimated for 10 years.^334,335^ The SBC recommends that, for women with low CVR, LDL-c should be lower than 130 mg/dL, and, if the goal is met, estrogen therapy should be preferably administered through transdermal route to prevent the phenomenon of hepatic first pass (through the oral route), and, for patients without hysterectomy, micronized progesterone is added to neutralize the estrogen’s proliferative effects on the endometrium.^13^

#### 8.4.3. Hypertension

Systemic arterial hypertension is a well-established RF for CVD and an aggravating factor of the CVR of DM in women.^13^ In the WHI study, the use of conjugated equine estrogens either associated or not with medroxyprogesterone acetate has shown an 1-1.5 mm Hg elevation in blood pressure levels.^335^ In the observational part of the WHI study, transdermal estrogen therapy had a low risk for SAH development as compared to the oral MHT regimen.^335^ Patients with non-controlled SAH (BP ≥ 180/110 mm Hg) have contraindication to initiate MHT because of the risk of stroke.

Observational data and meta-analyses have shown a reduced risk for CAD in women who initiate MHT before the age of 60 years or within 10 years from menopause onset. According to the NAMS, if SAH is controlled and the risk stratification for atherosclerotic CVD < 5%, either oral or transdermal MHT can be used. However, in situations with SAH of difficult control and moderate to severe VMS, the use of transdermal estrogen therapy is suggested in association with micronized progesterone for women without hysterectomy, because it has lower thrombogenic potential than other progestogens.^179,334^

#### 8.4.4. Diabetes

The prevalence of DM increases with the elevation in the prevalence of obesity.^13^ Menopausal hormone therapy is not contraindicated in healthy women with preexisting DM2 and may be beneficial to control glycemia when used to relieve menopausal symptoms. Menopausal hormone therapy significantly reduces the diagnosis of DM2 of recent onset,^325^ but is not approved for that indication. For women with well-controlled DM2 (HbA1c < 8%) and moderate to intense VMS, transdermal estrogen therapy, avoiding the hepatic first pass phenomenon, associated with micronized progesterone would be the best therapeutic option.^179,334,335^

#### 8.4.5. Metabolic Syndrome

Recent meta-analysis has shown that MHT reduced multiple components of the MS, such as abdominal obesity, insulin resistance, DLP, and SAH.^336^ In the WHI study, women with MS randomized to receive conjugated equine estrogen alone or associated with medroxyprogesterone acetate had a two-fold higher risk of CVD than those in the placebo group.^335^ Despite the lack of randomized studies comparing the different MHT routes of administration and CV events in women with MS, the use of transdermal MHT is recommended to relieve symptoms because that route of administration causes no hepatic first pass phenomenon.^334,335^

## 8.5. Prescription for Patients with Manifest Cardiovascular Disease

Data from a meta-analysis have shown no significant differences between MHT users and controls regarding the primary outcomes nonfatal MI, CV death, and stroke in women with CVD. In addition, the frequency of angina, HF, and transient ischemic attack did not differ between the MHT and control groups.^337^

### 8.5.1. Acute Myocardial Infarction

In general, MHT is contraindicated to women with known CAD, such as AMI, and peripheral artery disease.^335^Beginning MHT in women within 10 years from menopause onset either reduced or had no effect on the progression of subclinical atherosclerosis and coronary artery calcification in randomized and controlled trials.^179^ A study published in 2001 showed no difference in the risk of recurrent AMI or death from CVD in women after the first AMI between MHT users and non-users.^338^ The observational study ESPRIT RCT (*Estrogen for the Prevention of Reinfarction Trial*), with post-AMI women randomized to receive estradiol valerate alone or placebo for 2 years, in a 14-year follow-up, has shown no difference between those groups regarding death from CVD, stroke, or IHD in general.^338^ Current consensus guidelines do not stratify the risk of MHT based on the woman’s subtype of CVD. The American College of Cardiology argues that, for women aged 50-59 years and with history of AMI without obstructive CAD, spontaneous dissection of the coronary artery, microvascular coronary dysfunction, or coronary vasospasm, an individualized approach to MHT is necessary. Because of the supposed pathophysiological association between female sex hormones and spontaneous dissection of the coronary artery, the American College of Cardiology recommends that oral MHT should be avoided in that group, and that, after proper control of the BP levels and lipid fractions, systemic MHT can be considered.^335^ In addition, for women with risk stratification of atherosclerotic CVD > 10%, MHT should be avoided independently of the hormone route of administration.^335^

### 8.5.2. Unstable Angina

Data from a recent meta-analysis, assessing anginal symptoms and hospitalization due to stable angina in six studies, have shown that hospitalization occurred predominantly in the first 2 years after randomization, with nonsignificant trend of being higher in the MHT group in an observational study. And, in five randomized clinical trials, there was no difference in angina between the MHT and control groups.^337^

### 8.5.3. Stroke and Transient Ischemic Accident

Previous history of ischemic stroke is a contraindication to MHT although the absolute risks of both estrogen-only and estrogen plus progestogen therapies are considered rare (<10/10 000/year).^334,335^

### 8.5.4. Heart Failure

Literature data on the use of MHT in women diagnosed with HF are scarce. In case of no response to nonhormonal treatment and if the patient has clinical improvement, ejection fraction that returns to the previous levels, and good control of modifiable factors, low-dose transdermal MHT can be indicated.^334^

### 8.5.5. Venous Thromboembolism and Pulmonary Embolism

Previous history of VTE, including DVT and PE, should be considered a contraindication to oral systemic MHT.^179,335^ Lower doses of oral MHT can confer fewer risks for VTE than higher doses, but comparative data from randomized clinical trials lack. Micronized progesterone seems less thrombogenic than other progestogens used for MHT. Transdermal MHT has not been associated with the risk of VTE in observational studies, and a systematic review has confirmed those findings, suggesting lower risk with transdermal than oral MHT. However, comparative data from randomized clinical trials lack.^179^ If DVT is diagnosed during the use of oral contraceptives or pregnancy, MHT should be avoided because of the relation of DVT and exogenous estrogen. The occurrence of a DVT event after prolonged immobilization or trauma, such as in a car accident, fall, or immediate post-operative period, indicates that the pro-inflammatory state that caused thrombosis might not be related to estrogen effects. Thus, in such situations, the transdermal route is recommended for MHT.^179,334^

### 8.5.6. Congenital Heart Disease and Post-heart Transplantation

Due to lack of literature data, cautiousness is recommended when indicating MHT in such situations, nonhormonal treatments being considered the best option.

## 8.6. Bioidentical Hormones and Pellets: What Should Be Informed

Over the past years, media coverage of hormone replacement therapy has largely focused on the use of bioidentical hormones and hormonal pellets, which has generated controversies and debates.

Bioidentical hormones are molecules with the same chemical and molecular structure of the hormones synthesized by the human body. They are exact copies of human endogenous hormones, such as estradiol, estriol, and progesterone.^179^

The term “bioidentical” has been incorrectly used to refer to new therapeutic options of “natural hormones” tailored to the needs of individual patients at compounding pharmacies to treat menopausal symptoms. However, the first issue to clarify is that the most used bioidentical hormones in MHT - estradiol, estriol, and progesterone – have been produced for years by the pharmaceutical industry at different doses and formulations and sold at commercial drugstores.

The concern is that compounded hormonal therapies may have inconsistency in dosages, quality control, and absorption, because compounded hormonal formulations might have pharmacokinetic differences and not undergo the same quality control in the different compounding pharmacies countrywide.

The prescription of MHT as hormonal pellets has been popularized on social media and networks. Hormonal pellets are capsules or spherical bodies subcutaneously implanted and can be absorbable or not.

Compounded pellets can differ regarding their hormone composition (estradiol, testosterone, dihydrotestosterone, androstenedione, oxytocin, oxandrolone, nestorone, gestrinone) released to the blood stream. These pellets are produced by compounding pharmacies, not regulated by strict rules, do not undergo rigorous assessments of safety and efficacy testing, and do not meet the requirements of the pharmaceutical industry dugs approved by drug regulatory agencies. Once no medicine leaflet is required for compounded products, the dose and type of hormone contained in each pellet are not preestablished, there is neither alert nor description of basic information, such as indications, safety testing, and possible adverse effects, as required for other industrialized medications approved by the Brazilian Agency of Sanitary Surveillance (ANVISA). There is concern about the doses and contents of these pellets, because they can expose patients to harm or risks due to overdose or hormones not indicated for MHT.^339^

A search in the largest scientific database maintained by the National Library of Medicine, PubMed, shows that there are few clinical studies on hormonal pellets for MHT use. Most of the few studies are on testosterone pellets with small case series and methodology of low grade of evidence (retrospective or observational studies), which provides no knowledge on the effects of those pellets on several relevant MHT issues, such as breast cancer risk, endometrial risks, metabolic, CV, and long-term effects, for them to be safely used in women. In addition, a large part of the pharmacokinetics of compounded pellets, such as hormone release rates, significant inter-individual variance,^340^ and difficulty in pellet reversibility in case of adverse effects, is unknown. Although there are a few studies on pellets with a large number of participants, their methodology has limitations and biases.^341,342^

However, data from PubMed have shown that all the scientific knowledge accumulated over the years with several publications on the benefits and risks of MHT related to doses, oral and transdermal routes of administration, and type of estrogen and progestogen derive from industrialized bioidentical hormones or synthetic hormones approved by the regulatory agencies. These studies are different, and their results cannot be extrapolated to the pellet route until studies specifically designed for such purpose are published.

New MHT alternatives or routes of administration are very welcome to meet the needs of a larger number of women, but they will only be recommended based on confirmed efficacy and safety mainly.

Thus, considering the lack of scientific studies that confirm the safety of compounded bioidentical hormones and of hormonal pellets, as well as the several doubts regarding their clinical effects and potential risks, these hormonal therapies are recommended by neither the Brazilian national societies, such as FEBRASGO,^343^ SOBRAC, and the Brazilian Society of Endocrinology, nor the international medical societies.

## 8.7. Additional Therapies

Some clinical situations require nonhormonal therapies to control VMS. This applies to symptomatic women with contraindications or those who, due to personal preferences, do not want to undergo MHT.

The additional therapies can be divided into pharmacological, alternative pharmacological (phytotherapy), and non-pharmacological or behavioral.

### 8.7.1. Pharmacological Therapies

Of the nonhormonal therapies to relieve VMS, the selective serotonin reuptake inhibitors and selective serotonin-norepinephrine reuptake inhibitors act through the regulation of serotonin and norepinephrine levels in the hypothalamic thermoregulator center, reducing the VMS.

Paroxetine 7.5-25 mg, citalopram 10-20 mg, escitalopram 20 mg, venlafaxine 37.5-75 mg, and desvenlafaxine 50-100 mg are the most studied antidepressants, and placebo-controlled randomized clinical trials have shown their efficacy in reducing the frequency and severity of mild to moderate VMS.^344^ Less consistent results have been obtained with sertraline and fluoxetine, which, thus, are not recommended.^345^

Gabapentin is an antiseizure drug analogue of the gamma-aminobutyric acid that crosses the blood-brain barrier and acts directly on the hypothalamic thermoregulator center. Controlled randomized clinical trials have shown its efficacy to reduce VMS at doses varying from 900 mg to 2400 mg divided into three takes. The adverse events include somnolence, dizziness, and changes in balance function, and can be a good choice for women with sleep disorders associated with VMS.^346^ Pregabalin, derived from the aminobutyric acid related to gabapentin, has been assessed at the dose of 75-150 mg; however, because of the scarcity of studies and potential adverse effects, it has not been recommended to control VMS.^347^ Oxybutynin, an anticholinergic used to treat urgency urinary incontinence at the dose of 5-15 mg/day, has been assessed in a few clinical trials and shown improvement of VMS; however, it should be cautiously used in the elderly.^347^

The new drug fezolinetant, an antagonist of neurokinin B that acts directly on the hypothalamic thermoregulator center, has just been approved by the FDA to relieve VMS. Clinical trials have shown that the dose of 45 mg per day is effective to reduce VMS.^301^ It will soon be available in Brazil.

### 8.7.2. Alternative Pharmacological Therapies

Alternative pharmacological therapies, such as phytotherapy, have also been assessed to treat VMS. Isoflavones are non-steroid compounds found in plants and vegetables that have a phenolic ring and structure similar to that of estradiol, with high affinity and adhesion to hormonal receptors, and act as an estrogen agonist or antagonist. Studies on soy isoflavones, such as glycine max and *Trifolium pratense* (red clover), have shown conflicting results regarding the reduction of VMS as compared to placebo.^348^ Black cohosh (*Cimicifuga racemosa* or *Actae racemosa* L. ) is a phytotherapy agent whose mechanism of action has not been totally clarified and whose results are conflicting.^349^ Other compounds, such as *erva-de-são-joão*, ginkgo biloba, and ginseng, have no proven efficacy and are not recommended.^347^

### 8.7.3. Behavioral Therapies

Behavioral therapies, such as changes in lifestyle, physical exercises, yoga, and mindfulness, have not proven effective in reducing VMS. Cognitive-behavioral therapies, such as psychoeducation, understanding how emotions affect the perception of physical sensations, and avoiding triggers of VMS seem to reduce the discomfort associated with VMS.^347^

**Figure 8.2:**
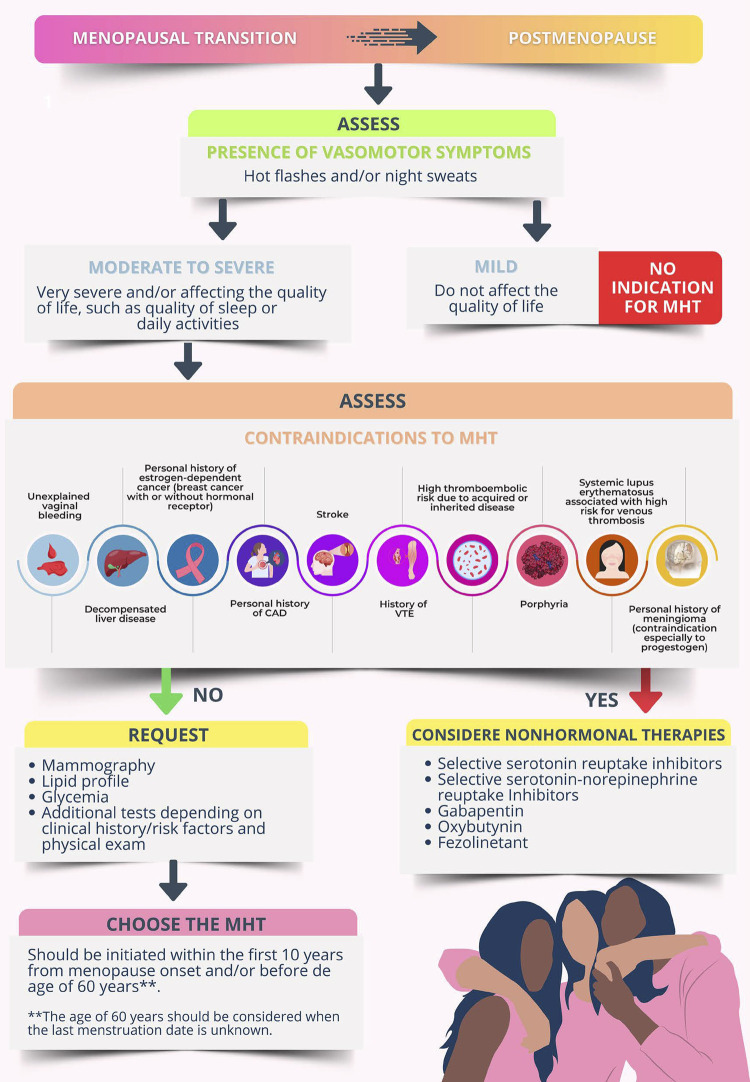
Flowchart for implementing the current recommendations for menopausal hormone therapy.

Figure 8.2 shows the flowchart for implementing the current recommendations for MHT. Chart 8.1 shows the types, doses, and routes of administration of estrogens and progestogens used in MHT.

**Chart 8.1:** Types, doses, and routes of administration of estrogens and progestogens used in menopausal hormone therapy. MHT: menopausal hormone therapy.

TYPES, DOSES, AND ROUTES OF ADMINISTRATION OF ESTROGENS USED IN MHT	
Oral route	
17-β-estradiol	0.5, 1.0, 2.0 mg/day
Estradiol valerate	1.0 - 2.0 mg/day
Transdermal route	
17-β-estradiol - gel	0.5, 0.75, 1.0, 1.5-3.0 mg/day
17-β-estradiol - patch	25, 50 mcg/day
Vaginal route	
Estriol cream	1.0 mg/g
Estradiol	10 mcg per pill
Promestriene	10 mg/g
TYPES, DOSES, AND ROUTES OF ADMINISTRATION OF PROGESTOGENS USED IN MHT	
Oral route	
Medroxyprogesterone acetate	2.5 - 10.0 mg/day
Cyproterone acetate	1.0 - 2.0 mg/day
Norethisterone acetate	0.1 - 1.0 mg/day
Nomegestrol acetate	2.5 - 5.0 mg/day
Dydrogesterone	5.0 - 10.0 mg/day
Drospirenone	2.0 mg/day
Micronized progesterone	100 - 200 mg/day
Transdermal route	
Norethisterone acetate	140 - 170 mcg/day
Vaginal route	
Micronized progesterone – soft gelatin capsules	100 - 200 mg/day
Intrauterine route	
HIUD-levonorgestrel	20 mcg/day

## 9. Menopause and Women in the Job Market – Difficulties and Opportunities for Improvement

### 9.1. Introduction

All women will enter menopause, and, in MT, the great majority will have several signs and symptoms, many of which hinder their quality of life.

According to global statistics, in 2020, there were 657 million women aged 45-59 years, 47% of whom were in the job market. Because the mean age at menopause is around 51 years, many working women are in that stage of life.^350^

In the current economy, the income from working women is necessary for not only them but their families as well; therefore, keep working during menopause is a reality for women. With the increase in women’s life expectancy and active age, many of them spend one third of their lives, as well as a significant amount of their professional years, in menopause and postmenopause.

Working is related to not only the obvious financial need, but to higher self-esteem, better health, and less psychological stress.

The impact of menopausal symptoms and related diseases on the work environment deserves higher professional attention than it currently gets. The negative effect of menopausal symptoms on the work ability of women in that stage of life is worth noting.^351,352^

There is a variety of experiences of menopausal women in the workplace influenced not only by the menopausal symptoms and context, but also by the physical and psychosocial characteristics of the work environment. These factors can affect not only their quality of life, but also their motivation and engagement in professional activities, daily performance, and relation with their employers.

In societies or environments in which menopause is considered a taboo, the lack of discussion about this topic and the stigma attached to it increase the burden of the symptoms to women.^353^

Studies have shown that the most worrisome symptoms to women in the workplace are the urinary symptoms, fatigue, difficulty sleeping, difficulty to concentrate, memory loss, sensation of dismay/depression, and reduced self-confidence.

Women with severe menopausal symptoms might quit their jobs or reduce their work hours, which negatively affects their income and safety later in life. For employers, this means loss of experienced personnel with valuable skills and talents.

Throughout the past decade, there has been an increased awareness of employers regarding menopause as a potential occupational health problem and the need for proper support at the workplace. Manager awareness about flexible working times has been considered the most beneficial workplace support.

It is necessary to recognize that menopausal symptoms can negatively impact work performance, and that the workplace can affect menopausal symptoms. Thus, it is fundamental to provide a work environment open to the women’s health needs, with an inclusive and supporting culture, without discrimination against those in this difficult life period.^350,353^

### 9.2. Impact of Menopausal Symptoms on Professional Life and How Women Cope with the Biological Changes in this Phase

#### 9.2.1. Signs and Symptoms and Professional Life in Menopause

Currently, one of the working groups that increases the most is that of women aged 50 years and older, with employment rates of people aged 50-64 years ranging from 55% to 67% in Europe, Australia, and the United States.^350^

**Figure 9.1:**
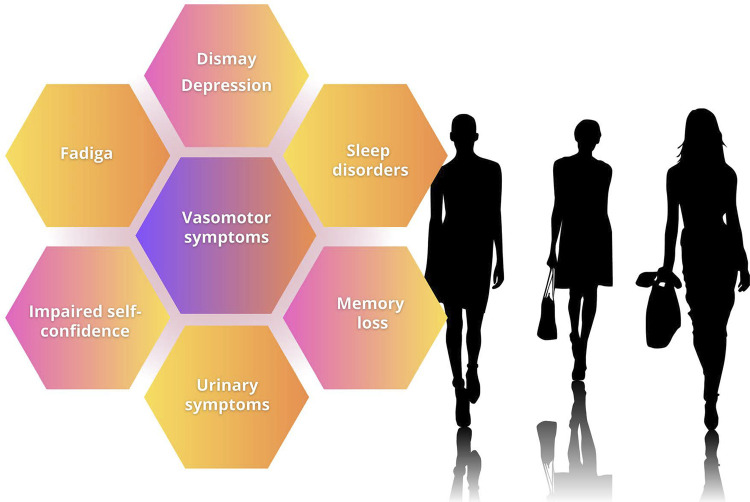
Menopausal symptoms with negative impact on professional performance.

Menopausal symptoms negatively impact working women, hindering their work performance, reducing job satisfaction, and jeopardizing individual professional well-being, which can result in direct and indirect costs for the employer and the State (Figure 9.1).

Loss of estrogen’s protective effect and its consequent changes lead to an increase in CVR during postmenopause, and more than half of the women develop SAH. Increases in cholesterol levels, body weight, and DM2 incidence are also frequent, contributing to elevate the CVR.^351^

Menopause is considered differently in different cultures, and more positively seen in those that value aging experience and wisdom. This has practical effects because women who consider menopause as something negative will have more symptoms.

In addition, menopause sometimes occurs in a difficult phase of life, when children leave their homes, many parents die, women have to take care of older parents of family members, and when age-related health problems occur. These situations increase the likelihood of developing depression, anxiety, chronic stress, and burnout syndrome.

It is worth noting that some health complaints associated with burnout, such as fatigue, sleep disorders, cognitive problems, such as difficulty to concentrate, memory loss, irritability, and emotional disorders, can be similar to menopausal symptoms, and identifying this is not always easy for women or even for health professionals.^351^

The most frequent symptoms in response to menopausal hormone changes are hot flashes, night sweats, sleep disorders, fatigue, muscle pain, vaginal dryness, urinary disorders, mood swings, headache, and poor concentration and memory. Peri- and postmenopausal insomnia and depression associate with productiveness loss and high costs for the employer.

The typical hot flashes usually appear suddenly and spread from the thorax to the neck, head, and arms, being secondary to increased blood flow to the skin, accompanied by increased heart rate, and followed by sweating in the upper part of the body. The increase in heart rate secondary to higher peripheral flow aims at maintaining proper cardiac output to maintain BP. The effect of the hot flashes on heart rate is even higher during hard working or under elevated temperatures.^354^ Occasional objective symptoms, such as increased menstrual flow and urinary incontinence, can occur, in addition to less specific complaints, such as cognitive and psychological symptoms.

A study with 407 working women in healthcare and social service, who answered a large questionnaire on menopause, has reported that the symptoms initiated between the ages of 46 years and 52 years in 43% of the women, and between 41 years and 45 years in 35% of them.^353^In addition, the physical and psychological effects of surgical menopause, observed in younger women, should be considered.^353^

#### 9.2.2. Menopause and the Work Environment

Usually, work improves women’s mental health, because it impacts positively on their self-esteem and health, and decreases psychological stress.^351^ Women that report good health are less influenced by menopausal symptoms at work.

The symptoms can start 4 years before and last for 4-8 years after menopause onset. Thus, the number of symptomatic working women, especially in work areas with prevalence of women, is significant, which has led, in recent years, to higher employers’ awareness on menopause as an occupational health concern and consequent need for support to these women.^353^

Menopause is rarely discussed in the workplace, due to taboos, which contributes to the lack of knowledge about this phase of life among women, health professionals, and employers. In a workplace where women are minority (police, military, industries), those difficulties to address menopause can be even more limiting. Thus, studies on the impact of menopause on women’s work and career are still scarce.

A study conducted in Holand has shown that women with severe menopausal symptoms have an 8.4-fold higher reduction in work ability than those paired for age but without those symptoms. In addition, there was a higher risk of absence from work, with higher financial loss.^355^

The acute effect of hot flashes can impact negatively on professional performance, affecting the ability to concentrate and complete complex cognitive tasks during hot flashes and sweats.

Women who have night sweats have their natural sleep cycle affected, sometimes with severe chronic consequences. Sleep deprivation leads to more sleep disorders and consequent insomnia and fatigue during the day. Thus, in the work environment, the impact of night sweats can manifest as reduced work ability and production, with higher likelihood of errors and consequent work accidents.^354^

The study by O’Neill, assessing the impact of menopausal symptoms on the work life of 407 women, has shown that 65% of the participants reported that the symptoms impacted their work performance, 35% stated that the symptoms influenced their career development decision, 18% reported occasional work absence due to their symptoms, 8% reported a reduction in their work times, 7% changed function, 6% stopped working night shifts, and 2% quit working due to the symptoms.^353^

The important negative impact of menopausal symptoms on the professional lives of this large number of women indicates the need for changes in their workplaces. These changes should aim to mitigate the problems of the work ability decline and work absenteeism related to those symptoms.

When asked about what should be changed in the workplace to reduce the impact of menopausal symptoms on work ability, one third of the women in the study by O’Neill mentioned their employer’s awareness would be the most important factor, followed by more flexible work schedules. In addition, they mentioned temperature and ventilation control, easy access to bathrooms and potable water fountains, and light uniforms. Regarding treatment offers, the most often mentioned were appointment with general practitioners, hormonal therapy use, and antidepressants. Another finding of that study was that neurocognitive and psychological symptoms had a higher impact, while the VMS had a smaller influence on those women’s work.^353^

Thus, health professionals and employers should be able to recognize that menopausal symptoms can affect working women’s well-being, and, thus, the quality of their lives and work ability, which will lead to a reduction in the working hours, underemployment or even unemployment, and their deleterious financial consequences.^350^

## 9.3. Employers and Menopause – Improvement Opportunities

In 1971 in Great Britain, there were 3 million employed women aged 45-59 years, and, in 2001, they were 3.9 million, representing 45% of the over 50-year-old workforce.^356^

Menopause is not a uniform experience among working women, and there is large variation in the prevalence of symptoms that create difficulties at work (25% to 65%), according to different studies.

Symptomatic women have higher levels of absenteeism (medical leave) and higher frequency of outpatient appointments. The costs due to work disability increase significantly in women with severe symptoms as compared to those with mild symptoms, and VMS, insomnia, sleep disorders, and psychosocial symptoms have the highest impact.^357^

The recently published results from the *Health and Employment after Fifty Study (HEAF)* have shown that, while the most frequently mentioned symptoms by peri- and postmenopausal working women were VMS (91.7%), and sleep (68.2%), psychological (63.6%), and urinary (49.1%) disorders, the symptoms that most impacted the work ability were the psychological ones (depression, irritability, anxiety, crying), severe headache, and joint pains.^358^In that study, the RFs for difficulty coping with menopausal symptoms at work were financial deprivation, poorer self-rated health, depression, and adverse psychosocial occupational factors.^358^The authors have concluded that inequality also affects menopausal working women, requiring higher awareness of employers of all sectors, and special attention to vulnerable women (with economic difficulties, jobs in which they feel insecure, unappreciated, or dissatisfied), because they are at higher risk.^358^

Problems with supervisors or colleagues, such as the perception of lack of support or the perceived probability that the mention to menopause will cause embarrassing, irrelevant, or discriminatory responses, have also been reported as the cause of difficulties and led to women’s lack of will to reveal their menopausal status.^359^

According to the study by Reynolds, women experience VMS at work, with distress worsening during formal meetings, in closed and hot spaces, and in the presence of men. The approach to this problem has been seen as an individual rather than organizational responsibility. Reynolds has suggested that counsellors and health occupational agents in organizations should raise awareness about the women’s needs at the management level, with sensitization to the potential impact of hot flashes on work experiences and preparing to provide support and counseling. Simply making controllable fans and heating systems available is a practical help for the most common problems.^360^

If the problem is seen as personal, revealing it is allowed when women have positive attitudes towards aging or have empathetic colleagues. However, when the problem is considered organizational, discussing and negotiating the underlying needs might generate alternatives to solve the problems, such as flexible schedules, change to part-time schedules for a while, occasional remote work, thermostat adjustments, discussion of annual vacations, change of the office site to well-ventilated and illuminated areas, closer to bathrooms, as well as talks with supervisors seeking creative solutions.^359^

Organization members sometimes become aware of a woman’s age through menopausal symptoms. Employers have taken too long to recognize that menopausal women might need special considerations. Organizations should try to reduce the menopause-related stigma and present it as an asset rather than a liability.

In their review, Jack *et al.*^357^ have suggested four recommendations:

1. Employers should review the scope of their policies, practices, and relevant activities regarding health and safety at work, as well the human resources, considering the national legal requirements to provide safe, healthy, and discrimination-free environment for working women in perimenopause, adopting a holistic approach depending on the company’s sector;
2. As part of their responsibilities regarding safety and health at work, employers should perform risk assessments of symptomatic women to identify the reasonably necessary adaptations that can be made in the physical and psychosocial work environment. This will depend on the type/function of the work and industry;
3. Employers should develop programs of health promotion that include information on menopause, aging, and health, enabling women to self-manage symptoms (for example, through dietary changes, stress management, development of positive attitudes regarding aging and menopause) and providing formal and informal social support (for example, through a women’s network or through lunch-time meetings);
4. A variety of policies and practices for training and development of the organization and human resources should be implemented to foster more friendly work environments, positive supervisor/subordinate relations, and support for menopausal women.

## 9.4. How to Improve Work Conditions

The past decade witnessed an increase in employers’ awareness on menopause as a potential occupational health problem, as well as on the need to provide proper support in the workplace. However, efforts in this direction are scarce, with almost no dialogue between employer/manager and workers in most organizations.

Employers should promote an institutional culture in which menopausal symptoms can be discussed and managers and supervisors are educated on menopausal topics and trained to be able to engage in supportive conversations with employees.

A study performed at the Mayo Clinic has shown that the missed days of work attributed to menopausal symptoms represent an annual cost of approximately 1.8 billion dolars.^361^

Temperature control (72%), flexible working hours, including work sharing, time off for medical appointments, regular intervals (58%), seminars on healthy aging (50%), flexible workspaces, such as home office, office change (50%), physical activity programs (46%), and table fans (45%) would be the most popular measures reported.^353^

**Chart 9.1:**
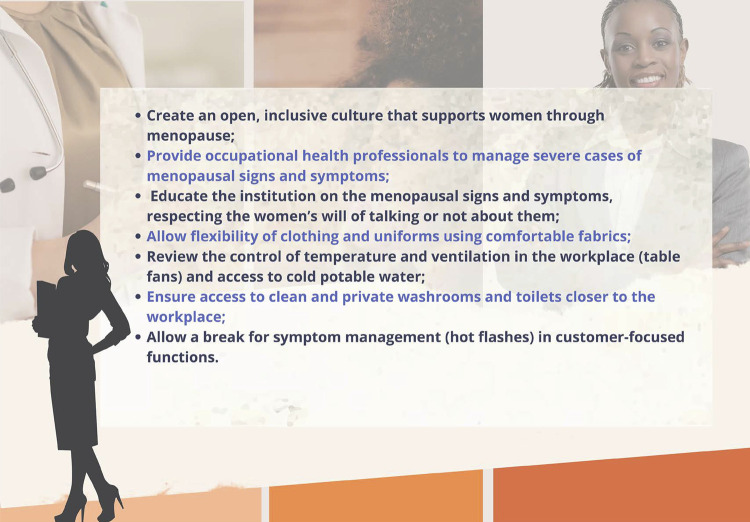
Practical actions about menopause to be adopted in the workplace by employers.

Menopause can be considered a taboo, not to be discussed in the work environment, although many workers might be affected. Being able to have proper conversations and adjust the work environment to the needs of perimenopausal women will positively impact on their quality of life, their engagement, performance, and professional motivation. Some practical actions to be adopted in the workplace by managers and supervisors are shown in Chart 9.1.^350^

## 9.5. Conclusions

Studies have assessed the frequency of menopausal symptoms rather than their impact on work, or have focused on grouped results, such as quality of life and survival strategies, having forgotten the effects on careers and professional well-being.

Women are a large part of the global workforce. Therefore, it is necessary to make the work environment more favorable to menopausal women to improve their well-being and ability to work, thus, ensuring that more people reach retirement with the possibility to maintain their contributions to pensions and savings, sufficient for proper income and safety in life.

## 10. Menopause and Postmenopause in Latin America – Current Situation, Chalenges, and Opportunities for Intervention

### 10.1. Introduction

Latin America (LA) has more than 20 million km^2^ and comprises 20 countries from Mexico to the South of South America: Argentina, Bolivia, Brazil, Chile, Colombia, Costa Rica, Cuba, Dominican Republic, Ecuador, El Salvador, Guatemala, Haiti, Honduras, Mexico, Nicaragua, Panama, Paraguay, Peru, Uruguay, and Venezuela.^362-364^

The region encompasses several cultures, a variety of idioms (Spanish, which predominates, Portuguese, French, English, Dutch, and several native languages), ethnicities (white, black, native), climates, religions, and traditions. Although sharing the same geographical region and common historical elements, each country has characteristics that make them unique.^362-364^

The region underwent a process of late industrialization and rapid and accelerated urbanization, the economy of the countries being based on exploration and exportation of natural resources, with the tertiary sector playing an important role.^362-364^

Latin America has a population estimated at 659 744 000 inhabitants (ONU, 2022), of which, approximately 80% live in the cities, the largest being São Paulo (Brazil), Mexico City (Mexico), and Buenos Aires (Argentina).^362-364^Women represent 51% of the total population of LA.^364^

The analysis of the life expectancy and causes of death of 363 cities from nine countries in LA has shown a large variation in those indicators among the countries and within each one.^365^Life expectancy at birth in LA ranges from 74 years to 83 years and 63 years to 77 years, for women and men, respectively. Some countries, such as Panama, Costa Rica, and Chile, have higher life expectancy levels, but there is large variation among the cities within each country, which can reach 7-10 years, such as Mexico, Brazil, Colombia, and Peru.^365^In addition, a large heterogeneity has been observed in mortality among the countries in LA and within each country, although chronic diseases (CVDs and others) remain the most common cause of death, representing, on average, 57.7% of total mortality. In LA, on average, cancer accounts for 16.2% of mortality in general, the communicable, maternal, neonatal, and nutritional causes for 14.4%, and external causes for 11.6%.^365^In addition, the results of that study have shown that a higher level of education, access to water and basic sanitation, and living in less crowded cities are predictive of higher life expectancy, of a relatively lower proportion of deaths from communicable, maternal, neonatal, and nutritional diseases, as well as of a higher proportion of deaths from CVD, cancer, and other chronic diseases in men and women.^365^

Some studies have shown that some women perceive menopause as a natural stage of life, with no negative implications, while, for many others, the hormonal changes of that phase can generate symptoms that affect their physical, emotional, mental, and social well-being.^15,366-368^

The hormonal changes of menopause determine several changes in female health, such as increased bone and cognitive loss, in addition to an elevation in the CVR in postmenopause, due to the higher prevalence of IHD, stroke, peripheral vascular disease, thromboembolic phenomena, AF, HF, and CV mortality.^15,366-368^Those risks are not reduced by MHT with synthetic estrogens/progestogens.^179,255^

It is worth noting that there is a secular trend of increase in the mean menopause age of women living in high-income countries. However, in low/middle-income countries, a clear increase in the prevalence of POF (before age 40 years) and early menopause (ages 40-44 years),^369^ both currently considered RFs for CVD and CV mortality, has been observed.^15,368^

### 10.2. Woman and menopause in Latin America

#### 10.2.1. Natural Menopause, Premature Ovarian Failure, and Early Menopause

Castelo-Branco *et al*.,^370^ in 2006, assessing 17 150 healthy women aged 40-59 years, in 47 cities from 15 Latin American countries, have reported median age at menopause for the entire sample of 48.6 years (43.8 years to 53 years).^12^ They have reported that 49-year-old women living in cities at 2000 meters or more above sea level and those with lower educational level or living in countries with lower income were more likely to have an early menopause onset.^370^

A meta-analysis published in 2014 by Schoenaker *et al.,*^371^ including the study by Castelo-Branco *et al.*,^370^ has shown that the mean age at natural menopause in LA was 47.24 years (45.9 years to 48.6 years).^371^ In that meta-analysis, performed based on studies across 24 countries from the six continents, the authors have concluded that the mean age at natural menopause was lower in countries from Africa (48.38 years), LA (47.24 years), Asia (48.75 years), and Middle East (47.37 years), and higher in Europe (50.54 years), Australia (51.25 years), and the United Sates of America (49.11 years), with a mean of 48.8 years (46 years to 52 years) for the six continents.^371^ In addition, the meta-analysis has shown that smoking and lower educational and occupational levels associated with earlier age at natural menopause.^371^

Leone, Brown, and Gemmill^369^ have conducted a study using 302 standardized household surveys from 1986 to 2019, with women aged 15-49 years, in 76 low/middle-income countries, in five geographical regions (Central Asia; LA and the Caribbean; Northern Africa/Western Asia/Europe; Southern/Southeastern Asia; and Sub-Saharan Africa). Those authors have reported an increasing prevalence of POF and early menopause in low/middle-income countries, particularly in Sub-Saharan Africa and Southern/Southeastern Asia, and that these regions also had a reduction in mean age at menopause, with high variation among the continents. Table 10.1 shows the results for the regions studied.

**Table 10.1:** Prevalence of premature ovarian failure and early menopause and mean age at menopause onset in different geographical regions, in women aged 15-49 years (modified from Leone, Brown, and Gemmill369).

REGIONS	PREMATURE OVARIAN FAILURE (%)	EARLY MENOPAUSE (%)	MEAN AGE AT MENOPAUSE ONSET
Central Asia	1.2	1.6	45.3
Latin America and the Caribbean	1.5	1.9	44.4
Northern Africa/Western Asia/Europe	0.1	1.4	44.7
Southern/Southeastern Asia	2.7	4.5	43.7
Sub-Saharan Africa	0.9	2.4	44.1

As compared to previous studies, only the Northern Africa/Western Asia/Europe region had a reduction in the prevalence of POF/early menopause and elevation in the mean age at menopause.^369^

These results call attention to the fact that LA is among the geographical regions with the lowest mean age at menopause onset and highest prevalence de POF and early menopause, when compared to regions with higher development and higher income. Considering that menopause (natural, POF, or early menopause) is currently a CVRF, this profile can contribute, along with other RFs, to increase the CVR of Latin American women.

#### 10.2.2. Symptoms and Quality of Life in Menopausal Transition in Latin American Women

Menopausal transition can cause several symptoms that hinder women’s quality of life, such as VMS (hot flashes and sweats, usually at night), insomnia, mood swings (with higher propension to depression), irritability, mental confusion, genital changes (vaginal dryness, pain during sexual activity, libido decline), urinary tract symptoms, muscle/joint pains, and palpitations.^372,373^ The VMS can last for 7-9 years or more.^372^

A – Vasomotor Symptoms

In a systematic review of studies performed in Europe, North America, LA, and Asia, published between 1966 and 2009, Palacios *et al*. have reported that, in addition to the geographical differences observed in the mean age at menopause onset, there are differences in the frequency of the symptoms reported by women, but the VMS are among those of higher prevalence in all regions studied (Europe, 74%; North America, 36-50%; LA, 45-69%; Asia, 22-63%).^373^

Using the Menopause Rating Scale and an itemized questionnaire containing sociodemographic data, Blümel *et al.* have assessed 8373 women, aged 40-59 years, from 22 health centers, in 18 cities from 12 countries in LA. Those authors identified VMS in 55% of the sample, and the symptoms were more severe in 9.6%.^374^ Logistic regression analysis showed that the presence of severe psychological/urogenital symptoms, lower educational level, natural perimenopause/postmenopause status, nulliparity, surgical menopause, and living at high altitude were significant RFs for severe VMS.^374^

Sánchez-Zarza *et al*. have recently published a cross-sectional study in which 216 women aged 40-60 years, living in urban areas of the city of Asunción, Paraguay, were surveyed with the 10-item Cervantes Scale and a general questionnaire (personal and partner data). According to the 10-item Cervantes Scale, the three most prevalent symptoms were muscle/joint pains (70,8%), anxiety and nervousness (70.8%), and VMS (54.2%).^375^

In a population-based study with 1500 Brazilian women aged 45-65 years, Pompei *et al.* have reported that the median age at menopause onset was 48 years (45 years to 51 years), with no difference between economic levels. Menopause-related symptoms were present in 87.9% of those in MT, and the VMS appeared earlier.^376^

Aiming to assess the prevalence and impact of moderate/severe VMS in postmenopause, a cross-sectional study has been performed with 12 268 women aged 40-65 years in Brazil, Canada, Mexico, and four Nordic countries (Denmark, Finland, Norway, and Sweden). The prevalence of moderate/severe VMS was 15.6%, higher in Brazil (36.2%) and lower in Nordic Europe (11.6%). The VMS impacted the quality of life in all domains assessed.^377^

B – Sleep Disorders

In a study assessing 6079 women aged 40-59 years, from 11 Latin American countries and using several investigation tools, Blümel *et al.* have found that 56.6% of the sample had insomnia or poor quality of sleep or both, and that the prevalence of insomnia increased with age and from pre- to late postmenopause.^378^ The logistic regression analysis identified that age, chronic diseases, alcohol abuse, anxiety, depression, VMS, and drug use (hypnotics and MHT) contributed to the occurrence of sleep disorders, while higher educational level associated with less insomnia and better quality of sleep.^378^

C – Muscle/Joint Pains

Blümel *et al*.,^374^ using that same sample of Latin American women, have assessed the relation of muscle/joint pains with other menopausal complaints.^379^ Those authors reported that 63% of the sample had such symptoms, and 15.6% were classified as severe/very severe. The logistic regression analysis identified that age, smoking, lower educational level, VMS, POF, postmenopause, psychiatric consultation, and the use of psychotropic drugs were significantly related to higher severity of symptoms, while self-perception of health, private access to healthcare, and use of MHT were significantly related to less severe symptoms.^379^

D – Sexual Dysfunction

By use of the Female Sexual Function Index to investigate sexual dysfunction in 7243 healthy women aged 40-59 years, users of 19 health systems from 11 Latin American countries, Blümel *et al.* have shown the occurrence of sexual dysfunction in 56.8% of the sample.^380^ The logistic regression analysis showed that the RFs for sexual dysfunction were poor vaginal lubrication (most important), use of alternative menopausal therapies, and the partner’s sexual dysfunction. Women’s higher educational level, partner’s faithfulness, and access to private healthcare were identified as protective factors.^380^

Using that same tool (Female Sexual Function Index), Cruz, Nina & Figuerêdo have assessed the association between sexual dysfunction and intensity of postmenopausal symptoms (assessed by use of the Blatt-Kupperman Index) in Brazilian women aged 40-65 years, treated at a public hospital.^381^ The sexual dysfunction prevalence was 58.73%, identified in 100% of the women with severe postmenopausal symptoms, in 70.59% of those with moderate symptoms, and in only 9.09% of those with mild symptoms.^381^

E – Other Symptoms

Blümel *et al*.^374^ have shown that, women with VMS are five times more likely to experience chest discomfort, four times more likely to experience depressive mood, sleep changes, sexual dysfunction, anxiety, physical and mental exhaustion, and vaginal dryness, and three times more likely to experience irritability, muscle/joint pains, and urinary dysfunction.^374^

F – Quality of Life

Using the same sample,^374,378^ Blümel *et al*.^380^ have assessed the quality of life and its impairing factors. The prevalence of women with moderate to severe symptoms that hinder quality of life was higher than 50% in all countries, but Chile and Uruguay achieved the highest scores (80.88% and 67.4%, respectively). The logistic regression analysis identified that the impaired quality of life of Latin American menopausal women was associated with the use of alternative menopausal therapies, use of psychiatric drugs, being postmenopausal, being 49 years or older, living at high altitudes, and having a partner with erectile dysfunction or premature ejaculation. Better quality of life was associated with living in a country with lower income, being on MHT, and engaging in healthy habits.^382^

Table 10.2 shows the prevalence of major menopausal symptoms, their RFs and protective factors in Latin American women.

**Table 10.2:** Prevalence of menopausal symptoms, their worsening and protective factors in Latin American women373,374,378-380,382

SYMPTOMS/QUALITY OF LIFE	PREVALENCE	WORSENING FACTORS	PROTECTIVE FACTORS
Vasomotor symptoms	45-69%; 55%	Severe psychological/urogenital symptoms, lower educational level, natural peri- and postmenopause, nulliparity, surgical menopause, living at high altitudes	Use of MHT
Sleep disorders	56.6%	Older age, chronic diseases, alcohol abuse, anxiety, depression, VMS, drug use (hypnotics and MHT)	Higher educational level associated with less insomnia and better quality of sleep
Muscle/joint pain	63%	Older age, smoking, lower educational level, VMS, premature menopause, postmenopause, psychiatric consultation, and use of psychotropic drugs	Self-perception of health, private access to healthcare, and use of MHT
Sexual dysfunction	56.8%	Reduced vaginal lubrication (most important), use of alternative menopausal therapies, and partner’s sexual dysfunction	Higher educational level, partner’s faithfulness, and access to private healthcare
Quality of life	50% with severe impairment	Use of alternative menopausal therapies, use of psychiatric drugs, being postmenopausal, being 49 years or older, living at high altitudes, partner with erectile dysfunction or premature ejaculation	Living in a country with lower income, being on MHT, and engaging in healthy habits

MHT: menopausal hormone therapy; VMS: vasomotor symptoms.

## 10.3. Cardiovascular Risk Factors

The studies performed by the REDLINC group from 2004 till now, resulting in seven investigation projects and 18 publications on several aspects of menopause in Latin American women, have evidenced the prevalence of traditional and emerging CVRFs as shown in Table 10.3.^383-386^

**Table 10.3:** Prevalence of cardiovascular risk factors in Latin American menopausal women383-386

CARDIOVASCULAR RISK FACTOR	PREVALENCE (%)
Sedentary lifestyle	63.9
Anxiety	59.7
Depression	46.5
Metabolic syndrome	35
Systemic arterial hypertension	22.9
Obesity	18.5
Smoking	11.3
Diabetes mellitus	8.6
Early menopause	1.9
Premature ovarian failure	1.5

The high prevalence of CVRFs poses a higher CVR to Latin American peri- and postmenopausal women, which might contribute to the high CV mortality currently observed in that geographical region.^14^ However, that is also an opportunity to control the RFs and reduce the CVR in that phase of life.^13,141,173,387-389^

## 10.4. Medicamentous and Non-medicamentous Treatment

There is evidence that, in LA, MHT is prescribed for 12.5% of menopausal women, the major route of administration being oral (43.7%), followed by transdermal (17.7%).^179,347,390^ The use of MHT associated with the patient having a positive perception of MHT, being postmenopausal, and having a higher socioeconomic level.^390^ In the group of those who never used MHT, 28% reported lack of a medical prescription as the major reason, followed by absence of symptoms (27.8%). Of those reporting lack of a medical prescription as the major reason for not being on MHT, 30.6% had severe menopausal symptoms (Menopause Rating Scale total score > 16).^390^ The use of alternative therapies was reported by 19.5% of the women studied, 35.1% of whom mentioned experiencing severe menopausal symptoms as compared to 22.5% of MHT users.^390^

A survey using a self-administered and anonymous questionnaire conducted with 2154 gynecologists (55.5% of the male sex, 20.3% faculty members, and 85% with a partner) in 11 countries of LA,^391,392^ 85.3% of whom responded to the survey (n = 1837), has shown that:

- 85.4% of the gynecologists responded they would use MHT if they had menopausal symptoms (81.8% female gynecologists) or would prescribe for their partners (88.2% male gynecologists).
- The perceived risk related to MHT use (on a 0-to-10 scale) was higher among female than male gynecologists (4.06 ± 2.09 *versus* 3.83 ± 2.11, respectively); the two major risks reported were thromboembolism (women 33.6% *versus* men 41.4%, respectively) and breast cancer (women 38.5% *versus* men 33.9%, respectively).
- Overall, gynecologists reported prescribing MHT for 48.9% of their symptomatic patients (women 47.3% *versus* men 50.2%, respectively), while 86.8% prescribed nonhormone and 83.8% alternative therapies for the management of menopause.
- Older gynecologists and academic professionals prescribed MHT more often.

The authors have concluded that, although Latin American gynecologists support MHT use for themselves and their partners, this is not reflected in their clinical practice.^391,392^ There is no clinical trial on the use of other drugs to control menopausal symptoms in LA as a whole.

Several interventions related to alternative medicine, such as body-mind practices (hypnosis, cognitive-behavioral therapies, relaxation, biofeedback, meditation, aromatherapy), the use of natural products (herbs, vitamins, minerals, dietary supplements) and others (traditional Chinese medicine, reflexology, acupuncture, homeopathy), have been used to control menopausal symptoms, many of which have been assessed on clinical trials somewhere else, but there is no specific study in LA.^393,394^

## 10.5. Challenges and Opportunities for Intervention

The combination of increased life expectancy at birth, reduction in the mean age at natural menopause, and increased prevalence of POF/early menopause has contributed to increase the number of Latin American women (51% of the population) experiencing MT and its negative impact on their quality of life (in all domains), with expressive loses in the personal, social, and economic levels, as well as elevation in CV morbidity and mortality (major cause of death worldwide currently).^14,365,369-371^

According to Faubion and Schufelt,^10^ after the gap in menopausal treatment left by the results of the HERS and WHI studies,^1,395^ the topic has received increasing attention by the health services, media (newspapers, blogs) and even celebrities, because of multiple reasons, such as the projection of a market of $ 600 billion involving several products and the arrival of new generations of women (Y, millennials) who choose not to suffer silently the deleterious consequences of menopause.^10^

**Figure 10.1:**
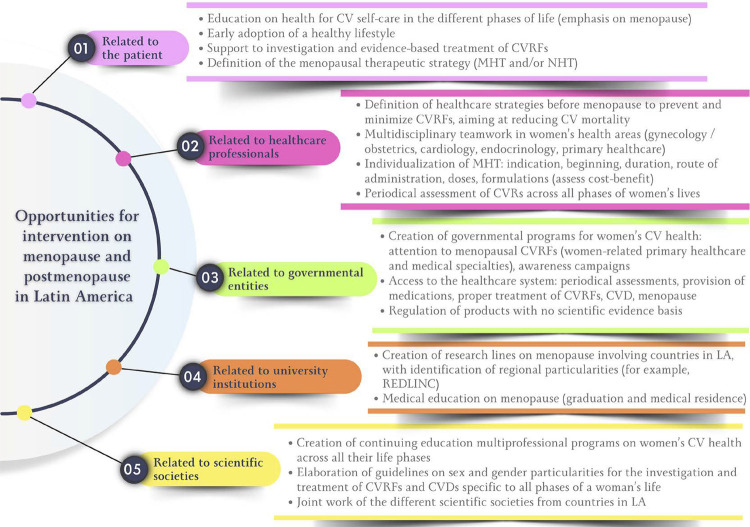
Interventions to improve menopause-related situations. CV: cardiovascular; CVR: cardiovascular risk; CVRF: cardiovascular risk factors; LA: Latin America; MHT: menopausal hormone therapy; NHT: nonhormone therapy.

Considering the worldwide and Latin American scenarios regarding menopause, Figure 10.1 lists the opportunities to intervene to improve the currently observed situation.

**Figure 10.2:**
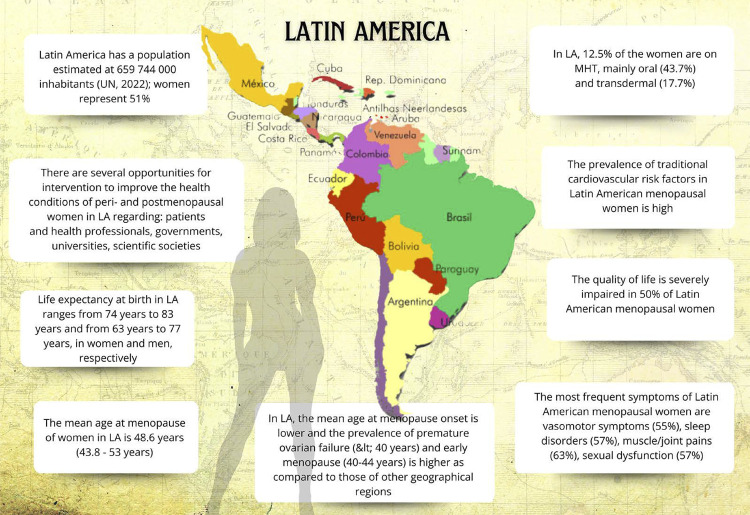
Latin American demographic and menopause data. LA: Latin America; MHT: menopausal hormone therapy.

Figure 10.2 summarizes the Latin American demographic and menopausal data.
